# Should the Identification Guidelines for Siamese Crocodiles Be Revised? Differing Post-Occipital Scute Scale Numbers Show Phenotypic Variation Does Not Result from Hybridization with Saltwater Crocodiles

**DOI:** 10.3390/biology12040535

**Published:** 2023-03-31

**Authors:** Nattakan Ariyaraphong, Wongsathit Wongloet, Pish Wattanadilokchatkun, Thitipong Panthum, Worapong Singchat, Thanyapat Thong, Artem Lisachov, Syed Farhan Ahmad, Narongrit Muangmai, Kyudong Han, Prateep Duengkae, Yosapong Temsiripong, Kornsorn Srikulnath

**Affiliations:** 1 Animal Genomics and Bioresource Research Unit (AGB Research Unit), Faculty of Science, Kasetsart University, 50 Ngamwongwan, Chatuchak, Bangkok 10900, Thailand; nattakan.ari@ku.th (N.A.); gamewongsathit@gmail.com (W.W.); pish.wa@ku.th (P.W.); thitipong.pa@ku.th (T.P.); worapong.singc@ku.ac.th (W.S.); thongthanyapat@gmail.com (T.T.); lisachev@bionet.nsc.ru (A.L.); syedfarhan.ah@ku.ac.th (S.F.A.); ffisnrm@ku.ac.th (N.M.); kyudong.han@gmail.com (K.H.); prateep.du@ku.ac.th (P.D.); 2 Special Research Unit for Wildlife Genomics (SRUWG), Department of Forest Biology, Faculty of Forestry, Kasetsart University, 50 Ngamwongwan, Chatuchak, Bangkok 10900, Thailand; 3 Laboratory of Animal Cytogenetics and Comparative Genomics (ACCG), Department of Genetics, Faculty of Science, Kasetsart University, 50 Ngamwongwan, Chatuchak, Bangkok 10900, Thailand; 4 Department of Fishery Biology, Faculty of Fisheries, Kasetsart University, Bangkok 10900, Thailand; 5 Department of Microbiology, Dankook University, Cheonan 31116, Republic of Korea; 6 Bio-Medical Engineering Core Facility Research Center, Dankook University, Cheonan 31116, Republic of Korea; 7 R&D Center, Sriracha Moda Co., Ltd., Sriracha 20230, Thailand; yosapong@srirachamoda.com; 8 Center of Excellence on Agricultural Biotechnology (AG-BIO/PERDO-CHE), Bangkok 10900, Thailand; 9 Center for Advanced Studies in Tropical Natural Resources, National Research University-Kasetsart University, Bangkok 10900, Thailand

**Keywords:** Siamese crocodile, saltwater crocodile, introgression, hybridization, post-occipital scutes

## Abstract

**Simple Summary:**

Morphological divergence between Siamese and other crocodiles has been identified by size, number of scales, and patterns of cervical squamation with post-occipital scutes (P.O.). However, a large variation of P.O. has been observed in captive Siamese crocodiles in Thailand, leading to questions about possible crocodile hybrids. The genetic diversity and population structure of Siamese crocodiles were studied using mitochondrial DNA D-loop and microsatellite genotyping. The STRUCTURE plot revealed numerous distinct gene pools, indicating that the crocodiles in each farm descended from distinct lineages. Researchers also discovered evidence of introgression in several individual crocodiles, implying that Siamese and saltwater crocodiles may have hybridized. A schematic protocol for screening hybrids was proposed based on patterns observed in phenotypic and molecular data.

**Abstract:**

Populations of Siamese crocodiles (*Crocodylus siamensis*) have severely declined because of hunting and habitat fragmentation, necessitating a reintroduction plan involving commercial captive-bred populations. However, hybridization between Siamese and saltwater crocodiles (*C. porosus*) has occurred in captivity. Siamese crocodiles commonly have post-occipital scutes (P.O.) with 4–6 scales, but 2–6 P.O. scales were found in captives on Thai farms. Here, the genetic diversity and population structure of Siamese crocodiles with large P.O. variations and saltwater crocodiles were analyzed using mitochondrial DNA D-loop and microsatellite genotyping. Possible crocodile hybrids or phenotypic variations were ascertained by comparison with our previous library from the Siam Crocodile Bioresource Project. Siamese crocodiles with <4 P.O. scales in a row exhibit normal species-level phenotypic variation. This evidence encourages the revised description of Siamese crocodiles. Moreover, the STRUCTURE plot revealed large distinct gene pools, suggesting crocodiles in each farm were derived from distinct lineages. However, combining both genetic approaches provides evidence of introgression for several individual crocodiles, suggesting possible hybridization between Siamese and saltwater crocodiles. We proposed a schematic protocol with patterns observed in phenotypic and molecular data to screen hybrids. Identifying non-hybrid and hybrid individuals is important for long-term in situ/ex situ conservation.

## 1. Introduction

Siamese crocodile (*Crocodylus siamensis*, Schneider, 1801) [1] is a freshwater species found in a wide range of lowland freshwater habitats including slow-moving rivers, streams, lakes, seasonal oxbow lakes, marshes, and swamps in mainland Southeast Asia, including Cambodia, Lao PDR and Thailand and on some islands of Indonesia and Malaysia [2,3,4,5,6]. This medium-sized crocodylian has a total length of less than 3.5 m [7]. However, its historical population distribution has decreased by 20% globally, with only 11% of its habitat range in nationally protected areas [8]. The Siamese crocodile was listed as a critically endangered species on the International Union for Conservation of Nature (IUCN) Red List and in Appendix I of the Convention on International Trade in Endangered Species of Wild Fauna and Flora (CITES) in 1996 to aid conservation efforts. In Thailand, Siamese crocodiles are widely distributed in lowland regions; however, most populations have been extirpated because of hunting, habitat loss, and collection to stock commercial crocodile farms over the last 40 years [8,9]. The severe decline of Siamese crocodile populations has led to there being fewer than 20 wild individuals in Khao Ang Rue Nai Wildlife Sanctuary (13°13′2.13″ N, 101°42′37″ E), Kaeng Krachan National Park (12°54′1.9″ N, 99°38′13.98″ E), Namnao National Park (16°57′57.65″ N, 101°30′28.08″ E), Yod Dom Wildlife Sanctuary (14°26′6.06″ N, 105°6′ 1.2″ E), and Bueng Boraphet (15°41′2.67″ N, 100°14′59.07″ E) [6,10]. To restore Siamese crocodile wild populations, it is essential to reintroduce captive-bred individuals and implement in situ/ex situ management practices. [11]. By contrast, 1.3 million Siamese crocodiles were present on 1400 farms in 2020, and crocodile farming now accounts for approximately 1% of Thailand’s agricultural income [12]. However, the occurrence of hybridization between Siamese and saltwater crocodiles (*C. porosus*, Schneider, 1801) [13] is bidirectional between males and females of parental species in captivity. Interspecific hybridization frequently occurs in Southeast Asia due to the keeping of both species together in captivity, rather than from the wild [11,14]. Both F_1_ hybrid and backcross crocodiles are fertile and reportedly grow faster than either parental species [13]. The genetic integrity of the species is at risk, which could harm conservation management. Alien outbreeding depression hybrids must be identified from the parental species before the reintroduction program or to improve genetic diversity in the wild. Using an effective genetic diagnosis approach, a genetically diverse captive population of pure Siamese crocodiles was identified while hybrids were differentiated from the parental species. Siamese crocodile sources serve as critical genetic resources for reintroduction efforts [11,15].

The morphological divergence between Siamese and other crocodiles (*Crocodylus* spp.) has been identified by size, the number of scales, and patterns of cervical squamation with post-occipital scutes (P.O.) [16]. The P.O.s of Siamese crocodiles show one row with 4–6 scales and several small scales, but no P.O. is seen in saltwater crocodiles. However, a large variation of P.O., ranging from two to six scales, has been observed in captive Siamese crocodiles in Thailand. This leads us to question whether possible crocodile hybrids remain in captivity, or whether these are actual phenotypic variations of Siamese crocodiles. A genetic approach to identifying Siamese and saltwater crocodiles was developed together with morphological observations in our previous study [11,15] but the P.O. pattern of each crocodile was not photo-recorded as evidence in our library report. In this study, to test these hypotheses, the genetic diversity and structure of Siamese and saltwater crocodile populations were assessed by screening the gene pool using 22 microsatellite markers and mitochondrial (mtDNA) D-loop sequences coupled with each crocodile photo record. MtDNA and nuclear DNA microsatellites are molecular genetic markers that can identify population diversity, origins of individuals, and hybrids along with their parents, especially in crocodiles [11,15]. Results were compared with those of the large gene pool library under “the Siam Crocodile Bioresource Project” from our previous study [11]. These findings provide pivotal information for prospective reintroduction programs and in situ/ex situ management.

## 2. Materials and Methods

### 2.1. Specimen Collection and DNA Extraction

A total of 136 Siamese and 29 saltwater crocodile specimens were collected from 4 captive locations under the auspices of the Thai Crocodile Farm Association (TCFA) and in accordance with CITES regulations for the leather and food industries. Table S1 provides detailed information on the sampled individuals. Scale samples were collected from the tail of captive crocodiles registered at four crocodile farms in Chonburi (CB) (13°09′06.57″ N, 101°28′36.01″ E), Nakhon Ratchasima (NR) (14°57′21.19″ N, 101°28′36.01″ E), Chainat (CN) (15°15′10.44″ N, 100°02′38.27″ E) and Nakhon Pathom (NP) (13°43′20.25″ N, 100°15′20.83″ E) between January and August 2022. A piece of scale clipped from the tail of each specimen was collected as a DNA source. Permission was granted by the farm owners and the TCFA and also from unnamed crocodile farms. Individuals were classified as Siamese or saltwater crocodiles based on external morphological observation [17,18] and photographed. The dataset comprised photo images of 165 individual crocodiles, each captured in 30–50 different postures to minimize testing redundancy and bias. The DNA extraction and quality assessment were performed using the same methods as in previous studies [19] (Supplementary Data S1). All experimental procedures and animal care were carried out in compliance with the Regulations on Animal Experiments at Kasetsart University and approved by the Animal Experiment Committee under Approval No. ACKU64-SCI-011.

### 2.2. Microsatellite Genotyping and Microsatellite Data Analysis

Twenty-two microsatellite primer sets, developed originally from saltwater crocodiles (Table S2) [20,21], were used for the genotyping of all crocodile individuals. The genotypic data resulting from this study were deposited in the Dryad Digital Repository Dataset (https://datadryad.org/stash/share/s4zREYQ1AUUXTsaIpk0r1HSdkYljvu8yvJOVR143K7Y, accessed on 18 February 2023). We used the same methods as previous studies for PCR amplification to analyze genetic diversity and population structure of the crocodile populations [11,22,23,24,25,26,27] (Supplementary Data S1).

### 2.3. Mitochondrial DNA D-Loop Sequencing and Data Analysis

The mtDNA D-loop sequences of DNA fragments were amplified using the primers mtCytbf2 (5′-TGCCATGTTCGCATCCATCC-3) and mt12srRNAr2 (5′-CCAGAGGCTA GGCGTCGTGG-3) [11]. We used the same methods as previous studies for PCR amplification and analyze genetic diversity of the crocodile populations [11,22] (Supplementary Data S1).

## 3. Results

### Genetic Variability of Captive Crocodile Population Based on Microsatellite Data

All captive individuals were genotyped, and 459 alleles were found across all loci, with the mean number of alleles per locus being 20.864 ± 2.351 (Table 1). All allelic frequencies in the captive population significantly deviated from what would be expected under the Hardy–Weinberg equilibrium, indicating the presence of linkage disequilibrium (Tables S3–S7). Null alleles were frequently observed for 13 loci (CpP3001, CpP501, CpP214, CpP2206, CpP3313, CpP2504, CpP203, CpP1308, CpP4004, CpP3008, CpP2904, CpP3004, CpP1409), and all markers listed were treated similarly. Siamese crocodiles from CB and NR populations exhibited negative *F* values, but Siamese crocodiles from CN and NP populations exhibited positive *F* values, similar to saltwater crocodiles from NR. The *PIC* of all captive populations ranged from 0.057 to 0.932 and *I* ranged from 0.153 to 3.031 (Table S8). The *H*_o_ values ranged from 0.059 to 1.000 (mean ± standard error (SE): 0.629 ± 0.033) and the *H*_e_ values ranged from 0.058 to 0.936 (mean ± SE: 0.718 ± 0.037) (Table 1 and Table S8). Welch’s *t*-test showed that *H*_o_ was not significantly different from *H*_e_ in Table 2. By comparing pairwise *H*_o_ values between populations, there were statistical differences between six pairs, while pairwise *H*_e_ values were different between five pairs (Table 3). The *AR* value of the population was 20.787 ± 2.332. The standard genetic diversity indices are summarized in Table 1 and Supplementary Table S8.

**Figure 1:**
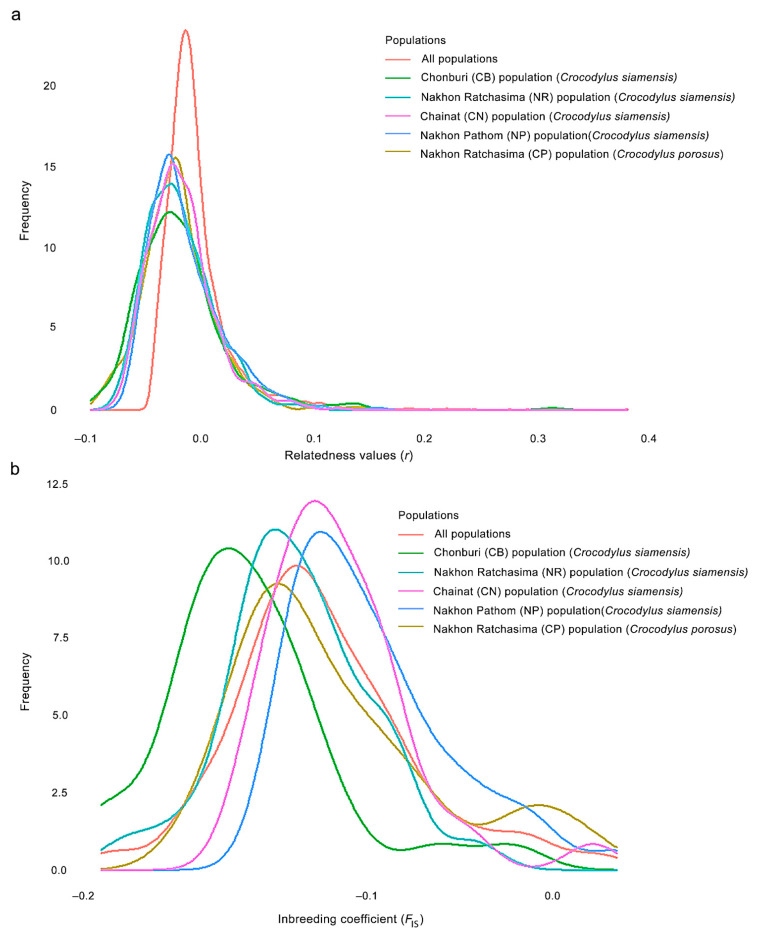
Observed distribution of (**a**) pairwise relatedness (*r*) and (**b**) inbreeding coefficients (*F*_IS_) for 136 Siamese (*Crocodylus siamensis*, Schneider, 1801) [1] and 29 saltwater crocodiles (*C. porosus*, Schneider, 1801) [13] individuals, plotted against the expected distributions.

We determined the degree of relatedness between individuals in the captive crocodile population by employing a pairwise test. The mean pairwise *r* values were calculated for a total of 13,530 combinations of crocodiles, which included all 165 sampled individuals, including Siamese and saltwater crocodiles, were −0.018 ± 0.033 (CB population = −0.018 ± 0.042, NR population = −0.019 ± 0.029, CN population = −0.017 ± 0.030, NP population = −0.019 ± 0.032 for Siamese crocodiles and NR population of saltwater crocodiles = −0.020 ± 0.032). No pairs showed *r* < −0.25. There were 13,525 pairs with −0.25 < *r* < 0.25 and 5 pairs with 0.25 > *r* (Table 2, Tables S9–S13). Distribution of *r* values for the crocodiles exhibited a left skew, indicating lower pairwise *r* values than what would be expected under a null hypothesis of unrelated individuals by chance. The distributions of pairwise r differed significantly between the CB and NP populations, and the mean pairwise *r* values were also significantly different across all populations. (Figure 1, Table S14). The mean *F*_IS_ was −0.074 ± 0.076 (Table 3), with individual values of *F*_IS_ ranging from −0.191 to 0.085 (Tables S15–S19). However, distributions of *F*_IS_ from all populations differed significantly from each other (Figure 1, Table S14). The *N*_e_ of Siamese crocodiles for individuals that contributed genetically to the CB population was 41.3 (95% CI: 32.2.8–46.5), 202.2 (95% CI: 87.7–113.3) for the NR population, 115.5 (95% CI: 69.5–88.1) for the CN population, 45.2 (95% CI: 37.1–136.1) for the NP population, and 103.7 (95% CI: 66.7–74.8) for saltwater crocodiles in the NR population (Table 4). 

**Figure 2:**
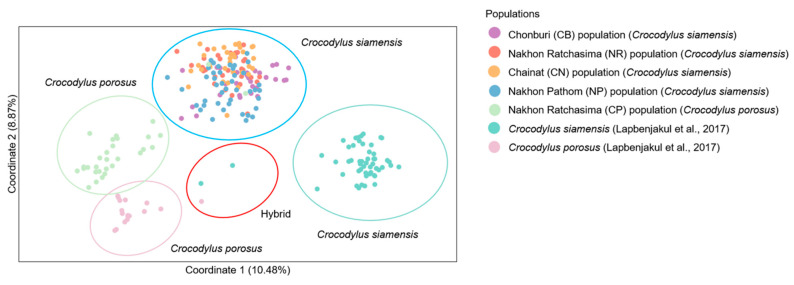
Principal component analysis of Siamese (*Crocodylus siamensis*, Schneider, 1801) [1] and saltwater crocodiles (*C. porosus*, Schneider, 1801) [13]. Table S1 provides detailed information on the sampled individuals and Lapbenjakul et al. [11].

**Figure 3:**
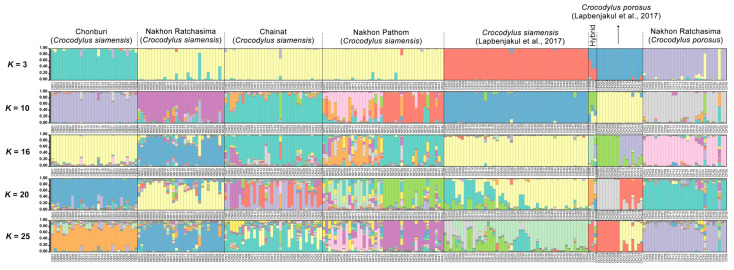
Population structure of 136 Siamese (*Crocodylus siamensis*, Schneider, 1801) [1] individuals and 29 saltwater crocodile (*C. porosus*, Schneider, 1801) [13] individuals. Each vertical bar on the *x*-axis represents an individual, while the *y*-axis represents the proportion of membership (posterior probability) in each genetic cluster. Crocodiles are superimposed on the plot, with black vertical lines indicating the boundaries. Detailed information for all crocodile individuals is presented in Table S1 and Lapbenjakul et al. [11].

Significant differences (*p* < 0.05) were observed in the estimates of *F*_ST_ between captive populations after 110 permutations. The AMOVA showed that genetic variation was 84% among individuals crocodiles within a population and 11% between populations (Table S21). Nei’s genetic distances and *R*_ST_ showed that the CN population was closer than the NP to the others (Tables S20 and S22). The distinction between the five crocodile groups and the three suspected individuals (CSI05, CSI06, and CPO09) from our previous study was supported by the first, second, and third principal components, which accounted for 10.48, 8.87, and 4.24% of the total variation, respectively, as revealed by PCoA [11] (Figure 2). Different population patterns were generated by the model-based Bayesian clustering algorithms implemented in STRUCTURE with increasing *K* values; however, the highest posterior probability with one peak (*K* = 3) based on Evanno’s Δ*K*, while the mean ln P(*K*) also revealed one peak (*K* = 16) (Figure 3 and Figure S1).

**Figure 4:**
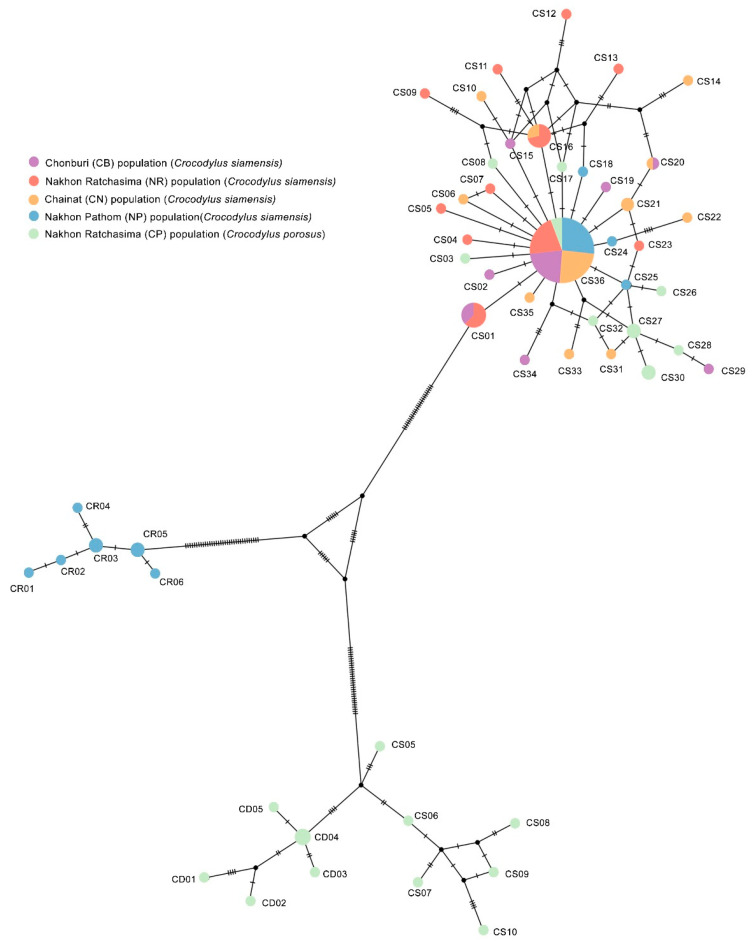
Haplotype network based on sequence data for the mitochondrial DNA D-loop region of Siamese (*Crocodylus siamensis*, Schneider, 1801) [1] and saltwater crocodiles (*Crocodylus porosus,* Schneider, 1801) [13].

The amplicon length and alignment length of the mtDNA D-loop sequences were 1500 and 1350 bp, respectively. The numbers of haplotypes were 46 and 28 for Siamese and saltwater crocodiles, respectively. Overall haplotype and nucleotide diversities were 0.846 ± 0.022 and 0.015 ± 0.003 for Siamese and 0.998 ± 0.055 and 0.069 ± 0.025 for saltwater crocodiles (Table 5). A complex haplotype network was constructed from the many detected polymorphic sites and haplotypes (Figure 4). The most common haplotype of all Siamese crocodile populations was haplotype CS36. Seven haplotypes (CS01, CS16, CS20, CS21, CS27, CS30, and CS36) were shared in the CB, NR, CN, and NP populations. Furthermore, the most common haplotype of all populations of saltwater crocodiles was haplotype CD04 and CD05. Phylogenetic analysis of a combined data set for the mtDNA D-loop sequences from both Siamese and saltwater crocodiles, together with those for 21 crocodile species obtained from the public repositories (GenBank/DDBJ/European Nucleotide Archive (ENA)), indicated that most Siamese and saltwater crocodile sequences each formed a monophyletic clade. However, NP04, NP06, NP07, NP09, NP13, NP14, NP16, and NP20, first assigned to Siamese crocodile, were grouped with Cuban crocodile (*C. rhombifer*) (GenBank accession number: NC_024513); whereas CP01, CP05, CP06, CP11, CP12, CP13, CP15, CP20, CP23, CP25, CP27, CP28, CP29, and CP30, categorized with the saltwater crocodile, were placed as a sister clade to Siamese crocodile (Figure S2). These results agreed with BLAST results of sequence identity (Table S1). To examine the genetic differentiation among the five populations, we calculated *F*_ST_, *G*_ST_, *Φ*_ST_, *D*_xy_, *D*_a_, and *N*_m_. The *F*_ST_ values ranged from −0.015 to 0.347, the *G*_ST_ values ranged from −0.008 to 0.071 and the *Φ*_ST_ values ranged from 0.013 to 0.330, The *N*_m_ values ranged from 0.947 to infinite, the *D*_xy_ values ranged from 0.002 to 0.065 and the *D*_a_ values ranged from 0.000 to 0.026 (Table 6). 

## 4. Discussion

Siamese crocodile is well-represented in captivity, with possibly over 1.5 million individuals in farms in Thailand, Cambodia, and Vietnam [31,32,33,34], and smaller numbers in farms in China and zoos in Europe and North America. In Thailand, 1400 crocodile farms with 1,319,395 Siamese and 162,449 saltwater crocodiles were operating in 2020, while 47,367 skins were sold in international trade in 2020 [35,36,37]. However, the captive population in Thailand includes an unknown number of individuals hybridized with saltwater crocodiles [11,33,38,39,40], similar to captive crocodiles in Cambodia, Lao PDR, and Vietnam [33,41,42,43]. Observations of hybrids between Siamese and saltwater crocodiles have been reported as a consequence of anthropogenic impacts [11,39,44,45]. Most anthropogenic crocodile hybrids pose a serious problem for conservation management because hybrids a possess highly similar morphology to their parental species, which might lead to introgression if they are included in a reintroduction program [11]. 

Most crocodile farms are members of the TCFA, which aims to keep purely captive-bred individuals of both species to comply with the recommendations of CITES Appendix I captive breeding operation [11,15,46]. Most Thai crocodile farms have thus pledged not to produce hybrid offspring in the interest of conservation. However, we found discrepancies in two genetic markers, microsatellite genotyping and mtDNA D-loop in several crocodile individuals (both Siamese and saltwater crocodiles), suggesting the possibility of hybrids in the population examined. Crocodiles that were clearly identified as being pure specimens of a species were designated as either “identified Siamese crocodile” or “identified saltwater crocodile”. However, if the results of the two genetic markers were not consistent, the crocodiles were designated “unidentified crocodile”. To ensure compliance between genetic tools and phenotypic variation for Siamese and saltwater crocodile identification, we compared the results of genetic diversity and structure with phenotypic observation in each crocodile. The most frequently observed means of identifying hybrid characteristics between Siamese and saltwater crocodiles are the presence of P.O. This has led to the misunderstanding of many crocodile experts and non-governmental organizations (NGOs) who have visited crocodile farms in Southeast Asia as to whether Siamese crocodiles with fewer than four post-occipital scales in a row are hybrids [16,47].

### 4.1. Are Different Numbers of Post-Occipital Scutes Due to Phenotypic Variation within Siamese Crocodiles or the Consequence of Hybridization with Saltwater Crocodiles?

**Figure 5:**
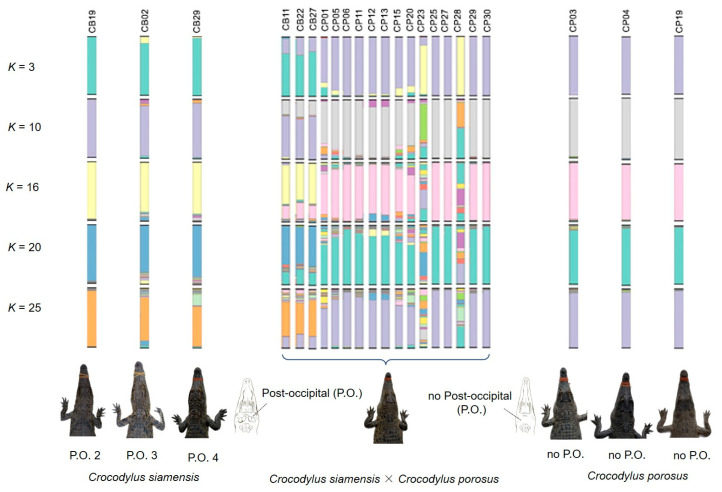
Representation of structure plot and post-occipital scutes (P.O.) for hybrids between Siamese and saltwater crocodiles (*Crocodylus siamensis* × *Crocodylus porosus*) and pure Siamese and saltwater crocodiles. Detailed information for all hybrids between Siamese and saltwater crocodile individuals is presented in Figure 3 and Figure S3.

As shown in the STRUCTURE plot and PCoA, Siamese crocodiles from CB, CN, NR, and NP, and saltwater crocodiles from NR, were likely clustered into different groups. We analyzed the clustering order and gene pool pattern from *K* = 2–25. Identified pure Siamese crocodiles, which have two P.O. scales shared the same gene pool with Siamese crocodiles having three or four scales, whereas no P.O. scales were found in identified saltwater crocodiles (Figure 3, Figure 5 and Figure S3). For *K* = 3, Siamese crocodiles (both identified and unclear individuals) from CB, CN, NR, and NP were grouped in the same gene pool, while saltwater crocodiles were part of a new group, with a small part shared with Siamese crocodiles. At higher *K* levels, saltwater crocodiles (both identified and unclear individuals) became identifiable, whereas Siamese crocodiles from different farms were separated from each other. Gene pool structuring from both species or each farm showed admixture at higher *K* levels; however, P.O. scale number variation also appeared in Siamese crocodiles with mixtures of specific gene pools of Siamese crocodiles. This suggests that Siamese crocodiles with fewer than four scales in a row are part of normal phenotypic variations at the species level. Similarly, the first version of the species identification guideline was revised after DNA analysis and proved various characteristics under the same species such as in fighting fishes [48,49]. 

This misunderstanding of widespread hybridization in Thailand has probably resulted from personal communication among experts from IUCN/SSC/Crocodile Specialist Group and other NGOs who visited farms and followed the CITES Identification Guide based on morphological characteristics in Charette [16], leading to an erroneous judgment of crocodile hybridization events in Thai crocodile farms [46,47]. Revision of Siamese crocodile identification should be reconsidered for scientific taxonomic study, which is relevant to conservation management and economic value. However, the limited number of microsatellite markers located at regular intervals cannot cover species-specific genomic regions [50]. The 22 microsatellite marker loci in this study may have caused bias due to limited population history, the timing of selection, phasing error, and false LD resolution [51,52]. Therefore, larger sample sizes with higher numbers of microsatellite loci are required to extensively investigate the evidence. Genome-wide SNP are also needed to identify signature selection between species or specific phenotypic issues such as P.O.

### 4.2. Large Gene Pool Variation Reflects Different Historical Origins in the Wild Population

Using data generated from microsatellite loci derived from Siamese and saltwater crocodiles by Lapbenjakul et al. [11] and this study, we addressed the genetic structure and gene pool pattern between the two crocodile species. As shown by PCoA, Siamese and saltwater crocodiles were clustered into different groups. However, sharing of gene pools by Siamese crocodiles between the crocodile farms was observed at different *K* levels of the STRUCTURE plot. Although Siamese crocodile individuals from this study grouped in the same gene pool at *K* = 3, and Siamese and saltwater crocodile individuals from our previous study [11] were shown to be part of a new group with saltwater crocodiles, large differences in gene pools were observed among the four populations from *K* = 5–25. In the NP population, two subpopulations of different gene pools were found, consistent with the positive fixation index value. It can be inferred from this that Siamese crocodiles in each farm had different ancestral original lineages.

Historically, Siamese crocodiles were widespread in Central Thailand. The current captive Siamese crocodile populations might reflect differences in the original sources brought into farms in Thailand. This result agreed with the genetic diversity parameters showing high values of heterozygosity, *AR*, and *N*_e_ in Siamese crocodiles from the four captive sites in this study. The pairwise *F*_ST_ value was statistically significant among the four populations, implying genetic structure differentiation between the farms. The differentiation in genetic makeup reflects the accumulation of variations in allelic frequencies, providing crucial insights into the evolutionary history, genetic drift, and selection of distinct populations [53,54]. However, we have no evidence to identify gene pool associations and geographic origin as known ecotypes, as no capture records exist for Siamese crocodile individuals. Interestingly, many Siamese crocodiles from the four captivities showed a genetic admixture of different gene pools of Siamese crocodile from *K* = 5–25. This was also observed in the saltwater crocodile population, which might have resulted from the historical genetic exchange of parental stocks between farms. Regarding mtDNA D-loop sequences, positive *N*_m_, low *F*_ST_, and sharing haplotypes of crocodiles between farms also confirmed the presence of crocodile genetic exchange in the market farms. The exchange of Siamese crocodile parental stocks has been conducted to impede inbreeding within each captive site, which may provide a negative inbreeding level. These findings collectively suggest that large captive populations of Siamese crocodiles held on farms represent a good potential source for reintroduction programs. Crocodile farms under TCFA are willing to donate Siamese crocodiles for this purpose [55]. 

### 4.3. Siamese Crocodile Identification Protocol Based on Morphology and DNA Fingerprinting

Captive crocodiles must be genetically identified at the species level before release [9,56,57]. However, hybridization between Siamese and saltwater crocodiles is widespread among some captive populations in Southeast Asia [33,34,39,58]. Differentiating hybrids from parental species based on phenotype alone is very challenging; thus, genetic screening is necessary to confirm species identity [11,15,40,58]. To ensure the success of reintroduction programs, we must first address the complex hybrid issue. Cluster analysis using STRUCTURE can now determine the degree of hybridization and gene pool pattern by aggregating individuals into a single cluster relative to additional highly differentiated populations/species [11,59,60]. Our previous study indicated that three individuals (CPO09, CSI05, and CSI06) may have been the result of interspecific hybridization between Siamese and saltwater crocodiles [11]. In this study, after we added more Siamese and saltwater crocodiles to the library analysis, the three crocodile individuals were still identified as hybrids. However, high levels of genetic admixture were observed in many crocodile individuals, and this might result in misleading conclusions about the genetic admixture of gene pools under the STRUCTURE plot with the probability of identifying the state of hybrids alone. According to genetic diversity parameters and the STRUCTURE plot, great genetic diversity and large gene pools of both species likely remain in the population, while both species are very closely related lineages [15]. The two species may share some alleles of microsatellite repeats at the same genomic locus, which is often observed in many closely related species in vertebrates [61,62]. We, therefore, proposed criteria to screen hybrid crocodiles between Siamese and saltwater crocodiles as follows: (*i*) Consideration of genetic admixture at different *K* levels to examine the trend of clustering, separation of allelic signals and the majority of allelic pattern, although the best *K* level might be predicted from different algorithms; (*ii*) sharing a gene pool between the two species might be possible, but should not have more than a posterior probability of 0.05 at the *K* level, which shows the trend of separation between the two species; (*iii*) clustering by PCoA should be considered together with the STRUCTURE plot to test the group of crocodile specimens; (*iv*) determination of maternal lineage by mtDNA D-loop sequences may be added to confirm; and (*v*) external morphological observation with updated phenotypic variations in the P.O. should be scored together with genetic screening.

**Figure 6:**
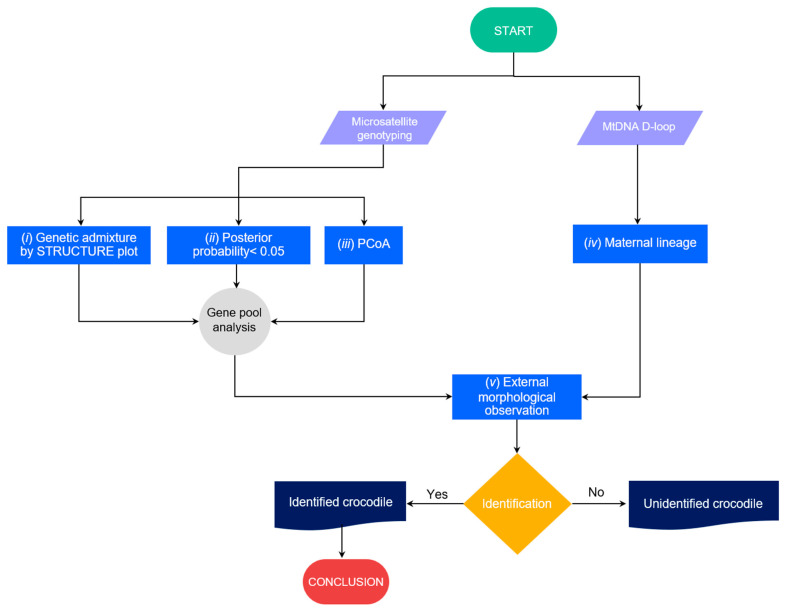
Schematic representation of criteria to screen hybrid crocodiles between Siamese and saltwater crocodiles to prove the hybridization level status in each Siamese crocodile individual before they can be used in the reintroduction program.

These five steps would provide evidence that can prove the hybridization status of each Siamese crocodile individual under reasonable time before they are used in the reintroduction program (Figure 6). Crocodiles that pass the five tests of characteristics would be key sources for release to the wild. However, if the crocodile fails on some aspects with unclear determination, the individual should undergo more experimental tests such as karyotyping. Siamese and saltwater crocodiles have different chromosome numbers, whereas the F_1_ hybrid or backcross shows diverge chromosome constitution from the parental species [63,64]. However, karyotyping is time-consuming, expensive, and may not be practical to prove species purity for large numbers of individuals, whereas multiple types and generations of hybrid (both F_2_, F_3_ or backcross) might escape detection of chromosome number. More research utilizing genome-wide scans with single nucleotide polymorphisms (SNPs) is necessary to enhance our comprehension of selection signatures in diverse populations and species. However, genome-wide SNP analysis might not be reliable for multiple processes with small crocodile numbers in each reintroduction program. From this state, we found suspected hybrids with CB11 (P.O. 4), CB22 (P.O. 3), CB27 (P.O. 4), CP01 (P.O. 0), CP05 (P.O. 4), CP06 (P.O. 4), CP11 (P.O. 4), CP12 (P.O. 4), CP13 (P.O. 4), CP15 (P.O. 2), CP20 (P.O. 0), CP23 (P.O. 4), CP25 (P.O. 2), CP27 (P.O. 4), CP28 (P.O. 4), CP29 (P.O. 0), CP30 (P.O. 0), CSI05 (unidentified P.O.), CSI06 (unidentified P.O.), and CPO09 (unidentified P.O.) (Figure 5), which should be tested by karyotyping before release.

The Thai government and TCFA have never promoted hybridization in crocodile farms under Appendix I captive breeding operation [46]; however, the incidence of contamination by hybrids was observed to be 5–10% here and in our previous study [11]. Hybrid contamination may result from a long history of crocodile trade from 30 years ago, which had no concrete plan of protection by the Thai Government. Hybridization between Siamese and Cuban crocodiles (*C. rhombifer*) as a result of human-mediated movement has also been observed in Southeast Asia [11,39,44,45]. Current results suggest that NP04, NP06, NP07, NP09, NP13, NP14, NP16, and NP20 are hybrid crocodiles derived from the Cuban crocodile lineage. We also found signs of a unique gene pool from unidentified crocodiles (suspected hybrid with Cuban crocodiles). However, we could not identify genetic admixture and introgression of Cuban crocodiles using microsatellite genotyping with our library, as there were no pure Cuban crocodiles in our experimental genetic stock [11] or in this study. Mitochondrial DNA analysis could allow us to infer the maternal lineage of Cuban crocodiles by comparing the DNA sequences with nucleotide data repositories such as GenBank, but this might not be enough for the cut-off determination of crocodile species. Thus, collaboration with crocodile research groups, governments, and NGOs such as the Crocodile Specialist Group (CSG) will be necessary for documenting and monitoring the introgression of several crocodile species in Southeast Asia.

Identifying purebred individuals from captive populations is a significant challenge for restocking and reintroduction efforts. The CSG has proposed various recommendations to support the conservation of Siamese crocodiles, including legislative and regulatory measures, compliance with CITES obligations, appropriate management of captive populations, conducting surveys and conservation initiatives, controlling illegal trade, promoting regional conservation initiatives, and exploring restocking options. These recommendations are aimed at supporting the current efforts of Thai national agencies to conserve the species, including the Department of Fisheries under the Ministry of Agriculture and Cooperatives and the Department of National Parks, Wildlife and Plant Conservation (DNP) under the Ministry of Natural Resources and Environment. To restore, protect, and create habitats for the Siamese crocodile, initiatives for public–private partnerships and sustainable financing will be launched. Prioritizing the management of key threats and conducting large-scale assessments of Siamese crocodile conservation status will be crucial for addressing challenges related to populations, protected areas, and other conservation initiatives.

## 5. Conclusions

These results indicate that a P.O. variation of 2–4 is within the species-level variation of Siamese crocodiles. In short, this is a phenotypic variation and not the result of hybridization with saltwater crocodiles. This baseline information on the association between genetic status and phenotypic variation of Siamese crocodiles in captive populations in Thailand is important for future conservation. Large differences in gene pools were observed in Siamese crocodiles, suggesting different historical origins of Siamese crocodiles in the wild population before massive capture and collection. Recently, a call was made to redefine the role of admixture in species conservation. It emphasized that crocodiles that have undergone gene flow and introgression during their evolutionary history or have been impacted by anthropogenic issues require protection measures. Ultimately, hybridization presents a management problem for Siamese crocodiles and complicates species identification based on morphology alone [65]. Adequate protocols to identify introgression and hybridization are urgently needed. Here, the genetic approach we followed proved that combining information from genetic and phenotypic approaches yielded more robust results. Accurate data on captive populations is critical for ensuring the long-term survival of the species through reintroduction programs and in situ/ex situ management, which helps to maintain sustainable genetic diversity.

## Figures and Tables

**Table 1:** Genetic diversity of 136 Siamese (*Crocodylus siamensis*, Schneider, 1801) [1] and 29 saltwater crocodiles (*C. porosus*, Schneider, 1801) [13] based on 22 microsatellite loci. Table S1 provides detailed information on the sampled individuals.

Population		N	*N*_a_	*AR*	*N*_ea_	*I*	*H*_o_	*H*_e_	*PIC*	*F*
CB ^1^	Mean	30	7.409	7.409	3.847	1.329	0.653	0.612	0.570	−0.092
	S.E.	0	0.993	0.993	0.713	0.140	0.044	0.044	0.214	0.045
NR ^2^	Mean	30	8.727	8.727	3.847	1.382	0.602	0.609	0.572	−0.031
	S.E.	0	1.405	1.405	0.728	0.149	0.043	0.043	0.213	0.066
CN ^3^	Mean	34	9.500	9.500	4.603	1.396	0.545	0.582	0.553	0.033
	S.E.	0	1.364	1.364	1.000	0.180	0.057	0.058	0.268	0.055
NP ^4^	Mean	42	11.182	11.182	4.880	1.658	0.642	0.693	0.658	0.057
	S.E.	0	1.307	1.307	0.730	0.147	0.044	0.040	0.204	0.068
CP ^5^	Mean	29	9.818	9.798	4.951	1.716	0.696	0.733	0.705	0.039
	S.E.	0	1.169	1.150	0.530	0.125	0.041	0.036	0.173	0.046
All Population	Mean	165	20.864	20.787	6.745	1.921	0.629	0.718	0.695	0.110
	S.E.	0	2.351	2.332	1.604	0.164	0.033	0.037	0.180	0.038

Sample size (N); number of alleles (*N*_a_); allelic richness (*AR*); number of effective alleles (*N*_ea_); Shannon’s information index (*I*); observed heterozygosity (*H*_o_); expected heterozygosity (*H*_e_); polymorphic information content (*PIC*); fixation index (*F*). ^1^ CB = Chonburi (*Crocodylus siamensis*). ^2^ NR = Nakhon Ratchasima (*Crocodylus siamensis*). ^3^ CN = Chainat (*Crocodylus siamensis*). ^4^ NP = Nakhon Pathom (*Crocodylus siamensis*). ^5^ CP = Nakhon Ratchasima (*Crocodylus porosus*).

**Table 2:** Welch’s *t*-test of observed heterozygosity (*H*_o_) and expected heterozygosity (*H*_e_) of Siamese (*Crocodylus siamensis*, Schneider, 1801) [1] and saltwater crocodiles (*Crocodylus porosus*, Schneider, 1801) [13] based on 22 microsatellite loci.

Population	*H*_o_	*H*_e_	df	*t*-Test	*p*-Value
CB ^1^	0.641 ± 0.044	0.612 ± 0.044	0.041	0.466	0.643
NR ^2^	0.602 ± 0.043	0.609 ± 0.043	−0.037	−0.115	0.909
CN ^3^	0.545 ± 0.057	0.582 ± 0.058	0.007	−0.455	0.651
NP ^4^	0.642 ± 0.044	0.693 ± 0.040	−0.051	−0.858	0.394
CP ^5^	0.696 ± 0.041	0.733 ± 0.036	−0.037	−0.678	0.501

^1^ CB = Chonburi (*Crocodylus siamensis*). ^2^ NR = Nakhon Ratchasima (*Crocodylus siamensis*). ^3^ CN = Chainat (*Crocodylus siamensis*). ^4^ NP = Nakhon Pathom (*Crocodylus siamensis*). ^5^ CP = Nakhon Ratchasima (*Crocodylus porosus*).

**Table 3:** Comparison of genetic diversity parameters between Siamese (*Crocodylus siamensis*, Schneider, 1801) [1] and saltwater crocodiles (*Crocodylus porosus*, Schneider, 1801) [13] based on 22 microsatellite loci. Table S1 provides detailed information on the sampled individuals.

	Population 1	Population 2	df	SE	*t*-Test	*p*-Value
Heterozygosity (*H*_o_)	CB ^1^	NR ^2^	0.051	0.011	4.540	<0.05
	CB	CN ^3^	0.108	0.013	8.536	<0.05
	CB	NP ^4^	0.011	0.099	0.111	0.912
	CB	CP ^5^	−0.051	0.010	−4.914	<0.05
	NR	CN	0.057	0.013	4.546	<0.05
	NR	NP	−0.040	0.099	−0.403	0.689
	NR	CP	−0.102	0.010	−9.962	<0.05
	CN	NP	−0.097	0.100	−0.974	0.335
	CN	CP	−0.159	0.012	−13.498	<0.05
	NP	CP	−0.062	0.099	−0.624	0.536
Heterozygosity (*H_e_*)	CB	NR	0.003	0.011	0.267	0.790
	CB	CN	0.030	0.013	2.346	<0.05
	CB	NP	−0.081	0.107	−0.755	0.454
	CB	CP	−0.135	0.010	−12.862	<0.05
	NR	CN	0.027	0.013	2.131	<0.05
	NR	NP	−0.084	0.107	−0.783	0.438
	NR	CP	−0.138	0.010	−13.324	<0.05
	CN	NP	−0.111	0.107	−1.034	0.307
	CN	CP	−0.165	0.012	−13.723	<0.05
	NP	CP	−0.054	0.107	−0.504	0.617

^1^ CB = Chonburi (*Crocodylus siamensis*). ^2^ NR = Nakhon Ratchasima (*Crocodylus siamensis*). ^3^ CN = Chainat (*Crocodylus siamensis*). ^4^ NP = Nakhon Pathom (*Crocodylus siamensis*). ^5^ CP = Nakhon Ratchasima (*Crocodylus porosus*).

**Table 4:** Inbreeding coefficients, relatedness, effective population size, and ratio of effective population size and census population (*N*_e_*/N*) of 136 Siamese (*Crocodylus siamensis*, Schneider, 1801) [1] and 29 saltwater crocodiles (*C. porosus*, Schneider, 1801) [13].

Population	*N*	*F*_IS_	Relatedness (*r*)	Estimated *N*_e_	95% CIs for *N*_e_	*N*_e_ */* *N*
CB ^1^	30	−0.113 ± 0.201	−0.018 ± 0.042	41.300	32.200–46.500	1.377
NR ^2^	30	−0.086 ± 0.039	−0.019 ± 0.029	202.200	87.700–113.300	6.740
CN ^3^	34	−0.063 ± 0.039	−0.017 ± 0.030	115.500	69.500–88.100	3.397
NP ^4^	42	−0.045 ± 0.046	−0.019 ± 0.032	45.200	37.100–136.100	1.076
CP ^5^	29	−0.065 ± 0.055	−0.020 ± 0.032	103.700	66.700–74.800	3.576

Estimates were calculated using GenAlEx version 6.5 [28]. NeEstimator version 2.1 [29], and COANCESTRY version 1.0.1.9 [30]. Detailed information for all elephant individuals is presented in Table S1. Sample size (*N*); inbreeding coefficient (*F*_IS_); effective population size (*N*_e_). ^1^ CB = Chonburi (*Crocodylus siamensis*). ^2^ NR = Nakhon Ratchasima (*Crocodylus siamensis*). ^3^ CN = Chainat (*Crocodylus siamensis*). ^4^ NP = Nakhon Pathom (*Crocodylus siamensis*). ^5^ CP = Nakhon Ratchasima (*Crocodylus porosus*).

**Table 5:** Mitochondrial DNA D-loop sequence diversity of Siamese (*Crocodylus siamensis*, Schneider, 1801) [1] and saltwater crocodiles (*Crocodylus porosus*, Schneider, 1801) [13].

Population	*N*	Number of Haplotypes (H)	Theta (per Site) from *S*	Average Number of Nucleotide Differences (*k*)	Overall Haplotype	Nucleotide Diversities (*π*)
CB ^1^	30	20	0.016	6.786	0.959 ± 0.022	0.013 ± 0.006
NR ^2^	30	18	0.007	3.471	0.940 ± 0.027	0.015 ± 0.008
CN ^3^	34	20	0.010	4.783	0.934 ± 0.027	0.022 ± 0.011
NP ^4^	42	24	0.036	40.539	0.922 ± 0.032	0.072 ± 0.035
CP ^5^	29	28	0.052	56.924	0.998 ± 0.010	0.069 ± 0.025
All populations	165	54	0.043	18.024	0.725 ± 0.039	0.040 ± 0.020

^1^ CB = Chonburi (*Crocodylus siamensis*). ^2^ NR = Nakhon Ratchasima (*Crocodylus siamensis*). ^3^ CN = Chainat (*Crocodylus siamensis*). ^4^ NP = Nakhon Pathom (*Crocodylus siamensis*). ^5^ CP = Nakhon Ratchasima (*Crocodylus porosus*).

**Table 6:** Genetic differentiation between the three populations of Siamese (*Crocodylus siamensis*, Schneider, 1801) [1] and saltwater crocodiles (*Crocodylus porosus*, Schneider, 1801) [13] for the mitochondrial DNA D-loop sequence. Genetic differentiation coefficient (*G*_ST_), Wright’s *F*-statistic for the subpopulations within the total population (*F*_ST_), *Φ*_ST_, gene flow (*N*_m_) from the sequence data and the haplotype data, the average number of nucleotide substitutions per site between populations (*D*_xy_), and the net nucleotide substitutions per site between populations (*D*_a_).

Population 1	Population 2	*G*_ST_	*Φ*_ST_	*F*_ST_	*D*_xy_	*D*_a_	*N*_m_
CB ^1^	NR ^2^	−0.008	0.013	0.045 *	0.002	0.000	10.620
CB	CN ^3^	0.000	0.017	0.027 ^ns^	0.002	0.000	18.224
CB	NP ^4^	0.005	0.083	0.127 **	0.021	0.003	3.432
CB	CP ^5^	0.071	0.320	0.347 **	0.055	0.025	0.941
NR	CN	0.006	0.025	−0.015 ^ns^	0.003	0.000	Infinite
NR	NP	0.009	0.081	0.094 **	0.021	0.003	4.840
NR	CP	0.066	0.318	0.345 **	0.055	0.025	0.949
CN	NP	0.000	0.087	0.090 *	0.021	0.003	5.064
CN	CP	0.067	0.330	0.345 **	0.055	0.026	0.950
NP	CP	0.050	0.198	0.261 *	0.065	0.020	1.418

ns = not significant, * *p* < 0.05, ** *p* < 0.01. ^1^ CB = Chonburi (*Crocodylus siamensis*). ^2^ NR = Nakhon Ratchasima (*Crocodylus siamensis*). ^3^ CN = Chainat (*Crocodylus siamensis*). ^4^ NP = Nakhon Pathom (*Crocodylus siamensis*). ^5^ CP = Nakhon Ratchasima (*Crocodylus porosus*).

## Data Availability

All sequences were deposited in the DNA Data Bank of Japan (DDBJ). The online version contains Supplementary Materials available at https://datadryad.org/stash/share/s4zREYQ1AUUXTsaIpk0r1HSdkYljvu8yvJOVR143K7Y (accessed on 18 February 2018).

## Supplementary Materials

The following supporting information can be downloaded at: https://www.mdpi.com/article/10.3390/biology12040535/s1, Figure S1. Population structure of 136 Siamese crocodiles (*Crocodylus siamensis*, Schneider, 1801) [1] and 29 saltwater crocodiles (*C. porosus*, Schneider, 1801) [13], (a) Plot of Evanno’s Δ*K* and (b) plot of ln P(*K*); Figure S2. Phylogenetic relationships among mitochondrial DNA D-loop region sequences were inferred using Bayesian inference analysis. Support values at each node denote the Bayesian posterior probability. Table S1 provides detailed information on the sampled individuals; Figure S3. Representation post-occipital scutes (P.O.) for hybrids between Siamese and saltwater crocodiles (*Crocodylus siamensis* × *Crocodylus porosus*). (a) CB11 (b) CB22 (c) CB27 (d) CP01 (e) CP05 (f) CP06 (g) CP11 (h) CP12 (i) CP13 (j) CP15 (k) CP20 (l) CP23 (m) CP25 (n) CP27 (o) CP28 (p) CP29 (q) CP30; Table S1. Specimen populations of Siamese (*Crocodylus siamensis*, Schneider, 1801) [1] and saltwater crocodiles (*C. porosus*, Schneider, 1801) [13] All sequences were deposited in the DNA Data Bank of Japan (DDBJ) and BLASTn (http://blast.ncbi.nlm.nih.gov/Blast.cgi, accessed on 8 February 2023) of sequence identity; Table S2. Microsatellite primers and sequences; Table S3. Pairwise differentiation of linkage disequilibrium of Siamese crocodiles (*Crocodylus siamensis*, Schneider, 1801) [1] at Chonburi (CB) based on 22 microsatellite loci. Numbers indicate *p*-values with 110 permutations; Table S4. Pairwise differentiation of linkage disequilibrium of Siamese crocodiles (*Crocodylus siamensis*, Schneider, 1801) [1] at Nakhon Ratchasima (NR) based on 22 microsatellite loci. Numbers indicate *p*-values with 110 permutations; Table S5. Pairwise differentiation of linkage disequilibrium of Siamese crocodiles (*Crocodylus siamensis*, Schneider, 1801) [1] at Chainat (CN) based on 22 microsatellite loci. Numbers indicate *p*-values with 110 permutations; Table S6. Pairwise differentiation of linkage disequilibrium of Siamese crocodiles (*Crocodylus siamensis*, Schneider, 1801) [1] at Nakhon Pathom (NP) based on 22 microsatellite loci. Numbers indicate *p*-values with 110 permutations; Table S7. Pairwise differentiation of linkage disequilibrium of saltwater crocodiles (*Crocodylus porosus*, Schneider, 1801) [13] at Nakhon Ratchasima (CP) based on 22 microsatellite loci. Numbers indicate *p*-values with 110 permutations; Table S8. Genetic diversity of 136 Siamese crocodiles (*Crocodylus siamensis*, Schneider, 1801) [1] and 29 saltwater crocodiles (*C. porosus*, Schneider, 1801) [13] based on 22 microsatellite loci. Table S1 provides detailed information on the sampled individuals; Table S9. Pairwise genetic relatedness (*r*) for all 30 Siamese crocodiles *(Crocodylus siamensis*, Schneider, 1801) [1] in Chonburi (CB) Table S1 provides detailed information on the sampled individuals; Table S10. Pairwise genetic relatedness (*r*) for all 30 Siamese crocodiles *(Crocodylus siamensis*, Schneider, 1801) [1] in Nakhon Ratchasima (NR) Table S1 provides detailed information on the sampled individuals; Table S11. Pairwise genetic relatedness (*r*) for all 34 Siamese crocodiles *(Crocodylus siamensis*, Schneider, 1801) [1] in Chainat (CN). Table S1 provides detailed information on the sampled individuals; Table S12. Pairwise genetic relatedness (*r*) for all 42 Siamese crocodiles *(Crocodylus siamensis*, Schneider, 1801) [1] in Nakhon Pathom (NP). Table S1 provides detailed information on the sampled individuals; Table S13. Pairwise genetic relatedness (*r*) for all 29 saltwater crocodiles *(Crocodylus porosus*, Schneider, 1801) [3] in Nakhon Ratchasima (CP). Table S1 provides detailed information on the sampled individuals; Table S14. Distributions of *r* values and *F*_IS_ values for Siamese (*Crocodylus siamensis*, Schneider, 1801) [1] and saltwater crocodiles (*Crocodylus porosus*, Schneider, 1801) [1]; Table S15. Pairwise inbreeding coefficients (*F*_IS_) for all 30 Siamese crocodiles (*Crocodylus siamensis*, Schneider, 1801) [1] in Chonburi (CB). Detailed information on all Siamese crocodile individuals is presented in Table S1; Table S16. Pairwise inbreeding coefficients (*F*_IS_) for all 30 Siamese crocodiles (*Crocodylus siamensis*, Schneider, 1801) [1] in Nakhon Ratchasima (NR). Detailed information on all Siamese crocodile individuals is presented in Table S1; Table S17. Pairwise inbreeding coefficients (*F*_IS_) for all 34 Siamese crocodiles (*Crocodylus siamensis*, Schneider, 1801) [1] in Chainat (CN). Detailed information on all Siamese crocodile individuals is presented in Table S1; Table S18. Pairwise inbreeding coefficients (*F*_IS_) for all 42 Siamese crocodiles (*Crocodylus siamensis*, Schneider, 1801) [1] in Nakhon Pathom (NP). Detailed information on all Siamese crocodile individuals is presented in Table S1; Table S19. Pairwise inbreeding coefficients (*F*_IS_) for all 29 saltwater crocodiles (*Crocodylus porosus*, Schneider, 1801) [1] in Nakhon Ratchasima (CP). Detailed information on all saltwater crocodile individuals is presented in Table S1; Table S20. Pairwise genetic differentiation (*F_ST_*), pairwise *F*_ST_^ENA^ values with ENA correction for null alleles and *R*_ST_ values using FSTAT version 2.9.3 [66] and of Siamese (*Crocodylus siamensis*, Schneider, 1801) [1] and saltwater crocodiles (*Crocodylus porosus*, Schneider, 1801) [13] between captive-bred individuals based on 22 microsatellite loci. The numbers indicate *p* values, with 110 permutations. Detailed information on all Siamese and saltwater crocodile individuals is presented in Table S1; Table S21. Analysis of molecular variance (AMOVA) results for Siamese crocodile (*Crocodylus siamensis*, Schneider, 1801) [1] and crocodiles (*Crocodylus porosus*, Schneider, 1801) [1] based on 22 microsatellite loci using Arlequin version 3.5.2.2 [67]. Detailed information on all Siamese and saltwater crocodiles is presented in Table S1; Table S22. Pairwise population Nei’s genetic distance (*D*) values using GenAlEx version 6.5 [28] of 166 Siamese (*Crocodylus siamensis*, Schneider, 1801) [1] and saltwater crocodiles (*Crocodylus porosus*, Schneider, 1801) [13] based on 22 microsatellite loci. Detailed information on all Siamese and saltwater crocodiles is presented in Table S1; Supplementary Data S1 Materials and Methods [66,67,68,69,70,71,72,73,74,75,76,77,78,79,80,81,82,83,84,85].
