# Supplementary material for: Should the Identification Guidelines for Siamese Crocodiles Be Revised? Differing Post-Occipital Scute Scale Numbers Show Phenotypic Variation Does Not Result from Hybridization with Saltwater Crocodiles
Source: Biology (Basel). 2023 Mar 31;12(4):535. doi: 10.3390/biology12040535 (PMC10136156; doi:10.3390/biology12040535)
Supplement: Supplementary file 1 [file biology-12-00535-s001.zip › Supplementary Material for Biology_V1.pdf]

Supplementary Material

Supplementary Data

1. Supplementary Data S1

1.1 Materials and Methods

DNA extraction

All crocodiles were released immediately after specimen collection. Total genomic DNA was extracted from the scale following the standard salting-out protocol as described previously [1] and used as the template for microsatellite genotyping. DNA quality and quantity were determined by electrophoresis on 1% agarose gel and a NanoDrop™ 2000 Spectrophotometer (Thermo Fisher Scientific, Wilmington, DE, USA).

Microsatellite genotyping

Twenty-two microsatellite primer sets, developed originally from saltwater crocodiles (Table S2) [2,3], were used for the genotyping of all crocodile individuals. Cross-species amplification of microsatellite primers is frequently performed in conservation-focused genetic and biodiversity research [4,5–10]. The 5′-end of the forward primer from each primer set was labeled with a fluorescent dye (6-FAM or ROX; Macrogen Inc., Seoul, Korea). PCR amplification was performed using 15 μL of 1× ThermoPol buffer containing 1.5 mM $MgCl_2$, 0.2 mM dNTPs, 5.0 μM primers, 0.5 U Taq polymerase (Apsalagen Co., Ltd., Bangkok, Thailand), and 25 ng genomic DNA. The PCR protocol was as follows: initial denaturation at 94°C for 3 min, followed by 35 cycles of 94°C for 30 s, 55°C for 30 s, and 72°C for 30 s, with a final extension at 72°C for 10 min (Table S2). The PCR products were detected by electrophoresis in 1% agarose gel. To decrease the influence of false alleles, PCR amplification was performed at least three times for each sample. Fluorescent DNA fragment length analysis was subsequently performed using an ABI 3730XL Automatic Sequencer (Applied Biosystems, Foster City, CA, USA) at the DNA sequencing service of Macrogen Inc. Allelic size was determined using Peak Scanner version 1.0 (Applied Biosystems).

Microsatellite data analysis

We followed the same approaches as those used in our previous studies on other animals [4,5–10]. Allelic frequency, number of alleles (A), effective number of alleles (N_a), observed heterozygosity (H_o), expected heterozygosity (H_e) and Wright's F-statistic for subpopulations within the total population (F_{ST}) were calculated using Arlequin version 3.5.2.2 [11]. Given that the population was small, deviations from the Hardy–Weinberg equilibrium were evaluated at each locus by the Markov chain Monte Carlo (MCMC) approximation of Fisher's exact test using the "genepop" function implemented in the "stats" package of R version 4.1.2 [12–14]. Welch's t-test, which does not assume equal variance between samples, was used to test for significant differences between H_o and H_e using the "t.test" function in the "stats" package of R version 4.1.2 [15,16]. To test for equal variances between the H_o and H_e of all captivities, Bartlett's test of homogeneity of variances was first conducted using the "bartlett.test" function in the "stats" package of R version 4.1.2 [14]. Allelic richness (AR) was calculated using FSTAT version 2.9.4 [17] and MicroChecker version 2.2.3 was used to identify null allelic markers [18]. Polymorphic information content (PIC) was estimated using the Excel Microsatellite Toolkit and calculated for each locus. Shannon's information index (I) and fixation index (F) were calculated for each locus of the population using GenAlEx version 6.5 [19,20]. Effective population size (N_e) was estimated as the number of breeding individuals that contributed to the population using the linkage disequilibrium method in NeEstimator version 2.01 [21].

To consider the possibility of sibling or parent-offspring pairs in the captive population, we determined whether crocodiles were more related than random unrelated individuals. Relatedness values (*r*) were calculated for all pairs (comprising female-female, male-male, and male-female pairs), and mean pairwise *r* values based on allelic frequencies in the population were calculated using GenAlEx version 6.5 [20]. Distributions of pairwise *r* values between all pairs from the sampled captivities were compared using a bootstrap version of the Kolmogorov-Smirnov test to provide relationships [21], using the "ks.test" function in the "stats" package of R version 4.1.2 [14]. The same approach was adopted for the comparison of inbreeding coefficients (F_{IS}) among captive locations for individual and overall F_{IS} at 95% confidence intervals (CIs) were calculated using the LynchRt estimator [22] as implemented in the program COANCESTRY [23]. The *r* values and F_{IS} were examined under the assumption that the averages did not differ significantly from random assortments of unrelated individuals. Pairwise genetic distances among populations were calculated based on the infinite allele model (IAM) using F_{ST} in Arlequin version 3.5.2.2 with corrected *p* values, and the stepwise mutation model (SMM) using R_{ST} in FSTAT version 2.9.3 [16]. To consider possible influences of null alleles on genetic differentiation estimates, the Free^{NA} program (Chapuis and Estoup, 2007) [24] was run, thereby providing pairwise F_{ST} ^{ENA} values with ENA correction for null alleles. To elucidate the group structure, an analysis of molecular variance (AMOVA) was performed using GenAlEx version 6.5. Unlike F_{ST}, this algorithm identifies subgroup hierarchical structure and does not require an a priori assumption of the Hardy–Weinberg equilibrium. Nei's genetic distances between groups were then examined using GenAlEx version 6.5 [25].

Principal coordinate analysis (PCoA) was performed to assess the overall relationship across individuals in the captive population using GenAlEx version 6.5. We performed PCoA analysis with crocodiles sampled in this study and our previous study [4] to determine the population cluster between Siamese and saltwater crocodiles. PCoA defines synthetic variables where the genetic variation is maximized between clusters of individuals (*K*) and minimized within clusters. The model-based clustering method was implemented in STRUCTURE version 2.3.4 and ran in parallel using Structure_threader to determine population structure [27]. Run length was set to 100,000 MCMC replicates after a burn-in period of 100,000 generations, using correlated allelic frequencies under a straight admixture model. The number of clusters (*K*) varied from 1 to 25, with 15 replicates for each value of *K*. The most probable number of bunches was determined by plotting the log-likelihood of the information (ln Pr ($X|K$)) [26] over the scope of tested *K* esteems before choosing the *K* esteem value where ln Pr ($X|K$) settled. The Δ*K* strategy was also applied using Structure Harvester [27].

Mitochondrial DNA D-loop sequencing

The mtDNA D-loop sequences of DNA fragments were amplified using the primers mtCytbf2 (5′ -TGCCATGTTCGCATCCATCC-3) and mt12srRNAr2 (5′ -CCAGAGGCTA GGCGTCGTGG-3) [4]. Each PCR amplification was performed using 15 μL of 1 × ThermoPol® buffer containing 1.5 mM $MgCl_2$, 0.2 mM dNTPs, 5.0 μM primers, 0.5 U Taq polymerase (Apsalagen Co., Ltd., Bangkok, Thailand) and 25 ng genomic DNA. The PCR conditions were as follows: initial denaturation at 94°C for 4 min, followed by 35 cycles of 94°C for 30 s, 63°C for 45 s, 72°C for 30 s, and a final extension at 72°C for 10 min. The PCR products were purified using the FavorPrep GEL/PCR Purification Mini Kit (Favorgen Biotech Corp., Ping-Tung, Taiwan). Nucleotide sequences of the DNA fragments were determined by the DNA sequencing service of First Base Laboratories Sdn Bhd (Seri Kembangan, Selangor, Malaysia). BLASTn (http://blast.ncbi.nlm.nih.gov/Blast.cgi) was used to search nucleotide sequences in the National Center for Biotechnology Information database to confirm the identity of the amplified DNA fragments

Mitochondrial DNA D-loop data analysis

Multiple alignments of the 165 partial mtDNA D-loop sequences were performed using the default parameters of Molecular Evolutionary Genetics Analysis 11 (MEGA11) software version 11 (Center for

Evolutionary Functional Genomics, The Biodesign Institute, Temple, PA, USA) [28]. All unalignable and gap-containing sites were carefully removed and trimmed from the datasets. Estimates of haplotype (*h*), and nucleotide (π) diversity, number of haplotypes (*H*), the estimator theta (*S*), overall haplotype and average number of nucleotide differences (*k*) were calculated based on the mtDNA D-loop sequences, as implemented in DnaSP version 6.12.03 [29]. The genetic differentiation coefficient (G_{ST}), Wright's *F*-statistic for subpopulations within the total population (F_{ST}), Φ_{ST} values, and gene flow (N_m) from the sequence data and haplotype data were estimated using Arlequin version 3.5.2.2 [11]. The F_{ST} and Φ_{ST} values were calculated by analyzing 1,000 permutations of haplotypes between populations [30]. The F_{ST} statistic is based only on the difference in haplotype frequencies, whereas Φ_{ST} considers the relationships between haplotypes based on molecular genetic distance [31]. The average number of nucleotide substitutions per site between populations (D_{xy}) and the net nucleotide substitutions per site between populations (D_a) were assessed using DnaSP version 6.12.03. A statistical parsimony network of the consensus sequences was constructed using the Templeton, Crandall, and Sing (TCS) algorithm implemented in PopART version 1.7 to examine haplotype grouping and population dynamics [32].

Representative sequences of crocodile mtDNA were used to construct the haplotype network and phylogenetic trees. Phylogenetic analysis was then performed using Bayesian inference with MrBayes v3.2.7a [33]. The MCMC process was used to run four chains simultaneously for one million generations, sampling every 100 generations. The Bayesian posterior probability in the sampled tree population was obtained in percentage terms.

2. Supplementary Figures and Tables
2.1 Supplementary Figures

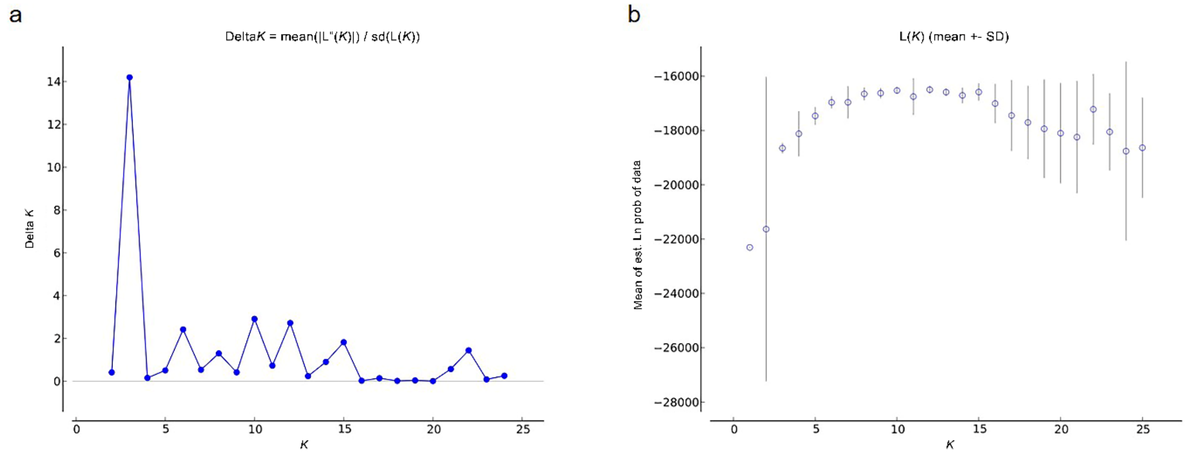

Figure S1. Population structure of 136 Siamese crocodiles (*Crocodylus siamensis*, Schneider, 1801) [34] and 29 saltwater crocodiles (*C. porosus*, Schneider, 1801) [35], (a) Plot of Evanno's ΔK and (b) plot of ln P(K).

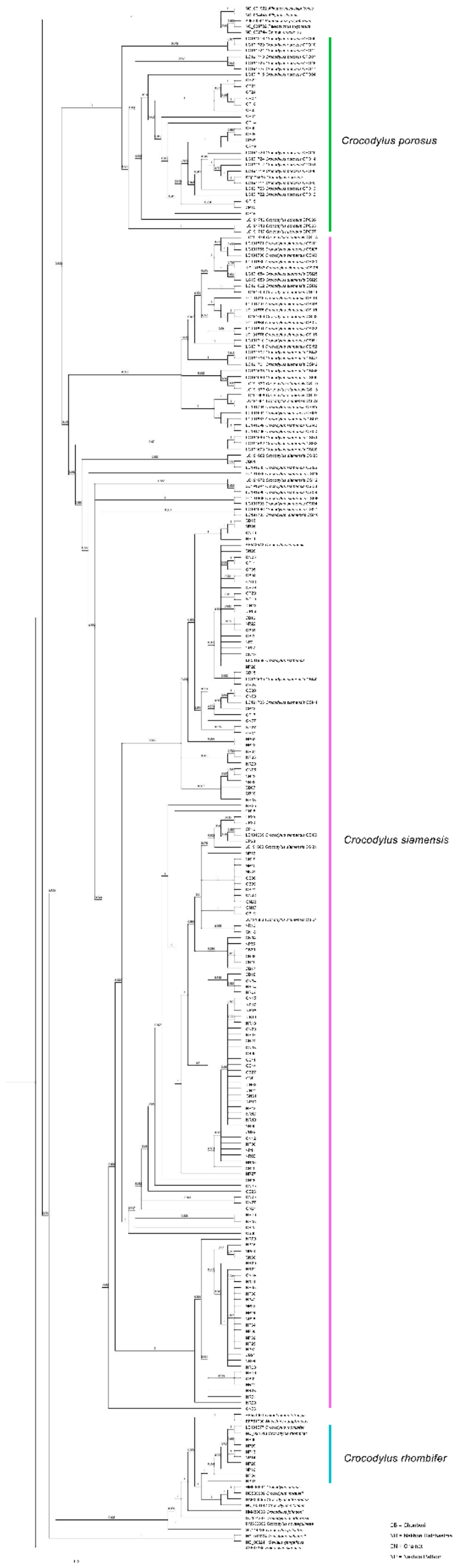

Figure S2. Phylogenetic relationships among mitochondrial DNA D-loop region sequences were inferred using Bayesian inference analysis. Support values at each node denote the Bayesian posterior probability. Table S1 provides detailed information on the sampled individuals.

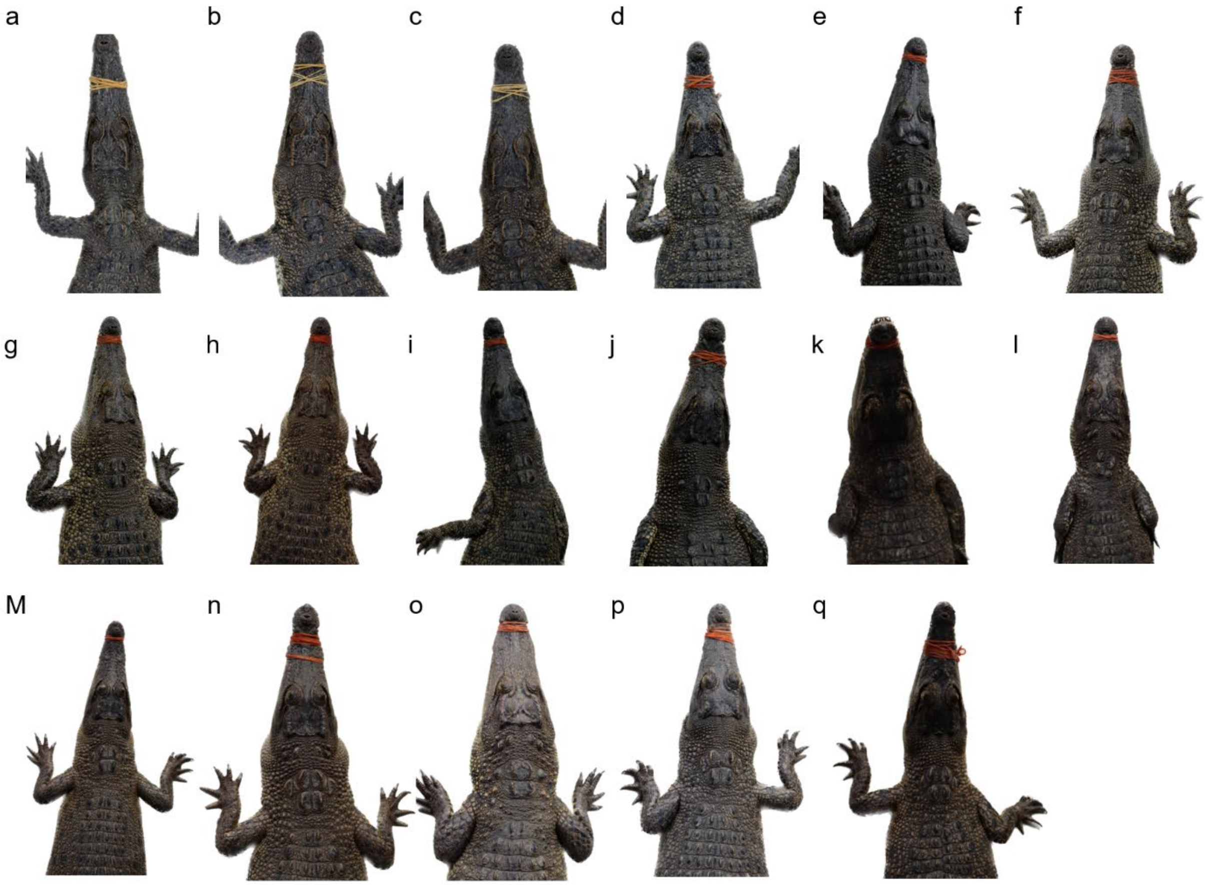

Figure S3. Representation post-occipital scutes (P.O.) for hybrids between Siamese and saltwater crocodiles (*Crocodylus siamensis* x *Crocodylus porosus*). (a) CB11 (b) CB22 (c) CB27 (d) CP01 (e) CP05 (f) CP06 (g) CP11 (h) CP12 (i) CP13 (j) CP15 (k) CP20 (l) CP23 (m) CP25 (n) CP27 (o) CP28 (p) CP29 (q) CP30.

2.2 Supplementary Tables

Table S1 Specimen populations of Siamese crocodile (*Crocodylus siamensis*, Schneider, 1801) [34] and saltwater crocodile (*C. porosus*, Schneider, 1801) [35] in this study. All sequences were deposited in the DNA Data Bank of Japan (DDBJ) and BLASTn (http://blast.ncbi.nlm.nih.gov/Blast.cgi) of sequence identity.

No.	Abbreviation/ Code	species	Locality	Mitochondrial D-loop DDBJ accession number	Percent identity with species	
					Percent identity	Species
1	CB01	*Crocodylus siamensis*	CB, Chonburi	LC754533	95.21	*C. siamensis*
2	CB02	*Crocodylus siamensis*	CB, Chonburi	LC754534	99.37	*C. siamensis*
3	CB03	*Crocodylus siamensis*	CB, Chonburi	LC754535	99.55	*C. siamensis*
4	CB04	*Crocodylus siamensis*	CB, Chonburi	LC754536	98.65	*C. siamensis*
5	CB05	*Crocodylus siamensis*	CB, Chonburi	LC754537	99.55	*C. siamensis*
6	CB06	*Crocodylus siamensis*	CB, Chonburi	LC754538	99.46	*C. siamensis*
7	CB07	*Crocodylus siamensis*	CB, Chonburi	LC754539	98.55	*C. siamensis*
8	CB08	*Crocodylus siamensis*	CB, Chonburi	LC754540	98.21	*C. siamensis*
9	CB09	*Crocodylus siamensis*	CB, Chonburi	LC754541	99.46	*C. siamensis*
10	CB10	*Crocodylus siamensis*	CB, Chonburi	LC754542	98.64	*C. siamensis*
11	CB11	*Crocodylus siamensis*	CB, Chonburi	LC754543	99.55	*C. siamensis*
12	CB12	*Crocodylus siamensis*	CB, Chonburi	LC754544	99.28	*C. siamensis*
13	CB13	*Crocodylus siamensis*	CB, Chonburi	LC754545	99.37	*C. siamensis*
14	CB14	*Crocodylus siamensis*	CB, Chonburi	LC754546	99.55	*C. siamensis*
15	CB15	*Crocodylus siamensis*	CB, Chonburi	LC754547	98.11	*C. siamensis*
16	CB16	*Crocodylus siamensis*	CB, Chonburi	LC754548	97.59	*C. siamensis*
17	CB17	*Crocodylus siamensis*	CB, Chonburi	LC754549	99.28	*C. siamensis*
18	CB18	*Crocodylus siamensis*	CB, Chonburi	LC754550	99.46	*C. siamensis*
19	CB19	*Crocodylus siamensis*	CB, Chonburi	LC754551	99.10	*C. siamensis*
20	CB20	*Crocodylus siamensis*	CB, Chonburi	LC754552	99.37	*C. siamensis*
21	CB21	*Crocodylus siamensis*	CB, Chonburi	LC754553	98.72	*C. siamensis*
22	CB22	*Crocodylus siamensis*	CB, Chonburi	LC754554	99.46	*C. siamensis*
23	CB23	*Crocodylus siamensis*	CB, Chonburi	LC754555	97.95	*C. siamensis*
24	CB24	*Crocodylus siamensis*	CB, Chonburi	LC754556	99.46	*C. siamensis*
25	CB25	*Crocodylus siamensis*	CB, Chonburi	LC754557	98.29	*C. siamensis*
26	CB26	*Crocodylus siamensis*	CB, Chonburi	LC754558	99.55	*C. siamensis*
27	CB27	*Crocodylus siamensis*	CB, Chonburi	LC754559	99.55	*C. siamensis*
28	CB28	*Crocodylus siamensis*	CB, Chonburi	LC754560	97.95	*C. siamensis*
29	CB29	*Crocodylus siamensis*	CB, Chonburi	LC754561	99.28	*C. siamensis*
30	CB30	*Crocodylus siamensis*	CB, Chonburi	LC754562	99.28	*C. siamensis*
31	NR01	*Crocodylus siamensis*	NR, Nakhon Ratchasima	LC754668	99.28	*C. siamensis*
32	NR02	*Crocodylus siamensis*	NR, Nakhon Ratchasima	LC754669	99.55	*C. siamensis*
33	NR03	*Crocodylus siamensis*	NR, Nakhon Ratchasima	LC754670	98.38	*C. siamensis*
34	NR04	*Crocodylus siamensis*	NR, Nakhon Ratchasima	LC754671	98.72	*C. siamensis*
35	NR05	*Crocodylus siamensis*	NR, Nakhon Ratchasima	LC754672	99.46	*C. siamensis*
36	NR06	*Crocodylus siamensis*	NR, Nakhon Ratchasima	LC754673	99.46	*C. siamensis*
37	NR07	*Crocodylus siamensis*	NR, Nakhon Ratchasima	LC754674	99.46	*C. siamensis*
38	NR08	*Crocodylus siamensis*	NR, Nakhon Ratchasima	LC754675	98.72	*C. siamensis*

No.	Abbreviation/ Code	species	Locality	Mitochondrial D-loop DDBJ accession number	Percent identity with species	
					Percent identity	Species
39	NR09	*Crocodylus siamensis*	NR, Nakhon Ratchasima	LC754675	98.72	*C. siamensis*
40	NR10	*Crocodylus siamensis*	NR, Nakhon Ratchasima	LC754676	98.04	*C. siamensis*
41	NR11	*Crocodylus siamensis*	NR, Nakhon Ratchasima	LC754677	98.55	*C. siamensis*
42	NR12	*Crocodylus siamensis*	NR, Nakhon Ratchasima	LC754678	99.37	*C. siamensis*
43	NR13	*Crocodylus siamensis*	NR, Nakhon Ratchasima	LC754679	99.28	*C. siamensis*
44	NR14	*Crocodylus siamensis*	NR, Nakhon Ratchasima	LC754680	99.46	*C. siamensis*
45	NR15	*Crocodylus siamensis*	NR, Nakhon Ratchasima	LC754681	99.55	*C. siamensis*
46	NR16	*Crocodylus siamensis*	NR, Nakhon Ratchasima	LC754682	98.64	*C. siamensis*
47	NR17	*Crocodylus siamensis*	NR, Nakhon Ratchasima	LC754683	99.55	*C. siamensis*
48	NR18	*Crocodylus siamensis*	NR, Nakhon Ratchasima	LC754684	99.55	*C. siamensis*
49	NR19	*Crocodylus siamensis*	NR, Nakhon Ratchasima	LC754685	99.46	*C. siamensis*
50	NR20	*Crocodylus siamensis*	NR, Nakhon Ratchasima	LC754686	98.38	*C. siamensis*
51	NR21	*Crocodylus siamensis*	NR, Nakhon Ratchasima	LC754687	98.55	*C. siamensis*
52	NR22	*Crocodylus siamensis*	NR, Nakhon Ratchasima	LC754688	99.55	*C. siamensis*
53	NR23	*Crocodylus siamensis*	NR, Nakhon Ratchasima	LC754689	99.19	*C. siamensis*
54	NR24	*Crocodylus siamensis*	NR, Nakhon Ratchasima	LC754690	99.37	*C. siamensis*
55	NR25	*Crocodylus siamensis*	NR, Nakhon Ratchasima	LC754691	96.26	*C. siamensis*
56	NR26	*Crocodylus siamensis*	NR, Nakhon Ratchasima	LC754692	97.58	*C. siamensis*
57	NR27	*Crocodylus siamensis*	NR, Nakhon Ratchasima	LC754693	98.92	*C. siamensis*
58	NR28	*Crocodylus siamensis*	NR, Nakhon Ratchasima	LC754694	97.95	*C. siamensis*
59	NR29	*Crocodylus siamensis*	NR, Nakhon Ratchasima	LC754695	98.55	*C. siamensis*
60	NR30	*Crocodylus siamensis*	NR, Nakhon Ratchasima	LC754696	99.55	*C. siamensis*
61	CN01	*Crocodylus siamensis*	CN, Chainat	LC754697	99.55	*C. siamensis*
62	CN02	*Crocodylus siamensis*	CN, Chainat	LC754564	99.55	*C. siamensis*
63	CN03	*Crocodylus siamensis*	CN, Chainat	LC754565	98.13	*C. siamensis*
64	CN04	*Crocodylus siamensis*	CN, Chainat	LC754566	99.37	*C. siamensis*
65	CN05	*Crocodylus siamensis*	CN, Chainat	LC754567	98.04	*C. siamensis*
66	CN06	*Crocodylus siamensis*	CN, Chainat	LC754568	97.79	*C. siamensis*
67	CN07	*Crocodylus siamensis*	CN, Chainat	LC754569	98.75	*C. siamensis*
68	CN08	*Crocodylus siamensis*	CN, Chainat	LC754570	99.37	*C. siamensis*
69	CN09	*Crocodylus siamensis*	CN, Chainat	LC754571	98.83	*C. siamensis*
70	CN10	*Crocodylus siamensis*	CN, Chainat	LC754572	99.37	*C. siamensis*
71	CN11	*Crocodylus siamensis*	CN, Chainat	LC754573	99.55	*C. siamensis*
72	CN12	*Crocodylus siamensis*	CN, Chainat	LC754574	99.55	*C. siamensis*
73	CN13	*Crocodylus siamensis*	CN, Chainat	LC754575	98.74	*C. siamensis*
74	CN14	*Crocodylus siamensis*	CN, Chainat	LC754576	98.64	*C. siamensis*
75	CN15	*Crocodylus siamensis*	CN, Chainat	LC754577	99.55	*C. siamensis*
76	CN16	*Crocodylus siamensis*	CN, Chainat	LC754578	99.28	*C. siamensis*
77	CN17	*Crocodylus siamensis*	CN, Chainat	LC754579	99.01	*C. siamensis*
78	CN18	*Crocodylus siamensis*	CN, Chainat	LC754580	99.28	*C. siamensis*
79	CN19	*Crocodylus siamensis*	CN, Chainat	LC754581	96.19	*C. siamensis*
80	CN20	*Crocodylus siamensis*	CN, Chainat	LC754582	93.14	*C. siamensis*
81	CN21	*Crocodylus siamensis*	CN, Chainat	LC754583	98.12	*C. siamensis*
82	CN22	*Crocodylus siamensis*	CN, Chainat	LC754584	98.29	*C. siamensis*

No.	Abbreviation/ Code	species	Locality	Mitochondrial D-loop DDBJ accession number	Percent identity with species	
					Percent identity	Species
83	CN23	*Crocodylus siamensis*	CN, Chainat	LC754585	98.46	*C. siamensis*
84	CN24	*Crocodylus siamensis*	CN, Chainat	LC754586	99.46	*C. siamensis*
85	CN25	*Crocodylus siamensis*	CN, Chainat	LC754587	97.78	*C. siamensis*
86	CN26	*Crocodylus siamensis*	CN, Chainat	LC754588	96.09	*C. siamensis*
87	CN27	*Crocodylus siamensis*	CN, Chainat	LC754589	97.96	*C. siamensis*
88	CN28	*Crocodylus siamensis*	CN, Chainat	LC754590	99.28	*C. siamensis*
89	CN29	*Crocodylus siamensis*	CN, Chainat	LC754591	99.28	*C. siamensis*
90	CN30	*Crocodylus siamensis*	CN, Chainat	LC754592	99.37	*C. siamensis*
91	CN31	*Crocodylus siamensis*	CN, Chainat	LC754593	99.55	*C. siamensis*
92	CN32	*Crocodylus siamensis*	CN, Chainat	LC754594	99.46	*C. siamensis*
93	CN33	*Crocodylus siamensis*	CN, Chainat	LC754595	99.46	*C. siamensis*
94	CN34	*Crocodylus siamensis*	CN, Chainat	LC754596	99.55	*C. siamensis*
95	NP01	*Crocodylus siamensis*	NP, Nakhon Pathom	LC754626	99.46	*C. siamensis*
96	NP02	*Crocodylus siamensis*	NP, Nakhon Pathom	LC754627	98.29	*C. siamensis*
97	NP03	*Crocodylus siamensis*	NP, Nakhon Pathom	LC754628	98.64	*C. siamensis*
98	NP04	*Crocodylus siamensis*	NP, Nakhon Pathom	LC754629	97.08	*C. rhombifer*
99	NP05	*Crocodylus siamensis*	NP, Nakhon Pathom	LC754630	98.12	*C. siamensis*
100	NP06	*Crocodylus siamensis*	NP, Nakhon Pathom	LC754631	97.08	*C. rhombifer*
101	NP07	*Crocodylus siamensis*	NP, Nakhon Pathom	LC754632	97.00	*C. rhombifer*
102	NP08	*Crocodylus siamensis*	NP, Nakhon Pathom	LC754633	99.55	*C. siamensis*
103	NP09	*Crocodylus siamensis*	NP, Nakhon Pathom	LC754634	97.60	*C. rhombifer*
104	NP10	*Crocodylus siamensis*	NP, Nakhon Pathom	LC754635	99.55	*C. siamensis*
105	NP11	*Crocodylus siamensis*	NP, Nakhon Pathom	LC754636	99.55	*C. siamensis*
106	NP12	*Crocodylus siamensis*	NP, Nakhon Pathom	LC754637	98.92	*C. siamensis*
107	NP13	*Crocodylus siamensis*	NP, Nakhon Pathom	LC754638	97.59	*C. rhombifer*
108	NP14	*Crocodylus siamensis*	NP, Nakhon Pathom	LC754639	97.51	*C. rhombifer*
109	NP15	*Crocodylus siamensis*	NP, Nakhon Pathom	LC754640	99.46	*C. siamensis*
110	NP16	*Crocodylus siamensis*	NP, Nakhon Pathom	LC754641	97.42	*C. rhombifer*
111	NP17	*Crocodylus siamensis*	NP, Nakhon Pathom	LC754642	99.55	*C. siamensis*
112	NP18	*Crocodylus siamensis*	NP, Nakhon Pathom	LC754643	99.55	*C. siamensis*
113	NP19	*Crocodylus siamensis*	NP, Nakhon Pathom	LC754644	99.10	*C. siamensis*
114	NP20	*Crocodylus siamensis*	NP, Nakhon Pathom	LC754645	97.25	*C. rhombifer*
115	NP21	*Crocodylus siamensis*	NP, Nakhon Pathom	LC754646	99.37	*C. siamensis*
116	NP22	*Crocodylus siamensis*	NP, Nakhon Pathom	LC754647	98.47	*C. siamensis*
117	NP23	*Crocodylus siamensis*	NP, Nakhon Pathom	LC754648	98.72	*C. siamensis*
118	NP24	*Crocodylus siamensis*	NP, Nakhon Pathom	LC754649	98.72	*C. siamensis*
119	NP25	*Crocodylus siamensis*	NP, Nakhon Pathom	LC754650	98.55	*C. siamensis*
120	NP26	*Crocodylus siamensis*	NP, Nakhon Pathom	LC754651	98.72	*C. siamensis*
121	NP27	*Crocodylus siamensis*	NP, Nakhon Pathom	LC754652	99.83	*C. siamensis*
122	NP28	*Crocodylus siamensis*	NP, Nakhon Pathom	LC754653	99.73	*C. siamensis*
123	NP29	*Crocodylus siamensis*	NP, Nakhon Pathom	LC754654	98.83	*C. siamensis*
124	NP30	*Crocodylus siamensis*	NP, Nakhon Pathom	LC754655	98.64	*C. siamensis*
125	NP31	*Crocodylus siamensis*	NP, Nakhon Pathom	LC754656	97.87	*C. siamensis*
126	NP32	*Crocodylus siamensis*	NP, Nakhon Pathom	LC754657	98.55	*C. siamensis*

No.	Abbreviation/ Code	species	Locality	Mitochondrial D-loop DDBJ accession number	Percent identity with species	
					Percent identity	Species
127	NP33	*Crocodylus siamensis*	NP, Nakhon Pathom	LC754658	98.55	*C. siamensis*
128	NP34	*Crocodylus siamensis*	NP, Nakhon Pathom	LC754659	98.72	*C. siamensis*
129	NP35	*Crocodylus siamensis*	NP, Nakhon Pathom	LC754660	98.64	*C. siamensis*
130	NP36	*Crocodylus siamensis*	NP, Nakhon Pathom	LC754661	99.28	*C. siamensis*
131	NP37	*Crocodylus siamensis*	NP, Nakhon Pathom	LC754662	99.46	*C. siamensis*
132	NP38	*Crocodylus siamensis*	NP, Nakhon Pathom	LC754663	98.72	*C. siamensis*
133	NP39	*Crocodylus siamensis*	NP, Nakhon Pathom	LC754664	98.72	*C. siamensis*
134	NP40	*Crocodylus siamensis*	NP, Nakhon Pathom	LC754665	99.46	*C. siamensis*
135	NP41	*Crocodylus siamensis*	NP, Nakhon Pathom	LC754666	97.80	*C. siamensis*
136	NP42	*Crocodylus siamensis*	NP, Nakhon Pathom	LC754667	98.64	*C. siamensis*
137	CP01	*Crocodylus porosus*	CP, Nakon Ratchasima	LC754597	98.05	*C. porosus*
138	CP02	*Crocodylus porosus*	CP, Nakon Ratchasima	LC754598	95.64	*C. porosus*
139	CP03	*Crocodylus porosus*	CP, Nakon Ratchasima	LC754599	96.38	*C. porosus*
140	CP04	*Crocodylus porosus*	CP, Nakon Ratchasima	LC754600	95.97	*C. porosus*
141	CP05	*Crocodylus porosus*	CP, Nakon Ratchasima	LC754601	98.38	*C. siamensis*
142	CP06	*Crocodylus porosus*	CP, Nakon Ratchasima	LC754602	99.28	*C. siamensis*
143	CP07	*Crocodylus porosus*	CP, Nakon Ratchasima	LC754603	97.39	*C. porosus*
144	CP08	*Crocodylus porosus*	CP, Nakon Ratchasima	LC754604	97.05	*C. porosus*
145	CP10	*Crocodylus porosus*	CP, Nakon Ratchasima	LC754605	97.30	*C. porosus*
146	CP11	*Crocodylus porosus*	CP, Nakon Ratchasima	LC754606	98.74	*C. siamensis*
147	CP12	*Crocodylus porosus*	CP, Nakon Ratchasima	LC754607	98.03	*C. siamensis*
148	CP13	*Crocodylus porosus*	CP, Nakon Ratchasima	LC754608	98.47	*C. siamensis*
149	CP14	*Crocodylus porosus*	CP, Nakon Ratchasima	LC754609	96.09	*C. porosus*
150	CP15	*Crocodylus porosus*	CP, Nakon Ratchasima	LC754610	91.73	*C. siamensis*
151	CP16	*Crocodylus porosus*	CP, Nakon Ratchasima	LC754611	97.31	*C. porosus*
152	CP17	*Crocodylus porosus*	CP, Nakon Ratchasima	LC754612	98.20	*C. porosus*
153	CP18	*Crocodylus porosus*	CP, Nakon Ratchasima	LC754613	97.48	*C. porosus*
154	CP19	*Crocodylus porosus*	CP, Nakon Ratchasima	LC754614	96.13	*C. porosus*
155	CP20	*Crocodylus porosus*	CP, Nakon Ratchasima	LC754615	97.93	*C. siamensis*
156	CP21	*Crocodylus porosus*	CP, Nakon Ratchasima	LC754616	94.68	*C. porosus*
157	CP22	*Crocodylus porosus*	CP, Nakon Ratchasima	LC754617	95.83	*C. porosus*
158	CP23	*Crocodylus porosus*	CP, Nakon Ratchasima	LC754618	99.10	*C. siamensis*
159	CP24	*Crocodylus porosus*	CP, Nakon Ratchasima	LC754619	95.07	*C. porosus*
160	CP25	*Crocodylus porosus*	CP, Nakon Ratchasima	LC754620	98.02	*C. siamensis*
161	CP26	*Crocodylus porosus*	CP, Nakon Ratchasima	LC754621	97.13	*C. porosus*
162	CP27	*Crocodylus porosus*	CP, Nakon Ratchasima	LC754622	99.37	*C. siamensis*
163	CP28	*Crocodylus porosus*	CP, Nakon Ratchasima	LC754623	98.56	*C. siamensis*
164	CP29	*Crocodylus porosus*	CP, Nakon Ratchasima	LC754624	98.56	*C. siamensis*
165	CP30	*Crocodylus porosus*	CP, Nakon Ratchasima	LC754625	97.66	*C. siamensis*

Table S2 Microsatellite primers and sequences.

Primer	Fluorescence	Primer sequence 5′ to 3′		Size (bp)
		Reward	Forward	
**CpP 3001**	FAM	CAGTCGGGCGTCATCAGTAGCATGGCATAAGTGT	GTTTAGAATGCCATAAATCACAT	200–212
**CpP 208**	FAM	CAGTCGGGCGTCATCACACATGGCTTTTGTTCTGAG	GTTTCCTGCAAAATGTTCTCCTA	211–295
**CpP 501**	ROX	CAGCGGGCGTCATCACCTGATAGACTGCCTACAA	GTTTGTTAGTTCCCACTGAAGAAG	290–375
**CpP 1002**	ROX	CAGTCGGGCGTCATCATTTGGGCTCCACAATTC	GTTTCATCCCTTGGAGCTCTT	276–401
**CpP 214**	ROX	CAGTCGGGCGTCATCAGTCTGTCTTTGCAGTCTTG	GTTTTCCTGGAATAAACTGACTA	274–335
**CpP 209**	FAM	CAGTCGGGCGTCATCATACTGCTAATGCGAGACA	GTTTACTTAGGGTTTGCTAGTCAC	288–316
**CpP 2206**	ROX	CAGTCGGGCGTCATCAAAGTTCTCCCCACTAAAG	GTTTAGGCCAGTTCTTATCTACAT	258–270
**CpP 3313**	ROX	CAGTCGGGCGTCATCACTTCTGTTACTTAGGGACTG	GTTTAAAAACCCAGGCAAATA	360–368
**CpP 2504**	ROX	CAGTCGGGCGTCATCACTCATATTTCCCAACTATCAC	GTTTCATTCCCACAATACACATAA	306–367
**CpP 203**	ROX	CAGTCGGGCGTCATCAGTCCATTGCCAGTTGTAA	GTTTCTGAGGGTGAACTTTAGAA	205–283
**CpP 1308**	FAM	CAGTCGGGCGTCATCAACCTGAAAATGGATACTG	GTTTACGCTTGTTAACTTCACT	153–271
**CpP 4004**	FAM	CAGTCGGGCGTCATCACTGAATTGGGTGGAATAG	GTTTATCCACATTTTTCCATGAC	408–435
**CpP 3303**	FAM	CAGTCGGGCGTCATCATGTCTATTTTTGCCTTGTTT	GTTTGCCTAATTGCCTGAATAA	367–389
**CpP 509**	FAM	CAGTCGGGCGTCATCAACACAAAGGAGCATACAC	GTTTAGCCAATTCCCATATCT	328–339
**CpP 3008**	FAM	CAGTCGGGCGTCATCAACAACTGGCACATCTCA	GTTTCCCGTAGCCTCCTACTG	370–432
**CpP 2904**	FAM	CAGTCGGGCGTCATCAGGTTTCCTCCACACAA	GTTTCTATGCCACTAACATTAACAG	223–231
**CpP 4501**	FAM	CAGTCGGGCGTCATCATATACACACATGCACCTCTT	GTTTGCACAGCCCTAATAGA	101–113
**CpP 1201**	FAM	CAGTCGGGCGTCATCAGCAGATGTGGCAAATAGTTC	GTTTAAACTCGCCTCACTACAGA	123–141
**CpP 3004**	FAM	CAGTCGGGCGTCATCAGGAGTGAATCTATGCCAGC	GTTTAAAATGTTTTCATATGGTCG	142–156
**CpP 1409**	FAM	CAGTCGGGCGTCATCAGGGAAGGGGATTTAATAAT	GTTTATGCCCTACTGGTTATCTATC	255–289
**CpP 3508**	FAM	CAGTCGGGCGTCATCAAGGGGATGTAAACACT	GTTTATAACTATCATTGCCATTGT	117–128
**CpP 3219**	FAM	CAGTCGGGCGTCATCAGCAAGGGCATATAATAGTTC	GTTTAAGTTGCCTTAAAATTGTAAA	305–313

Table S3 Pairwise differentiation of linkage disequilibrium of Siamese crocodile (*Crocodylus siamensis*, Schneider, 1801) [34] individuals at Chonburi (CB) based on 22 microsatellite loci. Numbers indicate *p*-values with 110 permutations.

**Locus**	**CpP3001**	**CpP208**	**CpP501**	**CpP1002**	**CpP214**	**CpP209**	**CpP2206**	**CpP3313**	**CpP2504**	**CpP203**	**CpP1308**	CpP4004	CpP3303	CpP509	CpP3008	CpP2904	CpP4501	CpP1201	CpP3004	CpP1409	CpP3508	CpP3219
**CpP3001**																						
**CpP208**	0.034																					
**CpP501**	N/A	N/A																				
**CpP1002**	0.121	0.405	N/A																			
**CpP214**	0.101	0.344	N/A	0.169																		
**CpP209**	0.087	0.498	N/A	0.053	0.478																	
**CpP2206**	0.003	0.260	N/A	0.100	0.055	0.427																
**CpP3313**	0.528	0.112	N/A	0.691	1.000	0.115	0.778															
**CpP2504**	0.212	0.378	N/A	0.172	0.169	0.919	0.302	0.104														
**CpP203**	0.295	0.111	N/A	0.616	0.069	0.076	0.429	1.000	0.241													
**CpP1308**	0.001	0.174	N/A	0.478	0.082	0.086	0.038	0.874	0.025	0.942												
**CpP4004**	0.153	0.276	N/A	0.612	0.480	0.894	0.368	0.254	0.214	0.073	0.027											
**CpP3303**	0.000	0.049	N/A	0.110	0.013	0.252	0.073	0.565	0.054	0.422	0.006	0.026										
**CpP509**	0.106	0.743	N/A	0.331	0.267	0.591	0.619	0.344	0.017	0.011	0.116	0.052	0.089									
**CpP3008**	0.009	0.000	N/A	0.298	0.151	0.652	0.072	0.002	0.757	0.926	0.111	0.881	0.115	0.265								
**CpP2904**	0.000	0.013	N/A	0.509	0.721	0.025	0.004	0.576	0.725	0.773	0.006	0.492	0.006	0.485	0.016							
**CpP4501**	0.189	0.937	N/A	0.264	0.475	0.777	0.567	0.062	0.126	0.293	0.015	0.167	0.011	0.352	0.723	0.520						
**CpP1201**	0.243	0.764	N/A	0.418	0.432	0.278	0.333	0.468	0.780	0.648	0.191	0.641	0.694	0.410	0.432	0.719	0.278					
**CpP3004**	0.233	0.329	N/A	0.271	0.594	0.736	0.833	0.429	0.419	0.161	0.002	0.104	0.145	0.972	0.193	0.084	0.665	0.912				
**CpP1409**	0.788	0.759	N/A	0.276	0.638	0.693	0.477	0.367	0.854	0.352	0.813	0.883	0.226	0.851	0.783	0.572	0.040	0.850	0.020			
**CpP3508**	0.656	0.093	N/A	0.217	0.507	0.098	0.201	0.182	0.218	0.988	0.881	0.490	0.597	0.462	0.505	0.907	0.094	1.000	0.186	0.386		
CpP3219	0.847	0.709	N/A	0.571	0.667	0.486	0.390	0.887	0.028	0.803	0.062	0.135	0.592	0.432	0.407	0.898	0.001	0.115	0.434	0.089	0.264	

Table S4 Pairwise differentiation of linkage disequilibrium of Siamese crocodile (*Crocodylus siamensis*, Schneider, 1801) [34] individuals at Nakhon Ratchasima (NR) based on 22 microsatellite loci. Numbers indicate *p*-values with 110 permutations.

**Locus**	**CpP3001**	**CpP208**	**CpP501**	**CpP1002**	**CpP214**	**CpP209**	**CpP2206**	**CpP3313**	**CpP2504**	**CpP203**	**CpP1308**	CpP4004	CpP3303	CpP509	CpP3008	CpP2904	CpP4501	CpP1201	CpP3004	CpP1409	CpP3508	CpP3219
**CpP3001**																						
**CpP208**	0.323																					
**CpP501**	1.000	1.000																				
**CpP1002**	0.821	0.092	1.000																			
**CpP214**	0.986	0.625	1.000	0.889																		
**CpP209**	0.399	0.989	0.192	0.607	0.921																	
**CpP2206**	0.817	0.135	1.000	0.475	0.105	0.359																
**CpP3313**	0.980	0.735	1.000	0.870	1.000	0.515	0.282															
**CpP2504**	0.466	0.128	1.000	0.371	1.000	0.179	0.566	0.196														
**CpP203**	0.661	0.042	1.000	0.872	1.000	0.977	0.617	0.075	0.000													
**CpP1308**	0.429	0.204	0.689	0.306	0.358	0.662	0.013	0.511	0.212	0.882												
**CpP4004**	0.629	0.660	1.000	0.404	0.693	0.881	0.358	0.254	0.865	0.525	0.789											
**CpP3303**	0.933	0.212	0.614	0.065	0.494	0.435	0.240	0.170	0.912	0.897	0.563	0.331										
**CpP509**	0.177	0.402	1.000	0.673	0.835	0.195	0.125	0.804	0.722	0.644	0.380	0.044	0.216									
**CpP3008**	0.618	0.090	1.000	0.041	0.222	0.776	0.489	0.209	0.174	0.097	0.867	0.020	0.625	0.479								
**CpP2904**	0.906	0.474	1.000	0.303	0.371	0.997	0.015	0.582	0.566	0.732	0.000	0.882	0.174	0.567	0.950							
**CpP4501**	0.547	0.360	1.000	0.284	0.733	0.106	0.834	0.330	0.142	0.827	0.454	0.094	0.812	0.858	0.219	0.483						
**CpP1201**	0.776	0.095	1.000	0.893	0.362	0.335	0.222	0.743	0.437	0.838	0.355	0.287	0.301	0.027	0.396	0.234	0.673					
**CpP3004**	0.631	0.506	1.000	0.841	0.168	0.100	0.053	0.040	0.774	0.841	0.168	0.931	0.024	0.392	0.931	0.199	0.552	0.316				
**CpP1409**	0.254	0.093	1.000	0.368	0.678	0.285	0.876	0.730	0.429	0.997	0.516	0.560	0.879	0.568	0.941	0.489	0.413	0.353	0.410			
**CpP3508**	0.019	0.648	1.000	0.121	0.406	0.838	0.757	0.861	0.126	0.301	0.662	0.448	1.000	0.381	0.325	0.115	0.767	0.028	0.451	0.373		
CpP3219	0.389	0.243	1.000	0.072	0.834	0.374	0.982	0.684	0.069	0.324	0.852	0.067	0.911	0.796	0.137	0.555	0.256	0.611	1.000	0.163	0.393	

Table S5 Pairwise differentiation of linkage disequilibrium of Siamese crocodile (*Crocodylus siamensis*, Schneider, 1801) [34] individuals at Chainat (CN) based on 22 microsatellite loci. Numbers indicate *p*-values with 110 permutations.

**Locus**	**CpP3001**	**CpP208**	**CpP501**	**CpP1002**	**CpP214**	**CpP209**	**CpP2206**	**CpP3313**	**CpP2504**	**CpP203**	**CpP1308**	CpP4004	CpP3303	CpP509	CpP3008	CpP2904	CpP4501	CpP1201	CpP3004	CpP1409	CpP3508	CpP3219
**CpP3001**																						
**CpP208**	0.805																					
**CpP501**	0.547	0.639																				
**CpP1002**	1.000	0.743	0.978																			
**CpP214**	0.073	0.223	0.414	0.092																		
**CpP209**	0.041	0.064	0.768	0.175	0.534																	
**CpP2206**	0.553	0.947	0.171	0.923	0.385	0.738																
**CpP3313**	0.748	0.646	1.000	0.079	0.239	0.503	0.312															
**CpP2504**	0.853	0.878	1.000	0.346	0.087	0.220	0.191	1.000														
**CpP203**	0.656	0.036	1.000	0.294	1.000	0.481	1.000	1.000	1.000													
**CpP1308**	0.383	0.391	0.408	1.000	0.174	0.441	0.593	0.952	0.918	0.872												
**CpP4004**	0.339	0.070	0.241	0.390	0.284	0.739	0.822	0.201	0.854	1.000	0.001											
**CpP3303**	0.269	0.061	0.400	0.468	0.984	0.015	0.163	0.318	0.639	0.895	0.683	0.183										
**CpP509**	0.406	0.269	1.000	0.633	0.597	0.497	0.628	0.183	0.228	0.386	0.531	0.361	0.635									
**CpP3008**	0.489	0.890	0.714	0.494	0.769	0.137	0.611	0.951	0.050	1.000	0.918	0.813	0.258	0/000								
**CpP2904**	0.857	0.093	0.070	0.506	0.840	0.518	0.735	0.169	0.790	0.767	0.824	0.160	0.025	0.665	0.256							
**CpP4501**	0.253	0.419	0.736	0.085	0.479	0.218	0.270	0.265	0.747	0.616	0.789	0.051	0.246	0.562	0.100	0.399						
**CpP1201**	0.257	0.582	0.814	0.164	0.168	0.040	0.281	0.710	0.795	0.943	1.000	0.468	0.111	0.658	0.042	0.251	0.012					
**CpP3004**	0.0602	0.170	0.899	0.304	0.880	0.590	0.074	0.103	0.270	0.460	0.726	0.881	0.022	0.163	0.191	0.054	0.299	0.479				
**CpP1409**	0.537	0.834	0.162	0.001	0.010	0.007	0.639	0.486	0.789	0.431	0.842	0.059	0.109	0.966	0.750	0.015	0.249	0.017	0.382			
**CpP3508**	0.773	0.469	0.569	0.117	0.191	0.114	0.478	0.417	0.166	0.419	0.466	0.058	0.022	0.524	0.225	0.020	0.047	0.060	0.232	0.445		
CpP3219	0.035	0.940	0.114	0.505	0.687	0.962	0.336	1.000	0.822	0.593	0.704	0.678	0.256	0.277	0.826	0.801	0.401	0.489	0.339	0.484	0.674	

Table S6 Pairwise differentiation of linkage disequilibrium of Siamese crocodile (*Crocodylus siamensis*, Schneider, 1801) [34] individuals at Nakhon Pathom (NP) based on 22 microsatellite loci. Numbers indicate *p*-values with 110 permutations.

**Locus**	**CpP3001**	**CpP208**	**CpP501**	**CpP1002**	**CpP214**	**CpP209**	**CpP2206**	**CpP3313**	**CpP2504**	**CpP203**	**CpP1308**	CpP4004	CpP3303	CpP509	CpP3008	CpP2904	CpP4501	CpP1201	CpP3004	CpP1409	CpP3508	CpP3219
**CpP3001**																						
**CpP208**	0.178																					
**CpP501**	0.675	0.198																				
**CpP1002**	0.763	0.223	0.442																			
**CpP210**	0.583	0.873	0.035	0.041																		
**CpP209**	0.564	0.775	0.013	0.115	0.444																	
**CpP2206**	0.792	0.611	0.324	0.052	0.570	0.271																
**CpP3313**	0.119	0.477	0.000	0.408	0.660	0.008	0.429															
**CpP2504**	0.439	0.626	0.405	0.165	0.002	0.310	0.342	0.190														
**CpP203**	0.731	0.785	1.000	0.842	0.240	0.837	0.504	0.520	0.639													
**CpP1308**	0.700	0.528	0.657	0.588	0.061	0.454	0.467	0.607	0.469	0.149												
**CpP4004**	0.403	0.662	0.181	0.494	0.000	0.178	0.326	0.454	0.191	1.000	0.047											
**CpP3303**	0.544	0.763	0.045	0.288	0.318	0.141	0.096	0.512	0.290	0.050	0.253	0.120										
**CpP509**	0.275	0.778	0.434	0.531	0.074	0.025	0.522	0.020	0.285	0.000	0.336	0.334	0.041									
**CpP3008**	0.777	0.570	0.239	0.538	0.824	0.502	0.106	0.938	0.656	0.324	0.356	0.200	0.000	0.646								
**CpP2904**	0.179	0.250	0.132	0.058	0.315	0.311	0.176	0.611	0.204	0.318	0.923	0.662	0.118	0.021	0.235							
**CpP4501**	0.503	0.869	0.108	0.680	0.224	0.007	0.197	0.739	0.185	0.314	0.010	0.853	0.040	0.082	0.634	0.753						
**CpP1201**	0.059	0.287	0.866	1.000	0.761	0.225	0.835	0.225	0.511	0.185	0.031	0.436	0.230	0.945	0.653	0.267	0.316					
**CpP3004**	0.263	0.054	0.849	0.875	0.223	0.915	0.276	1.000	0.982	0.456	0.824	0.327	0.306	0.970	0.043	0.433	0.397	0.410				
**CpP1409**	0.013	0.781	0.431	0.133	0.353	0.626	0.629	0.673	0.686	0.701	0.442	0.193	0.619	0.274	0.744	0.330	0.499	0.777	0.197			
**CpP3508**	0.550	0.010	0.943	0.130	0.681	0.744	0.550	0.193	0.299	0.890	0.366	0.760	0.105	0.389	0.664	0.267	0.445	0.781	0.756	0.654		
CpP3219	0.701	0.979	0.149	0.194	0.404	0.463	0.250	0.984	0.000	0.712	0.213	0.313	0.412	0.915	0.370	0.730	0.862	0.358	0.547	0.568	0.492	

Table S7 Pairwise differentiation of linkage disequilibrium of saltwater crocodile (*Crocodylus porosus*, Schneider, 1801) [35] individuals at Nakhon Ratchasima (CP) based on 22 microsatellite loci. Numbers indicate *p*-values with 110 permutations.

**Locus**	**CpP3001**	**CpP208**	**CpP501**	**CpP1002**	**CpP214**	**CpP209**	**CpP2206**	**CpP3313**	**CpP2504**	**CpP203**	**CpP1308**	CpP4004	CpP3303	CpP509	CpP3008	CpP2904	CpP4501	CpP1201	CpP3004	CpP1409	CpP3508	CpP3219
**CpP3001**																						
**CpP208**	0.363																					
**CpP501**	0.379	0.570																				
**CpP1002**	0.134	0.337	0.127																			
**CpP214**	0.639	1.000	1.000	0.750																		
**CpP209**	0.275	1.000	1.000	0.214	1.000																	
**CpP2206**	0.737	0.194	0.884	0.154	o.246	0.267																
**CpP3313**	0.230	1.000	0.772	0.642	1.000	0.343	1.000															
**CpP2504**	0.014	1.000	0.596	0.639	1.000	0.270	0.320	0.393														
**CpP203**	0.610	0.099	1.000	1.000	1.000	1.000	1.000	1.000	1.000													
**CpP1308**	0.381	1.000	0.142	0.811	0.061	1.000	0.111	1.000	0.582	1.000												
**CpP4004**	0.284	1.000	0.433	0.487	0.358	0.269	0.121	0.150	0.000	1.000	0.000											
**CpP3303**	0.653	0.292	1.000	0.231	1.000	0.430	0.902	0.364	0.441	1.000	0.965	0.022										
**CpP509**	0.891	0.282	0.412	0.424	0.008	0.578	0.196	0.376	0.499	0.471	0.077	0.336	0.589									
**CpP3008**	0.675	0.530	1.000	0.696	1.000	0.168	0.464	1.000	0.208	1.000	0.342	0.074	0.027	0.566								
**CpP2904**	0.745	0.237	0.121	0.427	0.392	0.597	0.355	0.395	0.187	1.000	0.570	0.188	0.140	0.218	0.399							
**CpP4501**	0.216	0.860	0.774	0.760	0.870	0.104	0.828	0.518	0.338	0.557	0.849	0.838	0.549	0.770	0.431	0.373						
**CpP1201**	0.633	1.000	0.890	0.450	0.916	0.307	0.041	0.125	0.093	0.824	0.856	0.227	0.380	0.914	0.994	0.591	0.932					
**CpP3004**	0.263	0.734	0.069	0.008	1.000	0.082	0.119	0.965	0.287	0.301	0.789	0.701	0.829	0.435	0.301	0.298	0.375	0.149				
**CpP1409**	0.478	0.405	1.000	0.991	1.000	1.000	0.781	0.472	0.278	1.000	0.186	0.420	1.000	0.850	0.567	0.435	0.538	0.332	0.722			
**CpP3508**	0.526	0.473	0.654	0.017	0.678	0.426	0.015	0.686	0.052	0.052	1.000	0.762	0.730	0.091	0.545	0.351	0.468	0.966	0.425	0.331	0.774	
CpP3219	0.031	0.593	0.099	0.712	0.468	0.440	0.936	0.772	0.005	1.000	0.786	0.255	0.059	0.178	0.279	0.768	0.592	0.592	0.376	0.240	0.635	1.000

Table S8 Genetic diversity of 136 Siamese crocodile (*Crocodylus siamensis*, Schneider, 1801) [34] individuals and 29 Saltwater crocodile (*C. porosus*, Schneider, 1801) [35] individuals based on 22 microsatellite loci. Table S1 provides detailed information on the sampled individuals.

Population	Locus	N	N_a	*AR*	N_{ea}	*I*	H_o	H_e	*PIC*	*F*
CB [1]	CpP 3001	30	3.000	3.000	1.144	0.282	0.133	0.126	0.121	-0.057
	CpP 208	30	7.000	7.000	2.799	1.306	0.900	0.643	0.599	-0.400
	CpP 501	30	21.000	21.000	15.517	2.874	0.833	0.936	0.932	0.109
	CpP 1002	30	5.000	5.000	1.508	0.668	0.367	0.337	0.307	-0.089
	CpP 214	30	11.000	11.000	5.960	2.030	0.833	0.832	0.814	-0.001
	CpP 209	30	7.000	7.000	2.711	1.337	0.767	0.631	0.599	-0.215
	CpP 2206	30	6.000	6.000	2.769	1.260	0.667	0.639	0.586	-0.043
	CpP 3313	30	8.000	8.000	3.727	1.630	0.700	0.732	0.702	0.043
	CpP 2504	30	14.000	14.000	9.091	2.366	0.733	0.890	0.880	0.176
	CpP 203	30	17.000	17.000	8.411	2.441	0.767	0.881	0.872	0.130
	CpP 1308	30	5.000	5.000	2.158	0.900	0.500	0.537	0.439	0.068
	CpP 4004	30	9.000	9.000	4.905	1.797	0.833	0.796	0.769	-0.047
	CpP 3303	30	5.000	5.000	1.568	0.726	0.433	0.362	0.332	-0.196
	CpF 509	30	7.000	7.000	3.689	1.537	1.000	0.729	0.691	-0.372
	CpP 3008	30	7.000	7.000	3.000	1.326	0.733	0.667	0.607	-0.100
	CpP 2904	30	4.000	4.000	2.100	0.864	0.500	0.524	0.431	0.046
	CpP 4501	30	4.000	4.000	2.230	1.050	0.733	0.552	0.512	-0.329
	CpP 1201	30	3.000	3.000	1.439	0.532	0.367	0.305	0.268	-0.202
	CpP 3004	30	5.000	5.000	2.341	1.094	0.600	0.573	0.523	-0.048
	CpP 1409	30	6.000	6.000	2.961	1.260	0.467	0.662	0.602	0.295
	CpP 3508	30	2.000	2.000	1.897	0.666	0.767	0.473	0.361	-0.622
	CpP 3219	30	7.000	7.000	2.715	1.290	0.733	0.632	0.590	-0.161
	Mean	30	7.409	7.409	3.847	1.329	0.653	0.612	0.570	-0.092
	SE		0.993	0.993	0.713	0.140	0.044	0.044	0.214	0.045
NR [2]	CpP 3001	30	8.000	8.000	2.125	1.182	0.600	0.529	0.506	-0.133
	CpP 208	30	4.000	4.000	2.076	0.825	0.867	0.518	0.415	-0.672
	CpP 501	30	28.000	28.000	15.385	3.031	0.833	0.935	0.932	0.109
	CpP 1002	30	5.000	5.000	1.957	0.938	0.533	0.489	0.447	-0.091
	CpP 214	30	10.000	10.000	7.407	2.095	0.867	0.865	0.850	-0.002
	CpP 209	30	7.000	7.000	2.695	1.328	0.767	0.629	0.595	-0.219
	CpP 2206	30	5.000	5.000	1.648	0.822	0.367	0.393	0.371	0.068
	CpP 3313	30	12.000	12.000	4.337	1.838	0.767	0.769	0.744	0.004
	CpP 2504	30	19.000	19.000	6.294	2.329	0.300	0.841	0.826	0.643
	CpP 203	30	22.000	22.000	10.714	2.685	0.533	0.907	0.900	0.412
	CpP 1308	30	2.000	2.000	1.180	0.287	0.167	0.153	0.141	-0.091
	CpP 4004	27	8.000	8.000	3.367	1.484	0.704	0.703	0.658	-0.001
	CpP 3303	30	2.000	2.000	1.260	0.360	0.233	0.206	0.185	-0.132
	CpF 509	30	10.000	10.000	3.622	1.720	0.867	0.724	0.703	-0.197
	CpP 3008	30	9.000	9.000	3.237	1.490	0.867	0.691	0.643	-0.254
	CpP 2904	30	5.000	5.000	2.387	1.026	0.567	0.581	0.499	0.025

Population	Locus	N	N_a	*AR*	N_{ea}	*I*	H_o	H_e	*PIC*	*F*
	CpP 4501	30	4.000	4.000	2.011	0.896	0.533	0.503	0.439	-0.061
	CpP 1201	30	10.000	10.000	2.242	1.308	0.700	0.554	0.534	-0.264
	CpP 3004	30	5.000	5.000	2.329	1.133	0.600	0.571	0.532	-0.052
	CpP 1409	30	7.000	7.000	2.975	1.432	0.267	0.664	0.633	0.598
	CpP 3508	30	2.000	2.000	1.835	0.647	0.700	0.455	0.351	-0.538
	CpP 3219	30	8.000	8.000	3.550	1.556	0.600	0.718	0.683	0.165
	Mean	30	8.727	8.727	3.847	1.382	0.602	0.609	0.572	-0.031
	SE		1.405	1.405	0.728	0.149	0.047	0.043	0.213	0.066
CN [3]	CpP 3001	34	5.000	5.000	1.199	0.408	0.147	0.166	0.162	0.115
	CpP 208	34	2.000	2.000	1.778	0.630	0.647	0.438	0.342	-0.478
	CpP 501	34	24.000	24.000	15.728	2.924	0.912	0.936	0.933	0.026
	CpP 1002	34	5.000	5.000	1.128	0.305	0.088	0.113	0.112	0.221
	CpP 214	34	10.000	10.000	5.364	1.898	0.735	0.814	0.790	0.096
	CpP 209	34	8.000	8.000	2.809	1.372	0.618	0.644	0.607	0.041
	CpP 2206	34	8.000	8.000	2.312	1.071	0.588	0.567	0.486	-0.037
	CpP 3313	34	15.000	15.000	8.927	2.375	0.735	0.888	0.878	0.172
	CpP 2504	34	23.000	23.000	15.413	2.913	0.235	0.935	0.931	0.748
	CpP 203	34	22.000	22.000	14.360	2.855	0.824	0.930	0.926	0.115
	CpP 1308	34	2.000	2.000	1.192	0.298	0.176	0.161	0.148	-0.097
	CpP 4004	34	9.000	9.000	3.487	1.572	0.618	0.713	0.682	0.134
	CpP 3303	34	7.000	7.000	2.865	1.315	0.824	0.651	0.599	-0.265
	CpF 509	34	7.000	7.000	1.859	1.030	0.529	0.462	0.442	-0.146
	CpP 3008	34	12.000	12.000	6.266	2.068	0.882	0.840	0.822	-0.050
	CpP 2904	34	10.000	10.000	3.327	1.549	0.471	0.699	0.658	0.327
	CpP 4501	34	6.000	6.000	1.710	0.868	0.412	0.415	0.390	0.008
	CpP 1201	34	3.000	3.000	1.061	0.153	0.059	0.058	0.057	-0.023
	CpP 3004	34	9.000	9.000	2.269	1.312	0.382	0.559	0.540	0.316
	CpP 1409	34	6.000	6.000	1.948	1.010	0.500	0.487	0.452	-0.028
	CpP 3508	34	4.000	4.000	2.406	0.990	0.824	0.584	0.499	-0.409
	CpP 3219	34	12.000	12.000	3.866	1.794	0.794	0.741	0.719	-0.071
	Mean	34	9.500	9.500	4.603	1.396	0.545	0.582	0.553	0.033
	SE		1.364	1.364	1.000	0.180	0.057	0.058	0.268	0.055
NP [4]	CpP 3001	42	9.000	9.000	2.185	1.223	0.452	0.542	0.517	0.166
	CpP 208	42	10.000	10.000	2.602	1.271	0.881	0.616	0.551	-0.431
	CpP 501	42	21.000	21.000	12.923	2.786	0.738	0.923	0.918	0.200
	CpP 1002	42	5.000	5.000	1.187	0.393	0.071	0.158	0.154	0.547
	CpP 214	42	10.000	10.000	5.910	1.934	0.548	0.831	0.809	0.341
	CpP 209	42	9.000	9.000	4.860	1.789	0.857	0.794	0.767	-0.079
	CpP 2206	42	4.000	4.000	2.361	0.970	0.595	0.577	0.490	-0.032
	CpP 3313	42	14.000	14.000	7.113	2.261	0.929	0.859	0.847	-0.080
	CpP 2504	42	25.000	25.000	12.041	2.805	0.476	0.917	0.912	0.481
	CpP 203	42	22.000	22.000	11.529	2.710	0.714	0.913	0.907	0.218
	CpP 1308	42	6.000	6.000	2.015	0.925	0.571	0.504	0.435	-0.134

Population	Locus	N	N_a	*AR*	N_{ea}	*I*	H_o	H_e	*PIC*	*F*
	CpP 4004	42	16.000	16.000	6.278	2.189	0.643	0.841	0.824	0.235
	CpP 3303	42	8.000	8.000	1.872	0.914	0.571	0.466	0.411	-0.227
	CpF 509	42	12.000	12.000	5.910	2.047	0.833	0.831	0.813	-0.003
	CpP 3008	42	18.000	18.000	5.765	2.275	0.452	0.827	0.815	0.453
	CpP 2904	42	10.000	10.000	3.667	1.618	0.738	0.727	0.688	-0.015
	CpP 4501	42	7.000	7.000	2.645	1.318	0.643	0.622	0.587	-0.034
	CpP 1201	42	3.000	3.000	2.033	0.746	0.929	0.508	0.391	-0.827
	CpP 3004	42	5.000	5.000	2.623	1.167	0.667	0.619	0.554	-0.077
	CpP 1409	42	8.000	8.000	3.507	1.527	0.524	0.715	0.674	0.267
	CpP 3508	42	8.000	8.000	2.761	1.440	0.405	0.638	0.614	0.365
	CpP 3219	42	16.000	16.000	5.565	2.171	0.881	0.820	0.804	-0.074
	Mean	42	11.182	11.182	4.880	1.658	0.642	0.693	0.658	0.057
	SE		1.307	1.307	0.730	0.147	0.044	0.040	0.204	0.068
CP [5]	CpP 3001	29	3.000	3.000	1.639	0.697	0.241	0.390	0.350	0.381
	CpP 208	28	11.000	11.000	7.724	2.162	0.893	0.871	0.857	-0.026
	CpP 501	29	16.000	15.829	6.782	2.272	0.586	0.853	0.838	0.312
	CpP 1002	29	5.000	5.000	2.977	1.324	0.828	0.664	0.625	-0.246
	CpP 214	28	18.000	18.000	9.389	2.545	0.821	0.893	0.886	0.081
	CpP 209	29	9.000	8.900	5.160	1.803	0.690	0.806	0.779	0.145
	CpP 2206	29	10.000	9.899	5.006	1.886	0.759	0.800	0.779	0.052
	CpP 3313	28	12.000	12.000	6.938	2.127	0.714	0.856	0.840	0.165
	CpP 2504	29	13.000	12.833	4.216	1.943	0.517	0.763	0.747	0.322
	CpP 203	29	24.000	23.696	10.383	2.813	0.759	0.904	0.899	0.161
	CpP 1308	29	8.000	7.966	4.558	1.736	0.690	0.781	0.752	0.117
	CpP 4004	29	7.000	6.967	4.685	1.712	0.931	0.787	0.760	-0.184
	CpP 3303	29	6.000	5.999	4.335	1.575	0.828	0.769	0.732	-0.076
	CpF 509	28	6.000	6.000	3.824	1.479	0.929	0.739	0.696	-0.257
	CpP 3008	28	18.000	18.000	9.011	2.527	0.429	0.889	0.881	0.518
	CpP 2904	29	4.000	4.000	2.100	0.989	0.586	0.524	0.482	-0.119
	CpP 4501	29	4.000	3.967	2.875	1.128	0.862	0.652	0.583	-0.322
	CpP 1201	29	5.000	4.900	1.284	0.508	0.241	0.221	0.212	-0.091
	CpP 3004	29	10.000	10.799	3.534	1.570	0.724	0.717	0.673	-0.010
	CpP 1409	29	14.000	13.799	5.306	2.073	0.759	0.812	0.794	0.065
	CpP 3508	28	6.000	6.000	3.647	1.461	0.750	0.726	0.681	-0.033
	CpP 3219	28	7.000	7.000	3.540	1.432	0.786	0.717	0.668	-0.095
	Mean	29	9.818	9.798	4.951	1.716	0.696	0.733	0.705	0.039
	SE		1.169	1.150	0.530	0.125	0.041	0.036	0.173	0.046

Sample size (N); number of alleles (N_a); allelic richness (*AR*); number of effective alleles (N_{ea}); Shannon's information index (*I*); observed heterozygosity (H_o); expected heterozygosity (H_e); polymorphic information content (*PIC*); fixation index (*F*). [1] CB = Chonburi (*Crocodylus siamensis*). [2] NR = Nakhon Ratchasima (*Crocodylus siamensis*). [3] CN = Chainat (*Crocodylus siamensis*). [4] NP = Nakhon Pathom (*Crocodylus siamensis*). [5] CP = Nakhon Ratchasima (*Crocodylus porosus*).

Table S9 Pairwise genetic relatedness (*r*) for all 30 Siamese crocodile *(Crocodylus siamensis*, Schneider, 1801) [34] individuals in Chonburi (CB) Detailed information on all individuals is presented in Table S1

Sample 1	Sample 2	*r*
CB1	CB2	-0.005
CB1	CB3	-0.029
CB2	CB3	0.062
CB1	CB4	0.029
CB2	CB4	-0.053
CB3	CB4	-0.031
CB1	CB5	0.006
CB2	CB5	-0.048
CB3	CB5	0.002
CB4	CB5	-0.028
CB1	CB6	0.019
CB2	CB6	-0.058
CB3	CB6	0.015
CB4	CB6	-0.009
CB5	CB6	0.115
CB1	CB7	-0.041
CB2	CB7	-0.082
CB3	CB7	-0.015
CB4	CB7	-0.037
CB5	CB7	0.008
CB6	CB7	0.052
CB1	CB8	-0.044
CB2	CB8	-0.040
CB3	CB8	0.046
CB4	CB8	-0.023
CB5	CB8	-0.052
CB6	CB8	-0.052
CB7	CB8	0.001
CB1	CB9	0.013
CB2	CB9	-0.002
CB3	CB9	-0.027
CB4	CB9	0.145
CB5	CB9	-0.036
CB6	CB9	-0.031
CB7	CB9	-0.056
CB8	CB9	-0.029
CB1	CB10	-0.029
CB2	CB10	0.031
CB3	CB10	-0.051
CB4	CB10	-0.012
CB5	CB10	-0.025

Sample 1	Sample 2	*r*
CB6	CB10	-0.021
CB7	CB10	0.133
CB8	CB10	-0.042
CB9	CB10	-0.019
CB1	CB11	-0.051
CB2	CB11	-0.025
CB3	CB11	-0.090
CB4	CB11	0.004
CB5	CB11	-0.061
CB6	CB11	-0.064
CB7	CB11	-0.042
CB8	CB11	-0.070
CB9	CB11	-0.011
CB10	CB11	-0.009
CB1	CB12	-0.039
CB2	CB12	-0.032
CB3	CB12	-0.026
CB4	CB12	-0.006
CB5	CB12	-0.034
CB6	CB12	-0.042
CB7	CB12	0.020
CB8	CB12	0.048
CB9	CB12	0.009
CB10	CB12	0.002
CB11	CB12	-0.038
CB1	CB13	-0.010
CB2	CB13	0.008
CB3	CB13	0.006
CB4	CB13	0.067
CB5	CB13	0.007
CB6	CB13	0.005
CB7	CB13	-0.035
CB8	CB13	-0.006
CB9	CB13	0.078
CB10	CB13	-0.017
CB11	CB13	-0.008
CB12	CB13	0.022
CB1	CB14	0.013
CB2	CB14	-0.043
CB3	CB14	-0.029
CB4	CB14	-0.007
CB5	CB14	0.135
CB6	CB14	0.015

Sample 1	Sample 2	*r*
CB7	CB14	-0.003
CB8	CB14	-0.008
CB9	CB14	-0.021
CB10	CB14	-0.048
CB11	CB14	-0.060
CB12	CB14	-0.023
CB13	CB14	-0.042
CB1	CB15	-0.026
CB2	CB15	-0.003
CB3	CB15	0.051
CB4	CB15	-0.040
CB5	CB15	0.004
CB6	CB15	-0.031
CB7	CB15	-0.007
CB8	CB15	0.025
CB9	CB15	-0.042
CB10	CB15	0.018
CB11	CB15	-0.063
CB12	CB15	-0.028
CB13	CB15	-0.048
CB14	CB15	0.091
CB1	CB16	-0.032
CB2	CB16	-0.049
CB3	CB16	-0.018
CB4	CB16	-0.019
CB5	CB16	-0.003
CB6	CB16	-0.029
CB7	CB16	0.045
CB8	CB16	0.009
CB9	CB16	-0.001
CB10	CB16	0.052
CB11	CB16	-0.029
CB12	CB16	0.012
CB13	CB16	-0.027
CB14	CB16	0.020
CB15	CB16	0.077
CB1	CB17	-0.064
CB2	CB17	-0.052
CB3	CB17	0.000
CB4	CB17	-0.059
CB5	CB17	-0.047
CB6	CB17	-0.036
CB7	CB17	-0.004

**Sample 1**	**Sample 2**	***r***
CB8	CB17	0.037
CB9	CB17	-0.056
CB10	CB17	-0.048
CB11	CB17	-0.090
CB12	CB17	-0.020
CB13	CB17	-0.031
CB14	CB17	-0.056
CB15	CB17	0.002
CB16	CB17	-0.002
CB1	CB18	-0.062
CB2	CB18	0.019
CB3	CB18	0.016
CB4	CB18	-0.093
CB5	CB18	-0.061
CB6	CB18	-0.048
CB7	CB18	-0.007
CB8	CB18	0.122
CB9	CB18	-0.018
CB10	CB18	-0.051
CB11	CB18	-0.098
CB12	CB18	0.061
CB13	CB18	-0.024
CB14	CB18	-0.036
CB15	CB18	-0.033
CB16	CB18	-0.034
CB17	CB18	0.089
CB1	CB19	-0.054
CB2	CB19	-0.022
CB3	CB19	-0.042
CB4	CB19	-0.023
CB5	CB19	-0.044
CB6	CB19	-0.022
CB7	CB19	-0.020
CB8	CB19	0.007
CB9	CB19	-0.008
CB10	CB19	-0.027
CB11	CB19	-0.042
CB12	CB19	0.003
CB13	CB19	-0.047
CB14	CB19	0.001
CB15	CB19	-0.019
CB16	CB19	0.011
CB17	CB19	0.020

Sample 1	Sample 2	*r*
CB18	CB19	-0.017
CB1	CB20	-0.056
CB2	CB20	-0.016
CB3	CB20	-0.055
CB4	CB20	-0.059
CB5	CB20	-0.011
CB6	CB20	-0.005
CB7	CB20	-0.006
CB8	CB20	0.010
CB9	CB20	-0.014
CB10	CB20	0.011
CB11	CB20	-0.081
CB12	CB20	-0.017
CB13	CB20	-0.056
CB14	CB20	-0.006
CB15	CB20	-0.010
CB16	CB20	0.001
CB17	CB20	-0.015
CB18	CB20	0.016
CB19	CB20	0.139
CB1	CB21	-0.048
CB2	CB21	-0.012
CB3	CB21	-0.012
CB4	CB21	0.030
CB5	CB21	-0.030
CB6	CB21	-0.052
CB7	CB21	-0.011
CB8	CB21	-0.045
CB9	CB21	-0.014
CB10	CB21	-0.020
CB11	CB21	-0.018
CB12	CB21	-0.039
CB13	CB21	0.007
CB14	CB21	-0.035
CB15	CB21	-0.041
CB16	CB21	-0.003
CB17	CB21	-0.058
CB18	CB21	-0.055
CB19	CB21	-0.011
CB20	CB21	-0.031
CB1	CB22	-0.057
CB2	CB22	-0.035
CB3	CB22	-0.046

Sample 1	Sample 2	*r*
CB4	CB22	-0.046
CB5	CB22	-0.063
CB6	CB22	-0.062
CB7	CB22	-0.031
CB8	CB22	-0.054
CB9	CB22	-0.058
CB10	CB22	-0.011
CB11	CB22	0.314
CB12	CB22	-0.057
CB13	CB22	-0.074
CB14	CB22	-0.061
CB15	CB22	-0.057
CB16	CB22	-0.059
CB17	CB22	-0.078
CB18	CB22	-0.091
CB19	CB22	-0.022
CB20	CB22	-0.020
CB21	CB22	0.069
CB1	CB23	-0.076
CB2	CB23	-0.018
CB3	CB23	-0.071
CB4	CB23	-0.028
CB5	CB23	-0.049
CB6	CB23	-0.051
CB7	CB23	-0.005
CB8	CB23	0.004
CB9	CB23	-0.031
CB10	CB23	-0.023
CB11	CB23	-0.027
CB12	CB23	-0.006
CB13	CB23	-0.016
CB14	CB23	-0.027
CB15	CB23	0.040
CB16	CB23	0.031
CB17	CB23	0.058
CB18	CB23	0.003
CB19	CB23	0.021
CB20	CB23	-0.011
CB21	CB23	0.020
CB22	CB23	-0.077
CB1	CB24	-0.038
CB2	CB24	-0.010
CB3	CB24	-0.065

Sample 1	Sample 2	*r*
CB4	CB24	0.041
CB5	CB24	-0.019
CB6	CB24	-0.014
CB7	CB24	-0.030
CB8	CB24	-0.023
CB9	CB24	0.002
CB10	CB24	-0.012
CB11	CB24	-0.045
CB12	CB24	-0.017
CB13	CB24	0.001
CB14	CB24	-0.008
CB15	CB24	0.001
CB16	CB24	0.029
CB17	CB24	-0.028
CB18	CB24	-0.062
CB19	CB24	0.001
CB20	CB24	-0.024
CB21	CB24	0.004
CB22	CB24	-0.067
CB23	CB24	0.033
CB1	CB25	-0.075
CB2	CB25	-0.046
CB3	CB25	-0.017
CB4	CB25	-0.044
CB5	CB25	-0.015
CB6	CB25	-0.059
CB7	CB25	-0.039
CB8	CB25	0.082
CB9	CB25	-0.007
CB10	CB25	-0.054
CB11	CB25	-0.087
CB12	CB25	-0.049
CB13	CB25	0.029
CB14	CB25	-0.052
CB15	CB25	-0.035
CB16	CB25	-0.035
CB17	CB25	0.069
CB18	CB25	0.046
CB19	CB25	-0.005
CB20	CB25	-0.016
CB21	CB25	0.020
CB22	CB25	-0.088
CB23	CB25	0.029

Sample 1	Sample 2	*r*
CB24	CB25	-0.023
CB1	CB26	0.005
CB2	CB26	-0.030
CB3	CB26	-0.043
CB4	CB26	-0.012
CB5	CB26	-0.025
CB6	CB26	0.020
CB7	CB26	-0.036
CB8	CB26	-0.012
CB9	CB26	-0.007
CB10	CB26	-0.029
CB11	CB26	0.024
CB12	CB26	-0.014
CB13	CB26	-0.058
CB14	CB26	-0.007
CB15	CB26	-0.028
CB16	CB26	-0.003
CB17	CB26	-0.036
CB18	CB26	0.026
CB19	CB26	-0.016
CB20	CB26	-0.045
CB21	CB26	-0.033
CB22	CB26	-0.006
CB23	CB26	0.012
CB24	CB26	-0.026
CB25	CB26	-0.032
CB1	CB27	-0.064
CB2	CB27	-0.009
CB3	CB27	-0.047
CB4	CB27	-0.017
CB5	CB27	-0.076
CB6	CB27	-0.056
CB7	CB27	-0.069
CB8	CB27	-0.055
CB9	CB27	-0.034
CB10	CB27	-0.011
CB11	CB27	0.085
CB12	CB27	-0.012
CB13	CB27	-0.031
CB14	CB27	-0.069
CB15	CB27	-0.070
CB16	CB27	-0.060
CB17	CB27	-0.056

Sample 1	Sample 2	*r*
CB18	CB27	-0.053
CB19	CB27	-0.017
CB20	CB27	-0.040
CB21	CB27	-0.011
CB22	CB27	0.041
CB23	CB27	-0.040
CB24	CB27	-0.029
CB25	CB27	-0.046
CB26	CB27	-0.061
CB1	CB28	-0.038
CB2	CB28	-0.002
CB3	CB28	-0.071
CB4	CB28	-0.036
CB5	CB28	-0.038
CB6	CB28	-0.028
CB7	CB28	-0.023
CB8	CB28	-0.061
CB9	CB28	0.029
CB10	CB28	-0.016
CB11	CB28	-0.068
CB12	CB28	-0.025
CB13	CB28	-0.059
CB14	CB28	-0.041
CB15	CB28	-0.038
CB16	CB28	-0.021
CB17	CB28	-0.006
CB18	CB28	-0.026
CB19	CB28	0.027
CB20	CB28	-0.007
CB21	CB28	-0.031
CB22	CB28	-0.021
CB23	CB28	0.014
CB24	CB28	0.044
CB25	CB28	-0.063
CB26	CB28	-0.050
CB27	CB28	-0.052
CB1	CB29	-0.001
CB2	CB29	0.048
CB3	CB29	0.009
CB4	CB29	-0.030
CB5	CB29	-0.005
CB6	CB29	0.060
CB7	CB29	-0.035

Sample 1	Sample 2	*r*
CB8	CB29	-0.032
CB9	CB29	-0.036
CB10	CB29	-0.052
CB11	CB29	-0.045
CB12	CB29	-0.030
CB13	CB29	-0.053
CB14	CB29	0.017
CB15	CB29	-0.034
CB16	CB29	-0.028
CB17	CB29	-0.037
CB18	CB29	-0.008
CB19	CB29	-0.037
CB20	CB29	-0.042
CB21	CB29	-0.058
CB22	CB29	-0.045
CB23	CB29	-0.025
CB24	CB29	-0.026
CB25	CB29	0.016
CB26	CB29	0.056
CB27	CB29	-0.031
CB28	CB29	-0.061
CB1	CB30	-0.022
CB2	CB30	-0.005
CB3	CB30	-0.013
CB4	CB30	0.001
CB5	CB30	0.004
CB6	CB30	0.068
CB7	CB30	-0.023
CB8	CB30	-0.036
CB9	CB30	-0.075
CB10	CB30	-0.021
CB11	CB30	-0.067
CB12	CB30	-0.023
CB13	CB30	-0.049
CB14	CB30	-0.005
CB15	CB30	-0.017
CB16	CB30	-0.012
CB17	CB30	0.015
CB18	CB30	-0.044
CB19	CB30	-0.017
CB20	CB30	-0.006
CB21	CB30	-0.045
CB22	CB30	-0.041

**Sample 1**	**Sample 2**	***r***
CB23	CB30	-0.033
CB24	CB30	0.000
CB25	CB30	-0.033
CB26	CB30	-0.036
CB27	CB30	-0.019
CB28	CB30	-0.013
CB29	CB30	0.108

Table S10 Pairwise genetic relatedness (*r*) for all 30 Siamese crocodile *(Crocodylus siamensis*, Schneider, 1801*)* [34] individuals in Nakhon Ratchasima (NR) Table S1 provides detailed information on the sampled individuals.

Sample 1	Sample 2	*r*
NR1	NR2	-0.023
NR1	NR3	-0.017
NR2	NR3	-0.046
NR1	NR4	0.034
NR2	NR4	0.034
NR3	NR4	0.009
NR1	NR5	-0.066
NR2	NR5	-0.037
NR3	NR5	-0.035
NR4	NR5	0.039
NR1	NR6	-0.055
NR2	NR6	0.002
NR3	NR6	-0.036
NR4	NR6	0.031
NR5	NR6	0.059
NR1	NR7	-0.022
NR2	NR7	-0.042
NR3	NR7	0.024
NR4	NR7	-0.002
NR5	NR7	0.003
NR6	NR7	-0.022
NR1	NR8	-0.014
NR2	NR8	-0.033
NR3	NR8	-0.068
NR4	NR8	0.021
NR5	NR8	0.009
NR6	NR8	0.009
NR7	NR8	0.005
NR1	NR9	-0.045
NR2	NR9	-0.034
NR3	NR9	-0.059
NR4	NR9	-0.024
NR5	NR9	-0.028
NR6	NR9	0.019
NR7	NR9	0.017
NR8	NR9	0.016
NR1	NR10	-0.049
NR2	NR10	-0.036
NR3	NR10	-0.044
NR4	NR10	-0.020

**Sample 1**	**Sample 2**	***r***
NR5	NR10	-0.007
NR6	NR10	-0.029
NR7	NR10	-0.009
NR8	NR10	-0.028
NR9	NR10	0.004
NR1	NR11	-0.037
NR2	NR11	-0.034
NR3	NR11	-0.040
NR4	NR11	-0.064
NR5	NR11	-0.028
NR6	NR11	-0.005
NR7	NR11	-0.044
NR8	NR11	-0.027
NR9	NR11	-0.008
NR10	NR11	-0.046
NR1	NR12	-0.059
NR2	NR12	-0.048
NR3	NR12	-0.011
NR4	NR12	-0.041
NR5	NR12	-0.038
NR6	NR12	-0.013
NR7	NR12	-0.020
NR8	NR12	-0.013
NR9	NR12	-0.025
NR10	NR12	0.081
NR11	NR12	-0.002
NR1	NR13	-0.037
NR2	NR13	-0.031
NR3	NR13	-0.065
NR4	NR13	-0.047
NR5	NR13	-0.024
NR6	NR13	-0.047
NR7	NR13	-0.006
NR8	NR13	-0.028
NR9	NR13	-0.033
NR10	NR13	-0.009
NR11	NR13	-0.046
NR12	NR13	0.002
NR1	NR14	0.008
NR2	NR14	-0.020
NR3	NR14	-0.055
NR4	NR14	-0.022
NR5	NR14	-0.022

Sample 1	Sample 2	*r*
NR6	NR14	-0.032
NR7	NR14	-0.013
NR8	NR14	-0.027
NR9	NR14	-0.028
NR10	NR14	-0.009
NR11	NR14	-0.044
NR12	NR14	-0.050
NR13	NR14	-0.039
NR1	NR15	0.012
NR2	NR15	-0.018
NR3	NR15	-0.023
NR4	NR15	-0.035
NR5	NR15	-0.038
NR6	NR15	-0.046
NR7	NR15	-0.030
NR8	NR15	-0.010
NR9	NR15	-0.022
NR10	NR15	0.016
NR11	NR15	-0.008
NR12	NR15	-0.045
NR13	NR15	-0.032
NR14	NR15	0.018
NR1	NR16	-0.042
NR2	NR16	-0.038
NR3	NR16	0.033
NR4	NR16	0.005
NR5	NR16	-0.018
NR6	NR16	-0.035
NR7	NR16	0.009
NR8	NR16	-0.026
NR9	NR16	-0.014
NR10	NR16	-0.023
NR11	NR16	-0.022
NR12	NR16	-0.042
NR13	NR16	-0.051
NR14	NR16	0.002
NR15	NR16	0.041
NR1	NR17	-0.002
NR2	NR17	-0.012
NR3	NR17	0.005
NR4	NR17	-0.045
NR5	NR17	-0.054
NR6	NR17	-0.025

Sample 1	Sample 2	*r*
NR7	NR17	-0.049
NR8	NR17	-0.031
NR9	NR17	0.023
NR10	NR17	-0.049
NR11	NR17	-0.001
NR12	NR17	-0.025
NR13	NR17	-0.074
NR14	NR17	-0.037
NR15	NR17	-0.044
NR16	NR17	-0.014
NR1	NR18	-0.056
NR2	NR18	-0.054
NR3	NR18	-0.018
NR4	NR18	-0.040
NR5	NR18	-0.051
NR6	NR18	-0.035
NR7	NR18	0.005
NR8	NR18	-0.035
NR9	NR18	0.009
NR10	NR18	0.002
NR11	NR18	-0.034
NR12	NR18	-0.049
NR13	NR18	-0.031
NR14	NR18	-0.034
NR15	NR18	0.000
NR16	NR18	0.082
NR17	NR18	-0.064
NR1	NR19	-0.006
NR2	NR19	0.013
NR3	NR19	0.032
NR4	NR19	-0.023
NR5	NR19	-0.069
NR6	NR19	-0.023
NR7	NR19	-0.042
NR8	NR19	0.047
NR9	NR19	-0.029
NR10	NR19	-0.048
NR11	NR19	0.021
NR12	NR19	0.011
NR13	NR19	-0.059
NR14	NR19	-0.025
NR15	NR19	-0.008
NR16	NR19	-0.054

Sample 1	Sample 2	*r*
NR17	NR19	-0.002
NR18	NR19	0.018
NR1	NR20	-0.048
NR2	NR20	-0.015
NR3	NR20	-0.054
NR4	NR20	-0.030
NR5	NR20	-0.025
NR6	NR20	-0.046
NR7	NR20	0.013
NR8	NR20	0.013
NR9	NR20	0.029
NR10	NR20	-0.013
NR11	NR20	-0.041
NR12	NR20	0.004
NR13	NR20	-0.001
NR14	NR20	-0.021
NR15	NR20	0.005
NR16	NR20	-0.043
NR17	NR20	0.040
NR18	NR20	-0.027
NR19	NR20	0.009
NR1	NR21	-0.021
NR2	NR21	-0.002
NR3	NR21	-0.002
NR4	NR21	-0.035
NR5	NR21	-0.012
NR6	NR21	-0.048
NR7	NR21	0.016
NR8	NR21	-0.053
NR9	NR21	-0.050
NR10	NR21	-0.012
NR11	NR21	0.033
NR12	NR21	-0.051
NR13	NR21	0.000
NR14	NR21	-0.018
NR15	NR21	-0.047
NR16	NR21	-0.026
NR17	NR21	-0.002
NR18	NR21	0.003
NR19	NR21	-0.056
NR20	NR21	-0.013
NR1	NR22	-0.055
NR2	NR22	-0.055

**Sample 1**	**Sample 2**	***r***
NR3	NR22	-0.030
NR4	NR22	-0.039
NR5	NR22	-0.066
NR6	NR22	-0.054
NR7	NR22	0.008
NR8	NR22	-0.015
NR9	NR22	-0.062
NR10	NR22	0.005
NR11	NR22	-0.033
NR12	NR22	0.056
NR13	NR22	-0.011
NR14	NR22	-0.055
NR15	NR22	-0.027
NR16	NR22	-0.043
NR17	NR22	-0.047
NR18	NR22	-0.044
NR19	NR22	0.026
NR20	NR22	-0.037
NR21	NR22	-0.059
NR1	NR23	-0.023
NR2	NR23	-0.045
NR3	NR23	-0.009
NR4	NR23	-0.027
NR5	NR23	0.016
NR6	NR23	0.001
NR7	NR23	-0.017
NR8	NR23	-0.067
NR9	NR23	-0.009
NR10	NR23	-0.011
NR11	NR23	-0.059
NR12	NR23	0.001
NR13	NR23	-0.029
NR14	NR23	-0.021
NR15	NR23	-0.024
NR16	NR23	-0.051
NR17	NR23	-0.034
NR18	NR23	-0.015
NR19	NR23	0.047
NR20	NR23	-0.025
NR21	NR23	-0.048
NR22	NR23	-0.004
NR1	NR24	-0.070
NR2	NR24	-0.013

**Sample 1**	**Sample 2**	***r***
NR3	NR24	0.029
NR4	NR24	-0.043
NR5	NR24	-0.025
NR6	NR24	-0.022
NR7	NR24	-0.020
NR8	NR24	-0.043
NR9	NR24	-0.009
NR10	NR24	-0.001
NR11	NR24	0.019
NR12	NR24	0.029
NR13	NR24	-0.050
NR14	NR24	-0.036
NR15	NR24	-0.019
NR16	NR24	0.003
NR17	NR24	0.028
NR18	NR24	-0.053
NR19	NR24	-0.016
NR20	NR24	-0.003
NR21	NR24	-0.031
NR22	NR24	-0.020
NR23	NR24	0.028
NR1	NR25	-0.031
NR2	NR25	-0.002
NR3	NR25	-0.041
NR4	NR25	-0.051
NR5	NR25	-0.036
NR6	NR25	-0.024
NR7	NR25	-0.008
NR8	NR25	-0.046
NR9	NR25	-0.045
NR10	NR25	0.036
NR11	NR25	-0.026
NR12	NR25	-0.004
NR13	NR25	0.072
NR14	NR25	-0.040
NR15	NR25	-0.017
NR16	NR25	-0.044
NR17	NR25	-0.068
NR18	NR25	-0.037
NR19	NR25	-0.019
NR20	NR25	-0.020
NR21	NR25	-0.046
NR22	NR25	-0.049

Sample 1	Sample 2	*r*
NR23	NR25	0.008
NR24	NR25	-0.011
NR1	NR26	-0.045
NR2	NR26	-0.025
NR3	NR26	-0.045
NR4	NR26	-0.031
NR5	NR26	-0.033
NR6	NR26	-0.022
NR7	NR26	-0.012
NR8	NR26	-0.023
NR9	NR26	0.045
NR10	NR26	-0.032
NR11	NR26	-0.037
NR12	NR26	-0.025
NR13	NR26	-0.033
NR14	NR26	-0.029
NR15	NR26	-0.005
NR16	NR26	0.030
NR17	NR26	0.019
NR18	NR26	-0.012
NR19	NR26	-0.020
NR20	NR26	-0.047
NR21	NR26	-0.042
NR22	NR26	-0.056
NR23	NR26	0.013
NR24	NR26	0.097
NR25	NR26	-0.014
NR1	NR27	-0.076
NR2	NR27	-0.034
NR3	NR27	-0.050
NR4	NR27	-0.024
NR5	NR27	0.000
NR6	NR27	0.014
NR7	NR27	-0.004
NR8	NR27	-0.028
NR9	NR27	0.011
NR10	NR27	-0.003
NR11	NR27	-0.065
NR12	NR27	-0.042
NR13	NR27	-0.016
NR14	NR27	-0.019
NR15	NR27	0.033
NR16	NR27	-0.007

Sample 1	Sample 2	*r*
NR17	NR27	-0.047
NR18	NR27	0.002
NR19	NR27	-0.058
NR20	NR27	-0.021
NR21	NR27	-0.043
NR22	NR27	-0.061
NR23	NR27	0.031
NR24	NR27	-0.019
NR25	NR27	-0.021
NR26	NR27	0.027
NR1	NR28	-0.045
NR2	NR28	-0.033
NR3	NR28	-0.042
NR4	NR28	-0.026
NR5	NR28	0.049
NR6	NR28	0.004
NR7	NR28	-0.026
NR8	NR28	-0.008
NR9	NR28	0.030
NR10	NR28	0.009
NR11	NR28	-0.038
NR12	NR28	-0.013
NR13	NR28	-0.031
NR14	NR28	0.031
NR15	NR28	0.000
NR16	NR28	-0.001
NR17	NR28	-0.040
NR18	NR28	0.030
NR19	NR28	-0.019
NR20	NR28	-0.007
NR21	NR28	-0.026
NR22	NR28	-0.009
NR23	NR28	0.007
NR24	NR28	-0.027
NR25	NR28	-0.015
NR26	NR28	0.016
NR27	NR28	0.036
NR1	NR29	-0.020
NR2	NR29	-0.027
NR3	NR29	-0.065
NR4	NR29	-0.020
NR5	NR29	-0.034
NR6	NR29	-0.048

Sample 1	Sample 2	*r*
NR7	NR29	0.016
NR8	NR29	-0.044
NR9	NR29	-0.017
NR10	NR29	-0.016
NR11	NR29	-0.071
NR12	NR29	-0.058
NR13	NR29	-0.005
NR14	NR29	0.010
NR15	NR29	-0.027
NR16	NR29	-0.014
NR17	NR29	-0.003
NR18	NR29	0.094
NR19	NR29	-0.059
NR20	NR29	0.033
NR21	NR29	-0.014
NR22	NR29	-0.013
NR23	NR29	-0.037
NR24	NR29	-0.051
NR25	NR29	0.000
NR26	NR29	-0.027
NR27	NR29	0.033
NR28	NR29	-0.016
NR1	NR30	-0.054
NR2	NR30	-0.018
NR3	NR30	0.038
NR4	NR30	-0.028
NR5	NR30	-0.047
NR6	NR30	-0.017
NR7	NR30	0.003
NR8	NR30	0.057
NR9	NR30	-0.043
NR10	NR30	-0.040
NR11	NR30	0.038
NR12	NR30	0.058
NR13	NR30	-0.052
NR14	NR30	-0.034
NR15	NR30	0.011
NR16	NR30	0.008
NR17	NR30	-0.019
NR18	NR30	-0.040
NR19	NR30	0.003
NR20	NR30	-0.004
NR21	NR30	-0.052

**Sample 1**	**Sample 2**	***r***
NR22	NR30	0.001
NR23	NR30	-0.033
NR24	NR30	0.001
NR25	NR30	-0.039
NR26	NR30	-0.042
NR27	NR30	-0.017
NR28	NR30	-0.032
NR29	NR30	-0.054

Table S11 Pairwise genetic relatedness (*r*) for all 34 Siamese crocodile *(Crocodylus siamensis*, Schneider, 1801*)* [34] individuals in Chainat (CN). Table S1 provides detailed information on the sampled individuals.

Sample 1	Sample 2	*r*
CN01	CN02	-0.010
CN01	CN03	-0.034
CN02	CN03	-0.007
CN01	CN04	-0.005
CN02	CN04	0.016
CN03	CN04	0.062
CN01	CN05	-0.038
CN02	CN05	0.030
CN03	CN05	-0.005
CN04	CN05	0.085
CN01	CN06	0.014
CN02	CN06	0.008
CN03	CN06	0.013
CN04	CN06	0.007
CN05	CN06	0.053
CN01	CN07	-0.029
CN02	CN07	0.020
CN03	CN07	-0.028
CN04	CN07	-0.020
CN05	CN07	0.097
CN06	CN07	0.029
CN01	CN08	-0.020
CN02	CN08	-0.006
CN03	CN08	-0.023
CN04	CN08	-0.037
CN05	CN08	-0.030
CN06	CN08	-0.008
CN07	CN08	0.025
CN01	CN09	-0.019
CN02	CN09	0.014
CN03	CN09	-0.030
CN04	CN09	-0.037
CN05	CN09	-0.053
CN06	CN09	-0.027
CN07	CN09	-0.039
CN08	CN09	0.045
CN01	CN10	-0.042
CN02	CN10	0.008
CN03	CN10	-0.033
CN04	CN10	-0.029
CN05	CN10	-0.022

Sample 1	Sample 2	*r*
CN06	CN10	-0.030
CN07	CN10	-0.015
CN08	CN10	-0.014
CN09	CN10	-0.025
CN01	CN11	-0.012
CN02	CN11	0.033
CN03	CN11	-0.047
CN04	CN11	0.019
CN05	CN11	-0.056
CN06	CN11	-0.030
CN07	CN11	-0.059
CN08	CN11	-0.003
CN09	CN11	0.071
CN10	CN11	-0.039
CN01	CN12	-0.026
CN02	CN12	0.013
CN03	CN12	-0.011
CN04	CN12	-0.003
CN05	CN12	-0.049
CN06	CN12	-0.004
CN07	CN12	-0.014
CN08	CN12	-0.012
CN09	CN12	-0.010
CN10	CN12	0.014
CN11	CN12	-0.011
CN01	CN13	-0.028
CN02	CN13	-0.009
CN03	CN13	-0.023
CN04	CN13	0.001
CN05	CN13	0.007
CN06	CN13	-0.007
CN07	CN13	-0.044
CN08	CN13	-0.016
CN09	CN13	-0.029
CN10	CN13	-0.007
CN11	CN13	0.014
CN12	CN13	-0.021
CN01	CN14	-0.024
CN02	CN14	-0.025
CN03	CN14	-0.017
CN04	CN14	-0.026
CN05	CN14	-0.029
CN06	CN14	0.019

Sample 1	Sample 2	*r*
CN07	CN14	-0.050
CN08	CN14	-0.007
CN09	CN14	-0.008
CN10	CN14	-0.018
CN11	CN14	0.013
CN12	CN14	-0.016
CN13	CN14	0.089
CN01	CN15	-0.050
CN02	CN15	-0.045
CN03	CN15	-0.016
CN04	CN15	-0.001
CN05	CN15	-0.053
CN06	CN15	0.002
CN07	CN15	-0.038
CN08	CN15	-0.037
CN09	CN15	0.078
CN10	CN15	-0.015
CN11	CN15	-0.005
CN12	CN15	0.043
CN13	CN15	-0.046
CN14	CN15	0.018
CN01	CN16	-0.027
CN02	CN16	0.023
CN03	CN16	-0.037
CN04	CN16	-0.031
CN05	CN16	-0.038
CN06	CN16	-0.014
CN07	CN16	-0.037
CN08	CN16	-0.021
CN09	CN16	-0.004
CN10	CN16	0.026
CN11	CN16	-0.041
CN12	CN16	0.032
CN13	CN16	-0.014
CN14	CN16	-0.008
CN15	CN16	0.075
CN01	CN17	-0.014
CN02	CN17	-0.024
CN03	CN17	0.017
CN04	CN17	-0.005
CN05	CN17	-0.013
CN06	CN17	-0.028
CN07	CN17	-0.033

Sample 1	Sample 2	*r*
CN08	CN17	0.019
CN09	CN17	0.012
CN10	CN17	-0.023
CN11	CN17	-0.020
CN12	CN17	-0.028
CN13	CN17	0.005
CN14	CN17	0.020
CN15	CN17	-0.013
CN16	CN17	0.003
CN01	CN18	-0.026
CN02	CN18	0.003
CN03	CN18	-0.032
CN04	CN18	-0.052
CN05	CN18	-0.042
CN06	CN18	-0.023
CN07	CN18	-0.045
CN08	CN18	-0.034
CN09	CN18	-0.034
CN10	CN18	-0.007
CN11	CN18	-0.010
CN12	CN18	-0.013
CN13	CN18	-0.012
CN14	CN18	-0.045
CN15	CN18	-0.005
CN16	CN18	0.023
CN17	CN18	-0.006
CN01	CN19	-0.012
CN02	CN19	0.009
CN03	CN19	-0.018
CN04	CN19	-0.050
CN05	CN19	-0.023
CN06	CN19	0.051
CN07	CN19	-0.039
CN08	CN19	0.000
CN09	CN19	-0.010
CN10	CN19	-0.023
CN11	CN19	0.004
CN12	CN19	0.003
CN13	CN19	-0.008
CN14	CN19	-0.005
CN15	CN19	-0.027
CN16	CN19	-0.002
CN17	CN19	-0.028

Sample 1	Sample 2	*r*
CN18	CN19	0.053
CN01	CN20	-0.055
CN02	CN20	-0.046
CN03	CN20	-0.059
CN04	CN20	-0.045
CN05	CN20	-0.046
CN06	CN20	-0.057
CN07	CN20	-0.014
CN08	CN20	-0.062
CN09	CN20	-0.057
CN10	CN20	0.004
CN11	CN20	-0.061
CN12	CN20	-0.077
CN13	CN20	-0.047
CN14	CN20	-0.068
CN15	CN20	0.000
CN16	CN20	-0.045
CN17	CN20	-0.053
CN18	CN20	-0.073
CN19	CN20	-0.062
CN01	CN21	-0.058
CN02	CN21	0.038
CN03	CN21	-0.011
CN04	CN21	-0.033
CN05	CN21	-0.002
CN06	CN21	-0.023
CN07	CN21	0.011
CN08	CN21	-0.042
CN09	CN21	-0.038
CN10	CN21	0.021
CN11	CN21	-0.052
CN12	CN21	0.014
CN13	CN21	-0.034
CN14	CN21	-0.054
CN15	CN21	-0.046
CN16	CN21	-0.031
CN17	CN21	-0.020
CN18	CN21	0.010
CN19	CN21	-0.033
CN20	CN21	-0.026
CN01	CN22	-0.016
CN02	CN22	0.014
CN03	CN22	-0.019

**Sample 1**	**Sample 2**	***r***
CN04	CN22	-0.008
CN05	CN22	-0.027
CN06	CN22	0.077
CN07	CN22	-0.003
CN08	CN22	-0.031
CN09	CN22	-0.032
CN10	CN22	-0.035
CN11	CN22	0.012
CN12	CN22	0.016
CN13	CN22	-0.015
CN14	CN22	-0.050
CN15	CN22	-0.020
CN16	CN22	-0.043
CN17	CN22	-0.009
CN18	CN22	0.039
CN19	CN22	0.014
CN20	CN22	-0.054
CN21	CN22	0.029
CN01	CN23	-0.044
CN02	CN23	-0.011
CN03	CN23	-0.019
CN04	CN23	0.003
CN05	CN23	0.008
CN06	CN23	-0.032
CN07	CN23	0.044
CN08	CN23	-0.017
CN09	CN23	-0.022
CN10	CN23	-0.030
CN11	CN23	-0.011
CN12	CN23	-0.006
CN13	CN23	-0.024
CN14	CN23	-0.009
CN15	CN23	-0.043
CN16	CN23	-0.037
CN17	CN23	-0.025
CN18	CN23	-0.026
CN19	CN23	-0.015
CN20	CN23	-0.073
CN21	CN23	-0.030
CN22	CN23	-0.011
CN01	CN24	-0.053
CN02	CN24	-0.019
CN03	CN24	-0.036

Sample 1	Sample 2	*r*
CN04	CN24	-0.035
CN05	CN24	-0.040
CN06	CN24	-0.023
CN07	CN24	0.016
CN08	CN24	-0.023
CN09	CN24	-0.013
CN10	CN24	-0.003
CN11	CN24	-0.025
CN12	CN24	-0.010
CN13	CN24	-0.037
CN14	CN24	0.017
CN15	CN24	-0.016
CN16	CN24	-0.024
CN17	CN24	0.014
CN18	CN24	-0.022
CN19	CN24	-0.012
CN20	CN24	-0.057
CN21	CN24	0.000
CN22	CN24	-0.030
CN23	CN24	-0.002
CN01	CN25	-0.043
CN02	CN25	-0.034
CN03	CN25	-0.026
CN04	CN25	-0.036
CN05	CN25	-0.025
CN06	CN25	-0.037
CN07	CN25	-0.037
CN08	CN25	-0.056
CN09	CN25	-0.028
CN10	CN25	-0.055
CN11	CN25	-0.012
CN12	CN25	-0.011
CN13	CN25	-0.024
CN14	CN25	-0.047
CN15	CN25	-0.066
CN16	CN25	-0.022
CN17	CN25	-0.005
CN18	CN25	0.051
CN19	CN25	-0.005
CN20	CN25	-0.009
CN21	CN25	-0.008
CN22	CN25	0.039
CN23	CN25	-0.033

Sample 1	Sample 2	*r*
CN24	CN25	-0.007
CN01	CN26	-0.055
CN02	CN26	-0.005
CN03	CN26	-0.052
CN04	CN26	-0.009
CN05	CN26	-0.062
CN06	CN26	-0.043
CN07	CN26	-0.020
CN08	CN26	0.002
CN09	CN26	-0.025
CN10	CN26	-0.042
CN11	CN26	-0.015
CN12	CN26	-0.042
CN13	CN26	-0.043
CN14	CN26	-0.051
CN15	CN26	-0.040
CN16	CN26	-0.004
CN17	CN26	-0.028
CN18	CN26	0.060
CN19	CN26	0.011
CN20	CN26	-0.011
CN21	CN26	-0.032
CN22	CN26	0.004
CN23	CN26	-0.020
CN24	CN26	-0.026
CN25	CN26	0.001
CN01	CN27	-0.052
CN02	CN27	-0.044
CN03	CN27	-0.040
CN04	CN27	-0.032
CN05	CN27	-0.046
CN06	CN27	-0.030
CN07	CN27	-0.051
CN08	CN27	-0.003
CN09	CN27	-0.024
CN10	CN27	-0.020
CN11	CN27	0.026
CN12	CN27	-0.057
CN13	CN27	-0.035
CN14	CN27	0.002
CN15	CN27	-0.017
CN16	CN27	-0.035
CN17	CN27	-0.023

Sample 1	Sample 2	*r*
CN18	CN27	-0.058
CN19	CN27	-0.046
CN20	CN27	-0.030
CN21	CN27	-0.020
CN22	CN27	-0.036
CN23	CN27	-0.025
CN24	CN27	0.024
CN25	CN27	-0.052
CN26	CN27	0.017
CN01	CN28	-0.012
CN02	CN28	0.002
CN03	CN28	-0.050
CN04	CN28	-0.038
CN05	CN28	-0.019
CN06	CN28	-0.029
CN07	CN28	-0.004
CN08	CN28	-0.044
CN09	CN28	-0.014
CN10	CN28	-0.001
CN11	CN28	-0.017
CN12	CN28	-0.025
CN13	CN28	-0.011
CN14	CN28	-0.043
CN15	CN28	-0.052
CN16	CN28	-0.025
CN17	CN28	0.028
CN18	CN28	0.013
CN19	CN28	-0.036
CN20	CN28	-0.031
CN21	CN28	-0.028
CN22	CN28	0.054
CN23	CN28	-0.027
CN24	CN28	-0.005
CN25	CN28	0.122
CN26	CN28	-0.059
CN27	CN28	-0.039
CN01	CN29	-0.041
CN02	CN29	-0.027
CN03	CN29	-0.038
CN04	CN29	-0.042
CN05	CN29	-0.010
CN06	CN29	-0.050
CN07	CN29	-0.053

**Sample 1**	**Sample 2**	***r***
CN08	CN29	-0.053
CN09	CN29	-0.008
CN10	CN29	0.002
CN11	CN29	-0.020
CN12	CN29	-0.027
CN13	CN29	-0.028
CN14	CN29	0.018
CN15	CN29	-0.026
CN16	CN29	-0.018
CN17	CN29	0.020
CN18	CN29	0.003
CN19	CN29	0.050
CN20	CN29	-0.065
CN21	CN29	-0.029
CN22	CN29	-0.056
CN23	CN29	0.031
CN24	CN29	0.084
CN25	CN29	-0.007
CN26	CN29	-0.050
CN27	CN29	-0.043
CN28	CN29	-0.008
CN01	CN30	-0.015
CN02	CN30	0.030
CN03	CN30	-0.019
CN04	CN30	-0.040
CN05	CN30	-0.048
CN06	CN30	-0.013
CN07	CN30	-0.014
CN08	CN30	0.019
CN09	CN30	-0.011
CN10	CN30	0.000
CN11	CN30	-0.037
CN12	CN30	0.012
CN13	CN30	-0.042
CN14	CN30	-0.010
CN15	CN30	0.006
CN16	CN30	-0.008
CN17	CN30	-0.020
CN18	CN30	-0.033
CN19	CN30	-0.022
CN20	CN30	-0.062
CN21	CN30	0.003
CN22	CN30	-0.011

Sample 1	Sample 2	*r*
CN23	CN30	-0.040
CN24	CN30	-0.030
CN25	CN30	-0.052
CN26	CN30	-0.039
CN27	CN30	-0.043
CN28	CN30	0.000
CN29	CN30	0.009
CN01	CN31	-0.063
CN02	CN31	-0.042
CN03	CN31	-0.038
CN04	CN31	-0.049
CN05	CN31	-0.044
CN06	CN31	-0.031
CN07	CN31	0.013
CN08	CN31	-0.023
CN09	CN31	-0.025
CN10	CN31	-0.019
CN11	CN31	0.066
CN12	CN31	-0.020
CN13	CN31	-0.037
CN14	CN31	0.001
CN15	CN31	-0.006
CN16	CN31	-0.017
CN17	CN31	0.036
CN18	CN31	-0.019
CN19	CN31	-0.011
CN20	CN31	-0.067
CN21	CN31	-0.030
CN22	CN31	-0.024
CN23	CN31	-0.035
CN24	CN31	-0.018
CN25	CN31	-0.022
CN26	CN31	-0.045
CN27	CN31	0.012
CN28	CN31	-0.003
CN29	CN31	-0.015
CN30	CN31	0.002
CN01	CN32	-0.022
CN02	CN32	-0.061
CN03	CN32	-0.029
CN04	CN32	0.022
CN05	CN32	-0.027
CN06	CN32	-0.010

**Sample 1**	**Sample 2**	***r***
CN07	CN32	-0.016
CN08	CN32	-0.002
CN09	CN32	-0.051
CN10	CN32	-0.012
CN11	CN32	-0.054
CN12	CN32	-0.060
CN13	CN32	-0.055
CN14	CN32	-0.048
CN15	CN32	-0.024
CN16	CN32	-0.051
CN17	CN32	-0.033
CN18	CN32	-0.004
CN19	CN32	-0.010
CN20	CN32	-0.050
CN21	CN32	0.058
CN22	CN32	0.008
CN23	CN32	-0.062
CN24	CN32	-0.039
CN25	CN32	0.022
CN26	CN32	-0.070
CN27	CN32	-0.046
CN28	CN32	0.011
CN29	CN32	-0.040
CN30	CN32	-0.063
CN31	CN32	0.043
CN01	CN33	-0.051
CN02	CN33	-0.024
CN03	CN33	-0.030
CN04	CN33	0.026
CN05	CN33	0.001
CN06	CN33	-0.022
CN07	CN33	-0.021
CN08	CN33	-0.021
CN09	CN33	-0.046
CN10	CN33	-0.033
CN11	CN33	-0.049
CN12	CN33	-0.022
CN13	CN33	0.057
CN14	CN33	0.050
CN15	CN33	-0.059
CN16	CN33	-0.050
CN17	CN33	-0.012
CN18	CN33	0.043

Sample 1	Sample 2	*r*
CN19	CN33	-0.010
CN20	CN33	-0.049
CN21	CN33	-0.032
CN22	CN33	-0.032
CN23	CN33	0.046
CN24	CN33	-0.004
CN25	CN33	-0.027
CN26	CN33	-0.026
CN27	CN33	0.003
CN28	CN33	-0.032
CN29	CN33	-0.016
CN30	CN33	-0.028
CN31	CN33	-0.028
CN32	CN33	0.000
CN01	CN34	-0.024
CN02	CN34	-0.025
CN03	CN34	-0.038
CN04	CN34	-0.059
CN05	CN34	-0.004
CN06	CN34	-0.001
CN07	CN34	0.004
CN08	CN34	0.047
CN09	CN34	0.013
CN10	CN34	-0.048
CN11	CN34	0.009
CN12	CN34	-0.010
CN13	CN34	-0.036
CN14	CN34	-0.025
CN15	CN34	-0.067
CN16	CN34	-0.065
CN17	CN34	-0.046
CN18	CN34	-0.005
CN19	CN34	0.040
CN20	CN34	-0.059
CN21	CN34	-0.031
CN22	CN34	-0.018
CN23	CN34	-0.002
CN24	CN34	-0.006
CN25	CN34	0.005
CN26	CN34	-0.005
CN27	CN34	-0.011
CN28	CN34	-0.054
CN29	CN34	-0.003

**Sample 1**	**Sample 2**	***r***
CN30	CN34	-0.049
CN31	CN34	-0.017
CN32	CN34	-0.037
CN33	CN34	0.038

Table S12 Pairwise genetic relatedness (*r*) for all 42 Siamese crocodile *(Crocodylus siamensis*, Schneider, 1801*)* [34] individuals in Nakhon Pathom (NP). Table S1 provides detailed information on the sampled individuals.

Sample 1	Sample 2	*r*
NP01	NP02	-0.024
NP01	NP03	0.032
NP02	NP03	-0.004
NP01	NP04	0.027
NP02	NP04	-0.051
NP03	NP04	0.036
NP01	NP05	-0.010
NP02	NP05	-0.001
NP03	NP05	-0.040
NP04	NP05	-0.010
NP01	NP06	-0.020
NP02	NP06	-0.037
NP03	NP06	-0.050
NP04	NP06	0.042
NP05	NP06	0.077
NP01	NP07	-0.007
NP02	NP07	0.013
NP03	NP07	0.034
NP04	NP07	0.007
NP05	NP07	-0.024
NP06	NP07	-0.022
NP01	NP08	0.004
NP02	NP08	-0.020
NP03	NP08	-0.032
NP04	NP08	-0.058
NP05	NP08	-0.026
NP06	NP08	-0.049
NP07	NP08	-0.032
NP01	NP09	0.031
NP02	NP09	-0.021
NP03	NP09	-0.007
NP04	NP09	0.004
NP05	NP09	-0.032
NP06	NP09	0.028
NP07	NP09	0.109
NP08	NP09	0.067
NP01	NP10	0.074
NP02	NP10	-0.019
NP03	NP10	0.004
NP04	NP10	-0.044

Sample 1	Sample 2	*r*
NP05	NP10	0.072
NP06	NP10	-0.009
NP07	NP10	-0.012
NP08	NP10	-0.043
NP09	NP10	-0.029
NP01	NP11	-0.017
NP02	NP11	-0.025
NP03	NP11	0.003
NP04	NP11	-0.046
NP05	NP11	-0.029
NP06	NP11	-0.015
NP07	NP11	0.023
NP08	NP11	0.066
NP09	NP11	-0.014
NP10	NP11	-0.020
NP01	NP12	0.020
NP02	NP12	0.019
NP03	NP12	-0.012
NP04	NP12	-0.026
NP05	NP12	-0.005
NP06	NP12	0.016
NP07	NP12	-0.009
NP08	NP12	0.012
NP09	NP12	0.012
NP10	NP12	-0.033
NP11	NP12	0.009
NP01	NP13	-0.015
NP02	NP13	-0.023
NP03	NP13	-0.026
NP04	NP13	0.026
NP05	NP13	-0.023
NP06	NP13	-0.031
NP07	NP13	0.083
NP08	NP13	-0.009
NP09	NP13	0.130
NP10	NP13	-0.039
NP11	NP13	-0.029
NP12	NP13	0.018
NP01	NP14	-0.024
NP02	NP14	0.007
NP03	NP14	0.042
NP04	NP14	-0.021
NP05	NP14	-0.004

Sample 1	Sample 2	*r*
NP06	NP14	-0.028
NP07	NP14	0.032
NP08	NP14	0.046
NP09	NP14	0.060
NP10	NP14	-0.028
NP11	NP14	-0.007
NP12	NP14	0.057
NP13	NP14	0.159
NP01	NP15	-0.005
NP02	NP15	0.005
NP03	NP15	-0.018
NP04	NP15	-0.021
NP05	NP15	0.002
NP06	NP15	-0.024
NP07	NP15	-0.041
NP08	NP15	0.053
NP09	NP15	-0.036
NP10	NP15	-0.025
NP11	NP15	0.016
NP12	NP15	0.010
NP13	NP15	0.027
NP14	NP15	0.045
NP01	NP16	-0.020
NP02	NP16	-0.041
NP03	NP16	-0.027
NP04	NP16	0.094
NP05	NP16	-0.004
NP06	NP16	0.105
NP07	NP16	0.015
NP08	NP16	-0.022
NP09	NP16	0.022
NP10	NP16	0.013
NP11	NP16	-0.018
NP12	NP16	-0.038
NP13	NP16	0.055
NP14	NP16	-0.034
NP15	NP16	0.004
NP01	NP17	0.003
NP02	NP17	-0.039
NP03	NP17	-0.002
NP04	NP17	-0.063
NP05	NP17	-0.023
NP06	NP17	-0.038

**Sample 1**	**Sample 2**	***r***
NP07	NP17	-0.036
NP08	NP17	0.013
NP09	NP17	-0.052
NP10	NP17	-0.030
NP11	NP17	-0.004
NP12	NP17	-0.002
NP13	NP17	-0.048
NP14	NP17	-0.039
NP15	NP17	0.013
NP16	NP17	0.003
NP01	NP18	0.031
NP02	NP18	-0.031
NP03	NP18	0.045
NP04	NP18	-0.034
NP05	NP18	0.022
NP06	NP18	0.018
NP07	NP18	-0.002
NP08	NP18	-0.018
NP09	NP18	-0.025
NP10	NP18	0.037
NP11	NP18	-0.014
NP12	NP18	-0.019
NP13	NP18	-0.003
NP14	NP18	-0.040
NP15	NP18	-0.003
NP16	NP18	-0.014
NP17	NP18	0.044
NP01	NP19	-0.047
NP02	NP19	0.004
NP03	NP19	-0.044
NP04	NP19	-0.048
NP05	NP19	-0.038
NP06	NP19	-0.032
NP07	NP19	-0.044
NP08	NP19	0.011
NP09	NP19	-0.012
NP10	NP19	-0.023
NP11	NP19	0.023
NP12	NP19	0.006
NP13	NP19	-0.010
NP14	NP19	-0.043
NP15	NP19	-0.033
NP16	NP19	-0.057

Sample 1	Sample 2	*r*
NP17	NP19	-0.003
NP18	NP19	-0.038
NP01	NP20	-0.006
NP02	NP20	-0.026
NP03	NP20	-0.021
NP04	NP20	-0.020
NP05	NP20	-0.025
NP06	NP20	0.048
NP07	NP20	0.036
NP08	NP20	0.019
NP09	NP20	0.066
NP10	NP20	0.010
NP11	NP20	0.022
NP12	NP20	-0.008
NP13	NP20	-0.025
NP14	NP20	0.020
NP15	NP20	-0.034
NP16	NP20	0.017
NP17	NP20	0.078
NP18	NP20	0.004
NP19	NP20	-0.020
NP01	NP21	-0.040
NP02	NP21	-0.002
NP03	NP21	-0.022
NP04	NP21	-0.053
NP05	NP21	-0.025
NP06	NP21	-0.057
NP07	NP21	-0.064
NP08	NP21	-0.005
NP09	NP21	-0.049
NP10	NP21	-0.028
NP11	NP21	-0.006
NP12	NP21	0.002
NP13	NP21	-0.004
NP14	NP21	0.050
NP15	NP21	-0.043
NP16	NP21	0.005
NP17	NP21	-0.019
NP18	NP21	-0.035
NP19	NP21	-0.050
NP20	NP21	-0.047
NP01	NP22	-0.040
NP02	NP22	0.023

Sample 1	Sample 2	*r*
NP03	NP22	-0.031
NP04	NP22	0.015
NP05	NP22	-0.056
NP06	NP22	-0.054
NP07	NP22	-0.026
NP08	NP22	-0.043
NP09	NP22	-0.066
NP10	NP22	-0.038
NP11	NP22	-0.025
NP12	NP22	0.039
NP13	NP22	-0.052
NP14	NP22	-0.027
NP15	NP22	-0.040
NP16	NP22	-0.060
NP17	NP22	-0.033
NP18	NP22	-0.034
NP19	NP22	-0.011
NP20	NP22	-0.053
NP21	NP22	0.021
NP01	NP23	-0.028
NP02	NP23	-0.032
NP03	NP23	-0.031
NP04	NP23	-0.016
NP05	NP23	-0.031
NP06	NP23	-0.045
NP07	NP23	-0.043
NP08	NP23	-0.048
NP09	NP23	-0.049
NP10	NP23	-0.024
NP11	NP23	-0.039
NP12	NP23	-0.024
NP13	NP23	-0.050
NP14	NP23	-0.045
NP15	NP23	-0.038
NP16	NP23	-0.055
NP17	NP23	-0.006
NP18	NP23	-0.035
NP19	NP23	-0.026
NP20	NP23	-0.056
NP21	NP23	-0.013
NP22	NP23	0.063
NP01	NP24	-0.015
NP02	NP24	0.004

**Sample 1**	**Sample 2**	***r***
NP03	NP24	-0.008
NP04	NP24	-0.024
NP05	NP24	0.002
NP06	NP24	-0.034
NP07	NP24	-0.049
NP08	NP24	-0.056
NP09	NP24	-0.041
NP10	NP24	-0.022
NP11	NP24	-0.028
NP12	NP24	-0.032
NP13	NP24	-0.032
NP14	NP24	-0.045
NP15	NP24	-0.018
NP16	NP24	-0.058
NP17	NP24	0.016
NP18	NP24	0.000
NP19	NP24	0.004
NP20	NP24	-0.025
NP21	NP24	-0.019
NP22	NP24	0.055
NP23	NP24	0.036
NP01	NP25	-0.032
NP02	NP25	0.012
NP03	NP25	-0.025
NP04	NP25	-0.029
NP05	NP25	0.011
NP06	NP25	0.009
NP07	NP25	-0.026
NP08	NP25	-0.045
NP09	NP25	-0.039
NP10	NP25	-0.015
NP11	NP25	-0.037
NP12	NP25	-0.022
NP13	NP25	-0.032
NP14	NP25	-0.026
NP15	NP25	-0.007
NP16	NP25	-0.042
NP17	NP25	-0.018
NP18	NP25	-0.026
NP19	NP25	-0.019
NP20	NP25	-0.055
NP21	NP25	-0.022
NP22	NP25	-0.003

Sample 1	Sample 2	*r*
NP23	NP25	0.015
NP24	NP25	-0.010
NP01	NP26	-0.030
NP02	NP26	0.027
NP03	NP26	-0.029
NP04	NP26	-0.048
NP05	NP26	-0.013
NP06	NP26	-0.032
NP07	NP26	-0.029
NP08	NP26	-0.032
NP09	NP26	-0.018
NP10	NP26	-0.032
NP11	NP26	-0.018
NP12	NP26	-0.048
NP13	NP26	-0.035
NP14	NP26	-0.036
NP15	NP26	-0.011
NP16	NP26	-0.028
NP17	NP26	-0.024
NP18	NP26	0.021
NP19	NP26	-0.001
NP20	NP26	-0.046
NP21	NP26	-0.043
NP22	NP26	0.004
NP23	NP26	0.059
NP24	NP26	0.030
NP25	NP26	0.050
NP01	NP27	-0.044
NP02	NP27	-0.027
NP03	NP27	-0.028
NP04	NP27	-0.039
NP05	NP27	-0.035
NP06	NP27	-0.030
NP07	NP27	-0.049
NP08	NP27	-0.062
NP09	NP27	-0.068
NP10	NP27	-0.040
NP11	NP27	-0.034
NP12	NP27	-0.051
NP13	NP27	-0.053
NP14	NP27	-0.033
NP15	NP27	-0.047
NP16	NP27	-0.060

Sample 1	Sample 2	*r*
NP17	NP27	-0.031
NP18	NP27	-0.043
NP19	NP27	0.012
NP20	NP27	-0.006
NP21	NP27	-0.037
NP22	NP27	0.085
NP23	NP27	0.077
NP24	NP27	0.050
NP25	NP27	0.019
NP26	NP27	0.034
NP01	NP28	0.029
NP02	NP28	-0.012
NP03	NP28	0.011
NP04	NP28	-0.036
NP05	NP28	-0.023
NP06	NP28	-0.029
NP07	NP28	-0.046
NP08	NP28	-0.049
NP09	NP28	-0.058
NP10	NP28	0.038
NP11	NP28	-0.023
NP12	NP28	-0.029
NP13	NP28	-0.050
NP14	NP28	-0.030
NP15	NP28	0.035
NP16	NP28	-0.005
NP17	NP28	-0.024
NP18	NP28	-0.022
NP19	NP28	-0.045
NP20	NP28	-0.027
NP21	NP28	-0.025
NP22	NP28	-0.028
NP23	NP28	-0.036
NP24	NP28	-0.014
NP25	NP28	0.005
NP26	NP28	-0.033
NP27	NP28	0.011
NP01	NP29	-0.036
NP02	NP29	-0.003
NP03	NP29	-0.036
NP04	NP29	-0.039
NP05	NP29	-0.020
NP06	NP29	-0.057

Sample 1	Sample 2	*r*
NP07	NP29	-0.006
NP08	NP29	-0.017
NP09	NP29	-0.063
NP10	NP29	-0.055
NP11	NP29	-0.008
NP12	NP29	-0.035
NP13	NP29	-0.040
NP14	NP29	-0.049
NP15	NP29	0.006
NP16	NP29	-0.040
NP17	NP29	-0.044
NP18	NP29	-0.050
NP19	NP29	-0.062
NP20	NP29	-0.053
NP21	NP29	-0.020
NP22	NP29	0.059
NP23	NP29	0.017
NP24	NP29	-0.036
NP25	NP29	-0.004
NP26	NP29	0.010
NP27	NP29	0.016
NP28	NP29	-0.032
NP01	NP30	-0.049
NP02	NP30	0.012
NP03	NP30	-0.034
NP04	NP30	-0.042
NP05	NP30	0.024
NP06	NP30	-0.048
NP07	NP30	-0.045
NP08	NP30	-0.047
NP09	NP30	-0.056
NP10	NP30	-0.048
NP11	NP30	-0.029
NP12	NP30	-0.042
NP13	NP30	-0.051
NP14	NP30	-0.050
NP15	NP30	0.000
NP16	NP30	-0.045
NP17	NP30	-0.041
NP18	NP30	-0.052
NP19	NP30	-0.019
NP20	NP30	-0.053
NP21	NP30	-0.053

Sample 1	Sample 2	*r*
NP22	NP30	-0.036
NP23	NP30	0.031
NP24	NP30	-0.002
NP25	NP30	0.012
NP26	NP30	0.005
NP27	NP30	0.032
NP28	NP30	-0.042
NP29	NP30	0.156
NP01	NP31	-0.027
NP02	NP31	-0.007
NP03	NP31	-0.029
NP04	NP31	-0.050
NP05	NP31	-0.022
NP06	NP31	-0.040
NP07	NP31	-0.049
NP08	NP31	-0.046
NP09	NP31	-0.060
NP10	NP31	0.003
NP11	NP31	-0.038
NP12	NP31	-0.027
NP13	NP31	-0.048
NP14	NP31	-0.031
NP15	NP31	-0.010
NP16	NP31	-0.067
NP17	NP31	-0.010
NP18	NP31	-0.027
NP19	NP31	-0.028
NP20	NP31	-0.054
NP21	NP31	-0.038
NP22	NP31	0.020
NP23	NP31	0.036
NP24	NP31	0.011
NP25	NP31	0.080
NP26	NP31	-0.002
NP27	NP31	0.039
NP28	NP31	0.004
NP29	NP31	-0.005
NP30	NP31	0.012
NP01	NP32	-0.011
NP02	NP32	-0.020
NP03	NP32	-0.031
NP04	NP32	-0.031
NP05	NP32	-0.030

Sample 1	Sample 2	*r*
NP06	NP32	-0.010
NP07	NP32	-0.036
NP08	NP32	0.042
NP09	NP32	-0.015
NP10	NP32	-0.016
NP11	NP32	-0.017
NP12	NP32	-0.046
NP13	NP32	-0.047
NP14	NP32	-0.035
NP15	NP32	-0.016
NP16	NP32	-0.054
NP17	NP32	-0.020
NP18	NP32	-0.012
NP19	NP32	-0.014
NP20	NP32	-0.042
NP21	NP32	0.024
NP22	NP32	0.009
NP23	NP32	-0.012
NP24	NP32	-0.017
NP25	NP32	0.063
NP26	NP32	-0.030
NP27	NP32	0.007
NP28	NP32	-0.031
NP29	NP32	-0.011
NP30	NP32	-0.025
NP31	NP32	0.045
NP01	NP33	0.020
NP02	NP33	-0.013
NP03	NP33	0.031
NP04	NP33	-0.043
NP05	NP33	-0.033
NP06	NP33	-0.034
NP07	NP33	0.036
NP08	NP33	-0.044
NP09	NP33	-0.027
NP10	NP33	-0.012
NP11	NP33	-0.004
NP12	NP33	-0.044
NP13	NP33	-0.024
NP14	NP33	0.038
NP15	NP33	-0.046
NP16	NP33	-0.029
NP17	NP33	0.002

Sample 1	Sample 2	*r*
NP18	NP33	-0.029
NP19	NP33	-0.034
NP20	NP33	0.021
NP21	NP33	-0.041
NP22	NP33	-0.007
NP23	NP33	-0.017
NP24	NP33	-0.017
NP25	NP33	-0.019
NP26	NP33	-0.003
NP27	NP33	-0.013
NP28	NP33	-0.028
NP29	NP33	0.010
NP30	NP33	-0.020
NP31	NP33	0.033
NP32	NP33	-0.022
NP01	NP34	-0.023
NP02	NP34	-0.032
NP03	NP34	-0.041
NP04	NP34	-0.017
NP05	NP34	-0.048
NP06	NP34	-0.045
NP07	NP34	-0.028
NP08	NP34	-0.053
NP09	NP34	-0.060
NP10	NP34	-0.050
NP11	NP34	-0.053
NP12	NP34	-0.012
NP13	NP34	-0.047
NP14	NP34	-0.041
NP15	NP34	-0.016
NP16	NP34	-0.074
NP17	NP34	0.015
NP18	NP34	-0.059
NP19	NP34	-0.044
NP20	NP34	-0.023
NP21	NP34	-0.064
NP22	NP34	-0.025
NP23	NP34	-0.004
NP24	NP34	-0.006
NP25	NP34	-0.007
NP26	NP34	-0.004
NP27	NP34	0.007
NP28	NP34	-0.022

Sample 1	Sample 2	*r*
NP29	NP34	0.066
NP30	NP34	0.036
NP31	NP34	0.042
NP32	NP34	-0.021
NP33	NP34	-0.008
NP01	NP35	-0.017
NP02	NP35	-0.032
NP03	NP35	-0.031
NP04	NP35	-0.058
NP05	NP35	0.049
NP06	NP35	0.049
NP07	NP35	-0.047
NP08	NP35	-0.050
NP09	NP35	-0.056
NP10	NP35	-0.027
NP11	NP35	-0.051
NP12	NP35	0.002
NP13	NP35	-0.051
NP14	NP35	-0.040
NP15	NP35	-0.030
NP16	NP35	-0.055
NP17	NP35	-0.028
NP18	NP35	-0.030
NP19	NP35	-0.029
NP20	NP35	-0.025
NP21	NP35	-0.046
NP22	NP35	0.047
NP23	NP35	-0.008
NP24	NP35	0.005
NP25	NP35	0.037
NP26	NP35	-0.024
NP27	NP35	0.020
NP28	NP35	-0.023
NP29	NP35	-0.028
NP30	NP35	0.009
NP31	NP35	0.048
NP32	NP35	0.009
NP33	NP35	-0.028
NP34	NP35	0.030
NP01	NP36	-0.007
NP02	NP36	-0.025
NP03	NP36	-0.041
NP04	NP36	0.019

**Sample 1**	**Sample 2**	***r***
NP05	NP36	-0.048
NP06	NP36	-0.033
NP07	NP36	-0.024
NP08	NP36	-0.023
NP09	NP36	-0.063
NP10	NP36	0.005
NP11	NP36	0.001
NP12	NP36	-0.007
NP13	NP36	-0.050
NP14	NP36	-0.035
NP15	NP36	-0.003
NP16	NP36	-0.041
NP17	NP36	-0.045
NP18	NP36	-0.029
NP19	NP36	-0.007
NP20	NP36	-0.029
NP21	NP36	0.012
NP22	NP36	0.022
NP23	NP36	-0.018
NP24	NP36	-0.035
NP25	NP36	-0.041
NP26	NP36	-0.045
NP27	NP36	-0.038
NP28	NP36	-0.009
NP29	NP36	-0.030
NP30	NP36	0.006
NP31	NP36	-0.005
NP32	NP36	0.019
NP33	NP36	-0.022
NP34	NP36	-0.019
NP35	NP36	0.006
NP01	NP37	-0.002
NP02	NP37	-0.026
NP03	NP37	-0.048
NP04	NP37	0.005
NP05	NP37	-0.036
NP06	NP37	-0.032
NP07	NP37	-0.017
NP08	NP37	-0.011
NP09	NP37	-0.048
NP10	NP37	-0.010
NP11	NP37	-0.014
NP12	NP37	-0.031

Sample 1	Sample 2	*r*
NP13	NP37	-0.017
NP14	NP37	-0.022
NP15	NP37	-0.018
NP16	NP37	-0.024
NP17	NP37	-0.058
NP18	NP37	-0.014
NP19	NP37	-0.043
NP20	NP37	0.071
NP21	NP37	0.014
NP22	NP37	-0.044
NP23	NP37	-0.044
NP24	NP37	-0.055
NP25	NP37	-0.052
NP26	NP37	-0.060
NP27	NP37	-0.049
NP28	NP37	0.036
NP29	NP37	-0.030
NP30	NP37	-0.010
NP31	NP37	-0.023
NP32	NP37	0.024
NP33	NP37	0.012
NP34	NP37	-0.039
NP35	NP37	-0.025
NP36	NP37	0.107
NP01	NP38	-0.034
NP02	NP38	-0.013
NP03	NP38	-0.007
NP04	NP38	-0.016
NP05	NP38	-0.024
NP06	NP38	-0.031
NP07	NP38	-0.035
NP08	NP38	-0.023
NP09	NP38	-0.051
NP10	NP38	-0.012
NP11	NP38	-0.024
NP12	NP38	0.009
NP13	NP38	-0.038
NP14	NP38	-0.041
NP15	NP38	-0.027
NP16	NP38	-0.056
NP17	NP38	-0.023
NP18	NP38	-0.033
NP19	NP38	0.012

Sample 1	Sample 2	*r*
NP20	NP38	-0.043
NP21	NP38	-0.023
NP22	NP38	-0.034
NP23	NP38	0.021
NP24	NP38	0.001
NP25	NP38	-0.017
NP26	NP38	-0.001
NP27	NP38	0.022
NP28	NP38	-0.031
NP29	NP38	0.003
NP30	NP38	0.104
NP31	NP38	0.042
NP32	NP38	0.004
NP33	NP38	-0.010
NP34	NP38	0.011
NP35	NP38	0.040
NP36	NP38	0.047
NP37	NP38	-0.026
NP01	NP39	-0.028
NP02	NP39	-0.026
NP03	NP39	-0.028
NP04	NP39	-0.027
NP05	NP39	-0.014
NP06	NP39	-0.028
NP07	NP39	-0.030
NP08	NP39	-0.034
NP09	NP39	-0.044
NP10	NP39	0.002
NP11	NP39	-0.024
NP12	NP39	-0.010
NP13	NP39	-0.027
NP14	NP39	-0.026
NP15	NP39	-0.021
NP16	NP39	-0.058
NP17	NP39	-0.007
NP18	NP39	-0.015
NP19	NP39	-0.003
NP20	NP39	-0.019
NP21	NP39	-0.032
NP22	NP39	0.005
NP23	NP39	0.018
NP24	NP39	0.051
NP25	NP39	0.011

Sample 1	Sample 2	*r*
NP26	NP39	-0.006
NP27	NP39	0.067
NP28	NP39	-0.013
NP29	NP39	-0.026
NP30	NP39	0.011
NP31	NP39	0.055
NP32	NP39	0.007
NP33	NP39	-0.023
NP34	NP39	-0.004
NP35	NP39	0.022
NP36	NP39	-0.002
NP37	NP39	-0.045
NP38	NP39	0.034
NP01	NP40	-0.045
NP02	NP40	-0.036
NP03	NP40	0.070
NP04	NP40	-0.019
NP05	NP40	-0.041
NP06	NP40	0.001
NP07	NP40	-0.054
NP08	NP40	-0.039
NP09	NP40	-0.044
NP10	NP40	0.021
NP11	NP40	-0.022
NP12	NP40	-0.012
NP13	NP40	-0.036
NP14	NP40	-0.018
NP15	NP40	-0.045
NP16	NP40	-0.029
NP17	NP40	0.004
NP18	NP40	-0.021
NP19	NP40	0.024
NP20	NP40	-0.013
NP21	NP40	-0.031
NP22	NP40	-0.032
NP23	NP40	-0.035
NP24	NP40	-0.013
NP25	NP40	0.019
NP26	NP40	-0.032
NP27	NP40	-0.012
NP28	NP40	-0.033
NP29	NP40	-0.046
NP30	NP40	-0.060

Sample 1	Sample 2	*r*
NP31	NP40	-0.004
NP32	NP40	-0.014
NP33	NP40	-0.037
NP34	NP40	-0.048
NP35	NP40	-0.023
NP36	NP40	-0.009
NP37	NP40	-0.037
NP38	NP40	-0.006
NP39	NP40	0.015
NP01	NP41	-0.020
NP02	NP41	-0.009
NP03	NP41	0.000
NP04	NP41	-0.036
NP05	NP41	-0.048
NP06	NP41	-0.045
NP07	NP41	-0.050
NP08	NP41	0.025
NP09	NP41	-0.047
NP10	NP41	0.000
NP11	NP41	-0.011
NP12	NP41	0.006
NP13	NP41	-0.040
NP14	NP41	-0.031
NP15	NP41	-0.010
NP16	NP41	-0.042
NP17	NP41	-0.020
NP18	NP41	-0.043
NP19	NP41	-0.020
NP20	NP41	-0.061
NP21	NP41	0.039
NP22	NP41	0.034
NP23	NP41	-0.024
NP24	NP41	-0.026
NP25	NP41	-0.019
NP26	NP41	-0.041
NP27	NP41	0.006
NP28	NP41	0.025
NP29	NP41	0.080
NP30	NP41	-0.010
NP31	NP41	0.029
NP32	NP41	0.024
NP33	NP41	-0.019
NP34	NP41	0.006

**Sample 1**	**Sample 2**	***r***
NP35	NP41	-0.007
NP36	NP41	0.039
NP37	NP41	-0.039
NP38	NP41	0.035
NP39	NP41	-0.007
NP40	NP41	0.027
NP01	NP42	-0.015
NP02	NP42	0.033
NP03	NP42	-0.035
NP04	NP42	-0.035
NP05	NP42	-0.014
NP06	NP42	-0.005
NP07	NP42	-0.025
NP08	NP42	-0.029
NP09	NP42	-0.024
NP10	NP42	-0.017
NP11	NP42	-0.011
NP12	NP42	-0.030
NP13	NP42	-0.031
NP14	NP42	-0.030
NP15	NP42	-0.048
NP16	NP42	-0.055
NP17	NP42	-0.028
NP18	NP42	-0.043
NP19	NP42	-0.041
NP20	NP42	-0.018
NP21	NP42	-0.034
NP22	NP42	0.011
NP23	NP42	0.038
NP24	NP42	-0.025
NP25	NP42	0.030
NP26	NP42	0.011
NP27	NP42	0.045
NP28	NP42	-0.052
NP29	NP42	-0.028
NP30	NP42	0.060
NP31	NP42	0.010
NP32	NP42	0.054
NP33	NP42	-0.004
NP34	NP42	0.059
NP35	NP42	-0.003
NP36	NP42	-0.014
NP37	NP42	-0.043

**Sample 1**	**Sample 2**	***r***
NP38	NP42	0.008
NP39	NP42	0.045
NP40	NP42	-0.042
NP41	NP42	-0.035

Table S13 Pairwise genetic relatedness (*r*) for all 29 Saltwater crocodile *(Crocodylus porosus*, Schneider, 1801*)* [35] individuals in Nakhon Ratchasima (CP). Table S1 provides detailed information on the sampled individuals.

Sample 1	Sample 2	*r*
CP01	CP02	-0.037
CP01	CP03	-0.051
CP02	CP03	-0.020
CP01	CP04	0.011
CP02	CP04	-0.021
CP03	CP04	-0.036
CP01	CP05	0.002
CP02	CP05	-0.046
CP03	CP05	-0.025
CP04	CP05	-0.010
CP01	CP06	-0.017
CP02	CP06	-0.051
CP03	CP06	-0.017
CP04	CP06	-0.009
CP05	CP06	0.056
CP01	CP07	-0.050
CP02	CP07	-0.019
CP03	CP07	0.026
CP04	CP07	-0.048
CP05	CP07	-0.026
CP06	CP07	-0.013
CP01	CP08	-0.039
CP02	CP08	-0.014
CP03	CP08	-0.029
CP04	CP08	-0.025
CP05	CP08	-0.027
CP06	CP08	-0.023
CP07	CP08	0.040
CP01	CP10	-0.008
CP02	CP10	-0.037
CP03	CP10	-0.040
CP04	CP10	0.039
CP05	CP10	-0.005
CP06	CP10	-0.018
CP07	CP10	0.012
CP08	CP10	0.017
CP01	CP11	-0.004
CP02	CP11	-0.023
CP03	CP11	-0.014
CP04	CP11	-0.022

Sample 1	Sample 2	*r*
CP05	CP11	-0.024
CP06	CP11	-0.037
CP07	CP11	-0.018
CP08	CP11	-0.021
CP10	CP11	-0.016
CP01	CP12	-0.040
CP02	CP12	-0.029
CP03	CP12	0.013
CP04	CP12	-0.009
CP05	CP12	0.048
CP06	CP12	0.012
CP07	CP12	-0.014
CP08	CP12	-0.026
CP10	CP12	0.010
CP11	CP12	0.009
CP01	CP13	0.033
CP02	CP13	-0.019
CP03	CP13	-0.024
CP04	CP13	0.026
CP05	CP13	-0.003
CP06	CP13	0.002
CP07	CP13	-0.030
CP08	CP13	-0.042
CP10	CP13	-0.016
CP11	CP13	0.056
CP12	CP13	0.016
CP01	CP14	0.059
CP02	CP14	-0.041
CP03	CP14	0.000
CP04	CP14	0.022
CP05	CP14	0.002
CP06	CP14	0.029
CP07	CP14	-0.024
CP08	CP14	-0.008
CP10	CP14	-0.019
CP11	CP14	-0.048
CP12	CP14	-0.026
CP13	CP14	-0.014
CP01	CP15	-0.046
CP02	CP15	-0.019
CP03	CP15	-0.029
CP04	CP15	-0.061
CP05	CP15	-0.032

Sample 1	Sample 2	*r*
CP06	CP15	-0.021
CP07	CP15	0.035
CP08	CP15	-0.008
CP10	CP15	-0.058
CP11	CP15	-0.049
CP12	CP15	-0.028
CP13	CP15	-0.052
CP14	CP15	-0.034
CP01	CP16	-0.041
CP02	CP16	-0.012
CP03	CP16	-0.022
CP04	CP16	-0.039
CP05	CP16	0.021
CP06	CP16	-0.036
CP07	CP16	-0.022
CP08	CP16	0.005
CP10	CP16	0.004
CP11	CP16	0.007
CP12	CP16	-0.039
CP13	CP16	-0.014
CP14	CP16	-0.034
CP15	CP16	-0.035
CP01	CP17	-0.026
CP02	CP17	-0.053
CP03	CP17	0.004
CP04	CP17	0.012
CP05	CP17	-0.058
CP06	CP17	-0.048
CP07	CP17	-0.036
CP08	CP17	-0.043
CP10	CP17	-0.010
CP11	CP17	0.022
CP12	CP17	-0.034
CP13	CP17	0.001
CP14	CP17	-0.044
CP15	CP17	-0.021
CP16	CP17	-0.046
CP01	CP18	-0.027
CP02	CP18	0.028
CP03	CP18	-0.030
CP04	CP18	-0.038
CP05	CP18	-0.019
CP06	CP18	-0.019

Sample 1	Sample 2	*r*
CP07	CP18	-0.042
CP08	CP18	0.032
CP10	CP18	-0.023
CP11	CP18	-0.003
CP12	CP18	-0.023
CP13	CP18	-0.027
CP14	CP18	-0.006
CP15	CP18	-0.017
CP16	CP18	0.003
CP17	CP18	-0.050
CP01	CP19	-0.010
CP02	CP19	0.022
CP03	CP19	-0.043
CP04	CP19	-0.004
CP05	CP19	-0.056
CP06	CP19	-0.014
CP07	CP19	-0.007
CP08	CP19	-0.017
CP10	CP19	0.010
CP11	CP19	-0.031
CP12	CP19	-0.018
CP13	CP19	-0.005
CP14	CP19	-0.026
CP15	CP19	-0.035
CP16	CP19	-0.030
CP17	CP19	-0.002
CP18	CP19	-0.038
CP01	CP20	-0.037
CP02	CP20	-0.003
CP03	CP20	-0.078
CP04	CP20	0.004
CP05	CP20	0.046
CP06	CP20	-0.018
CP07	CP20	-0.054
CP08	CP20	-0.057
CP10	CP20	0.002
CP11	CP20	-0.039
CP12	CP20	-0.029
CP13	CP20	-0.037
CP14	CP20	-0.042
CP15	CP20	-0.036
CP16	CP20	-0.027
CP17	CP20	0.033

Sample 1	Sample 2	*r*
CP18	CP20	-0.018
CP19	CP20	-0.022
CP01	CP21	-0.019
CP02	CP21	-0.028
CP03	CP21	-0.003
CP04	CP21	-0.012
CP05	CP21	0.006
CP06	CP21	-0.026
CP07	CP21	-0.064
CP08	CP21	-0.024
CP10	CP21	-0.037
CP11	CP21	-0.060
CP12	CP21	-0.020
CP13	CP21	-0.020
CP14	CP21	-0.026
CP15	CP21	-0.017
CP16	CP21	-0.051
CP17	CP21	-0.029
CP18	CP21	-0.039
CP19	CP21	0.023
CP20	CP21	0.034
CP01	CP22	-0.062
CP02	CP22	-0.027
CP03	CP22	-0.071
CP04	CP22	-0.056
CP05	CP22	-0.028
CP06	CP22	-0.016
CP07	CP22	-0.031
CP08	CP22	-0.045
CP10	CP22	-0.020
CP11	CP22	-0.034
CP12	CP22	-0.007
CP13	CP22	-0.040
CP14	CP22	-0.079
CP15	CP22	-0.025
CP16	CP22	-0.056
CP17	CP22	-0.070
CP18	CP22	0.007
CP19	CP22	-0.035
CP20	CP22	0.052
CP21	CP22	0.006
CP01	CP23	-0.038
CP02	CP23	-0.030

Sample 1	Sample 2	*r*
CP03	CP23	-0.090
CP04	CP23	-0.092
CP05	CP23	0.007
CP06	CP23	-0.049
CP07	CP23	-0.055
CP08	CP23	-0.051
CP10	CP23	-0.052
CP11	CP23	-0.016
CP12	CP23	-0.034
CP13	CP23	-0.012
CP14	CP23	-0.063
CP15	CP23	-0.003
CP16	CP23	-0.074
CP17	CP23	-0.033
CP18	CP23	-0.076
CP19	CP23	-0.083
CP20	CP23	-0.008
CP21	CP23	0.001
CP22	CP23	0.006
CP01	CP24	-0.033
CP02	CP24	-0.041
CP03	CP24	-0.052
CP04	CP24	-0.027
CP05	CP24	-0.016
CP06	CP24	0.070
CP07	CP24	-0.021
CP08	CP24	0.002
CP10	CP24	-0.042
CP11	CP24	-0.015
CP12	CP24	0.011
CP13	CP24	-0.017
CP14	CP24	-0.002
CP15	CP24	-0.050
CP16	CP24	-0.039
CP17	CP24	-0.044
CP18	CP24	-0.033
CP19	CP24	-0.009
CP20	CP24	-0.020
CP21	CP24	-0.012
CP22	CP24	0.001
CP23	CP24	0.008
CP01	CP25	-0.034
CP02	CP25	0.018

Sample 1	Sample 2	*r*
CP03	CP25	-0.031
CP04	CP25	-0.020
CP05	CP25	-0.050
CP06	CP25	-0.030
CP07	CP25	0.070
CP08	CP25	0.133
CP10	CP25	-0.040
CP11	CP25	-0.020
CP12	CP25	-0.022
CP13	CP25	-0.046
CP14	CP25	0.006
CP15	CP25	-0.014
CP16	CP25	-0.015
CP17	CP25	-0.012
CP18	CP25	0.015
CP19	CP25	-0.007
CP20	CP25	-0.056
CP21	CP25	-0.060
CP22	CP25	-0.015
CP23	CP25	-0.074
CP24	CP25	0.009
CP01	CP26	0.009
CP02	CP26	-0.026
CP03	CP26	-0.069
CP04	CP26	0.100
CP05	CP26	-0.047
CP06	CP26	-0.022
CP07	CP26	-0.059
CP08	CP26	-0.042
CP10	CP26	-0.015
CP11	CP26	-0.006
CP12	CP26	-0.012
CP13	CP26	0.017
CP14	CP26	-0.045
CP15	CP26	0.023
CP16	CP26	-0.020
CP17	CP26	-0.014
CP18	CP26	-0.037
CP19	CP26	0.045
CP20	CP26	-0.023
CP21	CP26	-0.026
CP22	CP26	-0.008
CP23	CP26	-0.032

Sample 1	Sample 2	*r*
CP24	CP26	0.009
CP25	CP26	-0.016
CP01	CP27	-0.025
CP02	CP27	0.011
CP03	CP27	0.006
CP04	CP27	-0.017
CP05	CP27	-0.038
CP06	CP27	0.030
CP07	CP27	0.018
CP08	CP27	-0.038
CP10	CP27	-0.002
CP11	CP27	0.012
CP12	CP27	-0.022
CP13	CP27	0.000
CP14	CP27	0.004
CP15	CP27	-0.012
CP16	CP27	-0.024
CP17	CP27	-0.043
CP18	CP27	0.005
CP19	CP27	0.004
CP20	CP27	-0.045
CP21	CP27	-0.035
CP22	CP27	-0.056
CP23	CP27	-0.076
CP24	CP27	0.020
CP25	CP27	-0.028
CP26	CP27	-0.032
CP01	CP28	-0.022
CP02	CP28	-0.068
CP03	CP28	-0.047
CP04	CP28	-0.073
CP05	CP28	-0.022
CP06	CP28	-0.040
CP07	CP28	-0.062
CP08	CP28	-0.083
CP10	CP28	-0.072
CP11	CP28	-0.021
CP12	CP28	-0.039
CP13	CP28	-0.030
CP14	CP28	-0.053
CP15	CP28	-0.087
CP16	CP28	-0.074
CP17	CP28	-0.051

**Sample 1**	**Sample 2**	***r***
CP18	CP28	-0.075
CP19	CP28	-0.081
CP20	CP28	0.046
CP21	CP28	0.015
CP22	CP28	-0.031
CP23	CP28	0.148
CP24	CP28	-0.011
CP25	CP28	-0.091
CP26	CP28	-0.025
CP27	CP28	-0.053
CP01	CP29	-0.025
CP02	CP29	0.038
CP03	CP29	-0.013
CP04	CP29	-0.032
CP05	CP29	0.004
CP06	CP29	-0.046
CP07	CP29	-0.018
CP08	CP29	-0.030
CP10	CP29	-0.031
CP11	CP29	-0.014
CP12	CP29	-0.043
CP13	CP29	-0.011
CP14	CP29	-0.008
CP15	CP29	-0.044
CP16	CP29	0.003
CP17	CP29	-0.067
CP18	CP29	-0.031
CP19	CP29	0.028
CP20	CP29	-0.037
CP21	CP29	-0.021
CP22	CP29	-0.043
CP23	CP29	-0.084
CP24	CP29	-0.063
CP25	CP29	-0.013
CP26	CP29	-0.015
CP27	CP29	-0.024
CP28	CP29	-0.015
CP01	CP30	-0.064
CP02	CP30	0.006
CP03	CP30	0.024
CP04	CP30	-0.046
CP05	CP30	-0.007
CP06	CP30	-0.012

**Sample 1**	**Sample 2**	***r***
CP07	CP30	-0.008
CP08	CP30	0.029
CP10	CP30	-0.053
CP11	CP30	-0.033
CP12	CP30	-0.025
CP13	CP30	-0.050
CP14	CP30	-0.019
CP15	CP30	-0.025
CP16	CP30	-0.044
CP17	CP30	-0.041
CP18	CP30	0.030
CP19	CP30	0.004
CP20	CP30	-0.010
CP21	CP30	0.051
CP22	CP30	0.015
CP23	CP30	-0.074
CP24	CP30	0.018
CP25	CP30	0.034
CP26	CP30	-0.019
CP27	CP30	0.059
CP28	CP30	-0.055
CP29	CP30	-0.055

Table S14 Distributions of *r* values and F_{IS} values for the Siamese crocodile (*Crocodylus siamensis*, Schneider, 1801) [34] and saltwater crocodile (*Crocodylus porosus*, Schneider, 1801) [35].

**Population 1**	**Population 2**	**Relatedness (*r*)**		**Inbreeding coefficient (F_{IS})**	
		**Density**	***p*-value**	**Density**	***p*-value**
All populations	CB [1]	0.076	<0.05	0.432	<0.01
All populations	NR [2]	0.031	0.857	0.144	0.568
All populations	CN [3]	0.029	0.823	0.325	0.314
All populations	NP [4]	0.042	0.191	0.170	<0.01
All populations	CP [5]	0.276	<0.01	0.103	0.904
CB	NR	0.080	0.120	0.400	<0.05
CB	CN	0.082	0.072	0.586	<0.01
CB	NP	0.118	<0.01	0.733	<0.01
CB	CP	0.051	0.636	0.464	<0.05
NR	CN	0.051	0.545	0.294	0.084
NR	NP	0.061	0.228	0.467	<0.01
NR	CP	0.043	0.839	0.214	0.426
NP	CP	0.070	0.137	0.361	<0.05

[1] CB = Chonburi (*Crocodylus siamensis*). [2] NR = Nakhon Ratchasima (*Crocodylus siamensis*). [3] CN = Chainat (*Crocodylus siamensis*). [4] NP = Nakhon Pathom (*Crocodylus siamensis*). [5] CP = Nakhon Ratchasima (*Crocodylus porosus*)

Table S15 Pairwise inbreeding coefficients (F_{IS}) for all 30 Siamese crocodile (*Crocodylus siamensis*, Schneider, 1801) [34] individuals in Chonburi (CB). Detailed information on all Siamese crocodile individuals is presented in Table S1.

**Individual**	F_{IS}
CB01	0.029
CB02	-0.087
CB03	-0.077
CB04	-0.128
CB05	-0.120
CB06	-0.059
CB07	-0.107
CB08	-0.011
CB09	-0.123
CB10	-0.114
CB11	-0.191
CB12	-0.147
CB13	-0.144
CB14	-0.147
CB15	-0.088
CB16	-0.128
CB17	-0.118
CB18	-0.099
CB19	-0.101
CB20	-0.111
CB21	-0.143
CB22	-0.191
CB23	-0.144
CB24	-0.076
CB25	-0.133
CB26	-0.099
CB27	-0.172
CB28	-0.090
CB29	-0.124
CB30	-0.140

Table S16 Pairwise inbreeding coefficients (F_{IS}) for all 30 Siamese crocodile (*Crocodylus siamensis*, Schneider, 1801) [34] individuals in Nakhon Ratchasima (NR). Detailed information on all Siamese crocodile individuals is presented in Table S1.

**Individual**	F_{IS}
NR01	-0.119
NR02	-0.118
NR03	-0.084
NR04	-0.080
NR05	-0.077
NR06	-0.104
NR07	-0.113
NR08	-0.100
NR09	-0.080
NR10	-0.101
NR11	-0.178
NR12	-0.152
NR13	-0.096
NR14	-0.043
NR15	-0.031
NR16	-0.100
NR17	-0.064
NR18	-0.046
NR19	-0.070
NR20	-0.066
NR21	-0.112
NR22	-0.061
NR23	-0.035
NR24	-0.104
NR25	-0.091
NR26	-0.135
NR27	-0.080
NR28	-0.109
NR29	0.008
NR30	-0.034

Table S17 Pairwise inbreeding coefficients (F_{IS}) for all 34 Siamese crocodile (*Crocodylus siamensis*, Schneider, 1801) [34] individuals in Chainat (CN). Detailed information on all Siamese crocodile individuals is presented in Table S1.

Individual	F_{IS}
CN01	-0.059
CN02	-0.084
CN03	-0.073
CN04	-0.052
CN05	-0.096
CN06	-0.068
CN07	-0.088
CN08	0.007
CN09	-0.102
CN10	-0.029
CN11	-0.112
CN12	-0.041
CN13	-0.085
CN14	-0.074
CN15	-0.078
CN16	-0.078
CN17	-0.065
CN18	-0.104
CN19	-0.080
CN20	0.072
CN21	-0.044
CN22	-0.096
CN23	-0.077
CN24	-0.041
CN25	-0.117
CN26	-0.090
CN27	-0.054
CN28	-0.035
CN29	-0.061
CN30	-0.064
CN31	-0.008
CN32	-0.102
CN33	-0.049
CN34	-0.033

Table S18 Pairwise inbreeding coefficients (F_{IS}) for all 42 Siamese crocodile (*Crocodylus siamensis*, Schneider, 1801) [34] individuals in Nakhon Pathom (NP). Detailed information on all Siamese crocodile individuals is presented in Table S1.

**Individual**	F_{IS}	**Individual**	F_{IS}
NP01	-0.071	NP22	-0.004
NP02	-0.042	NP23	-0.072
NP03	0.007	NP24	-0.035
NP04	0.038	NP25	-0.093
NP05	-0.042	NP26	-0.051
NP06	-0.074	NP27	-0.057
NP07	-0.089	NP28	0.085
NP08	-0.054	NP29	-0.004
NP09	-0.078	NP30	-0.091
NP10	-0.035	NP31	-0.079
NP11	-0.063	NP32	-0.086
NP12	-0.052	NP33	-0.073
NP13	-0.062	NP34	0.044
NP14	-0.073	NP35	-0.093
NP15	0.014	NP36	-0.033
NP16	-0.058	NP37	-0.085
NP17	-0.011	NP38	-0.093
NP18	-0.073	NP39	-0.084
NP19	-0.053	NP40	-0.032
NP20	-0.022	NP41	-0.088
NP21	0.028	NP42	-0.026

Table S19 Pairwise inbreeding coefficients (F_{IS}) for all 29 Saltwater crocodile (*Crocodylus porosus*, Schneider, 1801) [35] individuals in Nakhon Ratchasima (CP). Detailed information on all saltwater crocodile individuals is presented in Table S1.

**Individual**	F_{IS}
CP1	-0.117
CP2	-0.112
CP3	-0.052
CP4	-0.046
CP5	-0.087
CP6	-0.086
CP7	-0.059
CP8	-0.105
CP10	-0.093
CP11	-0.090
CP12	-0.098
CP13	-0.089
CP14	-0.112
CP15	-0.075
CP16	0.038
CP17	0.028
CP18	-0.100
CP19	-0.056
CP20	-0.020
CP21	-0.019
CP22	-0.106
CP23	-0.053
CP24	-0.134
CP25	-0.107
CP26	-0.025
CP27	-0.136
CP28	0.070
CP29	0.050
CP30	-0.085

Table S20 Pairwise genetic differentiation (F_{ST}), pairwise F_{ST}^{ENA} values with ENA correction for null alleles and R_{ST} values using FSTAT version 2.9.3 [16] and of Siamese crocodile (*Crocodylus siamensis*, Schneider, 1801) [34] and saltwater crocodile (*Crocodylus porosus*, Schneider, 1801) [35] between captive breeding based on 22 microsatellite loci. The number indicates *p* values, with 110 permutations. Detailed information on all Siamese crocodile and saltwater crocodile individuals is presented in Table S1.

**Combination**	F_{ST}	F_{ST}^{ENA}	R_{ST}
CB [1] x NR [2]	0.064*	0.058	0.097
CB x CN [3]	0.096*	0.094	0.122
CB x NP [4]	0.059*	0.058	0.087
CB x CP [5]	0.172*	0.172	0.308
NR x CN	0.079*	0.079	0.125
NR x NP	0.057*	0.055	0.100
NR x CP	0.174*	0.173	0.270
CN x NP	0.061*	0.063	0.052
CN x CP	0.184*	0.184	0.254
NP x CP	0.143*	0.138	0.270

*p value < 0.05. [1] CB = Chonburi (*Crocodylus siamensis*). [2] NR = Nakhon Ratchasima (*Crocodylus siamensis*). [3] CN = Chainat (*Crocodylus siamensis*). [4] NP = Nakhon Pathom (*Crocodylus siamensis*). [5] CP = Nakhon Ratchasima (*Crocodylus porosus*).

Table S21 Analysis of molecular variance (AMOVA) results for Siamese crocodile (*Crocodylus siamensis*, Schneider, 1801) [34] and saltwater crocodile (*Crocodylus porosus*, Schneider, 1801) [35] based on 22 microsatellite loci using Arlequin version 3.5.2.2 [11]. Detailed information on all Siamese crocodile and saltwater crocodile individuals is presented in Table 1.

Source of variation	df	Sum of squares	Variance components	Percentage of variation
Among populations	4	257.820	0.866	11
Among individual	160	1219.853	0.379	5
Within individual	165	1133.000	6.867	84
Total	329	2610.673	8.111	100

Table S22 Pairwise population Nei's genetic distance (*D*) values using GenAlEx version 6.5 [19] of 166 Siamese crocodile (*Crocodylus siamensis*, Schneider, 1801) [34] and saltwater crocodile (*Crocodylus porosus*, Schneider, 1801) [35] individuals on 22 microsatellite loci. Detailed information on all Siamese crocodile and saltwater crocodile individuals is presented in Table 1.

Nei D	**CB** [1]	**NR** [2]	**CN** [3]	**NP** [4]	**CP** [5]
CB	0.000				
NR	0.147	0.000			
CN	0.202	0.165	0.000		
NP	0.153	0.148	0.141	0.000	
CP	0.609	0.613	0.598	0.604	0.000

[1] CB = Chonburi (*Crocodylus siamensis*). [2] NR = Nakhon Ratchasima (*Crocodylus siamensis*). [3] CN = Chainat (*Crocodylus siamensis*). [4] NP = Nakhon Pathom (*Crocodylus siamensis*). [5] CP = Nakhon Ratchasima (*Crocodylus porosus*).

References

1. Supikamolseni, A.; Ngaoburanawit, N.; Sumontha, M.; Chanhome, L.; Suntrarachun, S.; Peyachoknagul, S.; Srikulnath, K. Molecular barcoding of venomous snakes and species-specific multiplex PCR assay to identify snake groups for which antivenom is available in Thailand. *Genet. Mol. Res*. **2015**, *14*, 13981–13997. https://doi.org/10.4238/2015.
2. Miles, L.G.; Isberg, S.R.; Glenn, T.C.; Lance, S.L.; Dalzell, P.; Thomson, P.C.; Moran, C. A genetic linkage map for the saltwater crocodile (*Crocodylus porosus*). *BMC Genom*. **2009**, *10*, 339. https://doi.org/10.1186/ 1471-2164-10-339.
3. Miles, L.G.; Isberg, S.R.; Moran, C.; Hagen, C.; Glenn, T.C. 253 Novel polymorphic microsatellites for the saltwater crocodile (*Crocodylus porosus*). *Conserv. Genet*. **2009**, *10*, 963–980. https://doi.org/10.1007/s10592-008-9600-7.
4. Lapbenjakul, S.; Thanapa, W.; Twilprawat, P.; Muangmai, N.; Khancanaketu, T.; Temsiripong, Y.; Unajak, S.; Peyachoknagul, S.; Srikulnath, K. High genetic diversity and demographic history of captive Siamese and saltwater crocodiles suggest the first step toward the establishment of a breeding and reintroduction program in Thailand. *PLoS One* **2017**, *12*, e0184526. https://doi.org/10.1371/journal.pone.0184526.
5. Ariyaraphong, N.; Pansrikaew, T.; Jangtarwan, K.; Thintip, J.; Singchat, W.; Laopichienpong, N.; Pongsanarm, T.; Panthum, T.; Suntronpong, A.; Ahmad, S.F.; et al. Introduction of wild Chinese gorals into a captive population requires careful genetic breeding plan monitoring for successful long-term conservation. *Glob. Ecol. Conserv*. **2021**, *28*, e01675. https://doi.org/10.1016/j.gecco.2021.e01675.
6. Chailertrit, V.; Swatdipong, A.; Peyachoknagul, S.; Salaenoi, J.; Srikulnath, K. Isolation and characterization of novel microsatellite markers from Siamese fighting fish (*Betta splendens*, Osphronemidae, Anabantoidei) and their transferability to related species, *B. smaragdina* and *B. imbellis*. *Genet. Mol. Res*. **2014**, *13*, 7157–7162. https://doi.org/10.4238/2014.September.5.1.
7. Jangtarwan, K.; Koomgun, T.; Prasongmaneerut, T.; Thongchum, R.; Singchat, W.; Tawichasri, P.; Fukayama, T.; Sillapaprayoon, S.; Kraichak, E.; Muangmai, N.; et al. Take one step backward to move forward: Assessment of genetic diversity and population structure of captive Asian woolly-necked storks (*Ciconia episcopus*). *PLoS One* **2019**, *14*, e0223726. https://doi.org/10.1371/journal.pone.0223726.
8. Jangtarwan, K.; Kamsongkram, P.; Subpayakom, N.; Sillapaprayoon, S.; Muangmai, N.; Kongphoemph, A.; Wongsodchuen, A.; Intapan, S.; Chamchumroon, W.; Safoowong, M.; et al. Predictive genetic plan for a captive population of the Chinese goral (*Naemorhedus griseus*) and prescriptive action for *ex situ* and *in situ* conservation management in Thailand. *PLoS One* **2020**, *15*, e0234064. https://doi.org/10.1371/journal.pone.0234064.
9. Thintip, J.; Singchat, W.; Ahmad, S.F.; Ariyaraphong, N.; Muangmai, N.; Chamchumroon, W.; Pitiwong, K.; Suksavate, W.; Duangjai, S.; Duengkae, P.; et al. Reduced genetic variability in a captive-bred population of the endangered Hume's pheasant (*Syrmaticus humiae*, Hume 1881) revealed by microsatellite genotyping and D-loop sequencing. *PLoS One* **2021**, *16*, e0256573. https://doi.org/10.1371/journal.pone.0256573.
10. Wongtienchai, P.; Lapbenjakul, S.; Jangtarwan, K.; Areesirisuk, P.; Mahaprom, R.; Subpayakom, N.; Singchat, W.; Sillapaprayoon, S.; Muangmai, N.; Songchan, R.; et al. Genetic management of a water monitor lizard (*Varanus salvator macromaculatus*) population at Bang Kachao Peninsula as a consequence of urbanization with Varanus Farm Kamphaeng Saen as the first captive research establishment. *J. Zool. Syst. Evol. Res*. **2021**, *59*, 484–497. https://doi.org/10.1111/jzs.12436.
11. Excoffier, L.; Lischer, H.E. Arlequin suite ver 3.5: a new series of programs to perform population genetics analyses under Linux and Windows. *Mol. Ecol. Resour.* **2010**, *10*, 564–567. https://doi.org/10.1111/j.1755-0998.2010.02847.x.
12. Guo, S.W.; Thompson, E.A. Performing the exact test of Hardy-Weinberg proportion for multiple alleles. *Biometrics* **1992**, *48*, 361–372. https://doi.org/10.2307/2532296.

13. Raymond, M.; Rousset, F. GENEPOP (version 1.2): population genetics software for exact tests and ecumenicism. *J. Hered*. **1995**, 86, 248–249. https://doi.org/10.1093/oxfordjournals.jhered. a111573.
14. R Core Team. *R: A Language and Environment for Statistical Computing*; R Foundation for Statistical Computing: Vienna, Austria, 2022.
15. Welch, B.L. The generalization of student's' problem when several different population variances are involved. *Biometrika* **1947**, *34*, 28–35. https://doi.org/10.1093/biomet/34.1-2.28.
16. Goudet, J.F. FSTAT (version 1.2): A computer program to calculate F-statistics. *J. Hered*. **1995**, *86*, 485–486. https://doi.org/10.1093/oxfordjournals.jhered.a111627.
17. Van, O.C.; Hutchinson, W.F.; Wills, D.P.; Shipley, P. MICRO-CHECKER: software for identifying and correcting genotyping errors in microsatellite data. *Mol. Ecol. Notes*. **2004**, *4*, 535–538. https://doi.org/10.1111/ j.1471-8286.2004.00684.x.
18. Park, S.D.E. Trypanotolerance in West African cattle and the population genetic effects of selection. *PhD thesis*. University of Dublin, Ireland, 2001.
19. Peakall, R.; Smouse, P.E. GenAlEx 6.5: Genetic analysis in Excel. Population genetic software for teaching and research—An update. *Bioinformatics* **2012**, *28*, 2537–2539. https://doi.org/ 10.1093/bioinformatics/bts460.
20. Do, C.; Waples, R.S.; Peel, D.; Macbeth, G.M.; Tillett, B.J.; Ovenden, J.R. NeEstimator v2: Re-implementation of software for the estimation of contemporary effective population size (N_e) from genetic data. *Mol. Ecol. Resour.* **2014**, *14*, 209–214. https://doi.org/10.1111/1755-0998.12157.
21. Præstgaard, J.T. Permutation and bootstrap Kolmogorov-Smirnov tests for the equality of two distributions. *Scand. J. Stat*. **1995**, *22*, 305–322.
22. Lynch, M.; Ritland, K. Estimation of pairwise relatedness with molecular markers. *Genetics* **1999**, *152*, 1753–1766.
23. Wang, J. COANCESTRY: a program for simulating, estimating and analysing relatedness and inbreeding coefficients. *Mol. Ecol. Resour.* **2011**, *11*, 141–145. https://doi.org/10.1111/j.1755-0998.2010.
24. Chapuis, M.P.; Estoup, A. Microsatellite null alleles and estimation of population differentiation. *Mol. Biol. Evol*. **2007**, *24*, 621–631. https://doi.org/10.1093/molbev/MSL191.
25. Nei, M. Genetic distance between populations. *Am. Nat.* **1972**, *106*, 283–292. https://doi.org/10.1086/ 282771.
26. Pritchard, J.K.; Stephens, M.; Donnelly, P. Inference of population structure using multilocus genotype data. *Genetics* **2000**, 155, 945–959.
27. Earl, D.A. STRUCTURE HARVESTER: a website and program for visualizing STRUCTURE output and implementing the Evanno method. *Conserv. Genet. Resour*. **2012**, *4*, 359–361. https://doi.org/10.1007/ s12686-011-9548-7.
28. Tamura, K.; Stecher, G.; Kumar, S. MEGA11: Molecular evolutionary genetics analysis version 11. *Mol. Biol. Evol.* **2021**, *38*, 3022–3027. https://doi.org/10.1093/molbev/msab120.
29. Rozas, J.; Ferrer-Mata, A.; Sánchez-DelBarrio, J.C.; Guirao-Rico, S.; Librado, P.; Ramos-Onsins, S.E.; Sanchez-Gracia, A. DnaSP6: DNA sequence polymorphism analysis of large data sets. *Mol. Biol. Evol.* **2017**, *34*, 3299–3302.
30. Weir, B.; Cockerham, C. Estimating F-Statistics for the analysis of population-structure. *Evolution* **1984**, *38*, 1358–1370. https://doi. org/10.2307/2408641.
31. Excoffier, L.; Smouse, P.E.; Quattro, J.M. Analysis of molecular variance inferred from metric distances among DNA haplotypes: Application to human mitochondrial DNA restriction data. *Genetics* **1992**, *131*, 479–491.
32. Clement, M.; Snell, Q.; Walker, P.; Posada, D.; Crandall, K. TCS: Estimating gene genealogies. *Parallel and Distributed Processing Symposium, International Proceedings*. 2002, *2*, 184.
33. Huelsenbeck, J.P.; Ronquist, F.; MRBAYES: Bayesian inference of phylogenetic trees. *Bioinformatics* **2001**, *17*, 754–755. https://doi.org/10.1093/bioinformatics/17.8.754.
34. Schneider, J.G. *Crocodylus siamensis.* 1801. Available online: https://www.gbif.org/species/185108439 (accessed on 16 January 2023).

35. Schneider. *Crocodylus porosus.* 1801. Available online: https://www.gbif.org/species/ 144104483 (accessed on 16 January 2023).
